# Supplementary material for: Engineering Escherichia coli to Utilize Erythritol as Sole Carbon Source
Source: Adv Sci (Weinh). 2023 Mar 20;10(14):2207008. doi: 10.1002/advs.202207008 (PMC10190533; doi:10.1002/advs.202207008)
Supplement: Supplementary file 1 — Supporting Information [file ADVS-10-2207008-s001.pdf]

ADVANCED SCIENCE
Open Access

Supporting Information

for *Adv. Sci.*, DOI 10.1002/advs.202207008

Engineering *Escherichia coli* to Utilize Erythritol as Sole Carbon Source

*Fang Ba, Xiangyang Ji, Shuhui Huang, Yufei Zhang, Wan-Qiu Liu, Yifan Liu, Shengjie Ling and Jian Li**

Supplementary Information

Engineering *Escherichia coli* to Utilize Erythritol as Sole Carbon Source

Fang Ba, Xiangyang Ji, Shuhui Huang, Yufei Zhang, Wan-Qiu Liu, Yifan Liu, Shengjie Ling, and Jian Li*

School of Physical Science and Technology, ShanghaiTech University, Shanghai, 201210, China
*Corresponding author. E-mail: lijian@shanghaitech.edu.cn (J.L.)

I. Supplementary Tables

Supplementary Table 1. *E. coli* strains......3
Supplementary Table 2. Genetic parts......4
Supplementary Table 3. Gene clusters......22
Supplementary Table 4. Vectors......28
Supplementary Table 5. Plasmids......28
Supplementary Table 6. Primers......33

II. Supplementary Results

Supplementary Figure 1. Isolation of microorganisms growing in the erythritol-based M9 medium......34
Supplementary Figure 2. Characterization of the isolated *Ochrobactrum* spp. strain......35
Supplementary Figure 3. Annotation of erythritol catabolism-associated genes......36
Supplementary Figure 4. Nucleotide BLAST alignments of erythritol catabolism-associated genes......37
Supplementary Figure 5. Protein BLAST alignments of erythritol catabolism-associated genes......38
Supplementary Figure 6. *In vivo* expression of erythritol catabolism-associated genes......39
Supplementary Figure 7. Native promoter-RBS (pR-) composite part characterization in the erythritol gene cluster......40
Supplementary Figure 8. Native RBS characterization in the erythritol gene cluster......41
Supplementary Figure 9. Prediction of eryE and eryG transmembrane topology by TMHMM - 2.0......42
Supplementary Figure 10. eryE-sfGFP and eryG-sfGFP characterization......43
Supplementary Figure 11. Comparison of cell growth between two strains with and without the erythritol ABC-transporter (i.e., eryE/F/G)......44
Supplementary Figure 12. pEryR characterization......45
Supplementary Figure 13. pEryF characterization......47
Supplementary Figure 14. eryD and eryR characterization......49
Supplementary Figure 15. The first step for characterization of eryD binding site prefix......51
Supplementary Figure 16. The second step for characterization of eryD binding site prefix......52
Supplementary Figure 17. The third step for characterization of eryD binding site prefix......54
Supplementary Figure 18. The first step for characterization of eryD binding site suffix......56
Supplementary Figure 19. The second step for characterization of eryD binding site suffix......58
Supplementary Figure 20. The third step for characterization of eryD binding site suffix......60
Supplementary Figure 21. EMSA analysis of eryD-eryO interaction......61
Supplementary Figure 22. pEry2 characterization......62
Supplementary Figure 23. Structure models of eryD monomer and homotetramer......64
Supplementary Figure 24. eryD assembled as a homotetramer......65
Supplementary Figure 25. Erythritol induction of pEryF-732 at 30°C and 16°C......66
Supplementary Figure 26. Erythritol induction of synthetic operons at 30°C and 16°C......68

Supplementary Figure 27. Sample preparation for mRNA transcriptional analysis……………………………………………70
Supplementary Figure 28. Volcano plot of differentially expressed genes from mRNA transcriptional analysis……71
Supplementary Figure 29. mRNA transcriptional analysis…………………………………………………………………72
Supplementary Figure 30. Some up-regulated gene clusters (RNA-seq) from M9-erythritol catabolic *E. coli* strain……74
Supplementary Figure 31. glcC can respond to erythritol catabolism……………………………………………………75
Supplementary Figure 32. Erythritol ABC transporter cluster changes *E. coli* morphology……………………………76
Supplementary Figure 33. Effect of eryE, eryF, and eryG on cell morphology……………………………………………78
Supplementary Figure 34. Potential endogenous erythritol transporter homologous screening………………………79
Supplementary Figure 35. Several eryG homologous permeases in *E. coli* may facilitate erythritol catabolism……80
Supplementary Figure 36. Up-regulated carbohydrate transporters were not essential for erythritol transport (from COG analysis of RNA-seq) ……………………………………………………………………………………81
Supplementary Figure 37. Erythritol catabolic *E. coli* strain utilizes different carbon sources…………………………82
Supplementary Figure 38. Growth curves of engineered *E. coli* Nissle 1917 in SIF with additional erythritol……83
Supplementary Figure 39. Additional erythritol catabolism promotes *E. coli* growth in LB medium…………………84
Supplementary Figure 40. *E. coli* MG1655 with the erythritol catabolic cluster could express green fluorescent protein in M9-erythritol medium……………………………………………………………………………………85
Supplementary Figure 41. Standard curve of erythritol analyzed by HPLC (Refractive Index Detector, RID) ……86

III. Supplementary References

References……87

I. Supplementary Tables

Supplementary Table 1. *E. coli* strains.

Strain name	Purpose	Genotype	Origin
Mach1-T1	Molecular cloning, promoter characterization, ribosome binding site characterization	F^- φ80(*lac*Z)Δ*M15* Δ*lac*X74 *hsd*R(r_K^- m_K^+) Δ*rec*A1398 *end*A1 *ton*A	TransGen Biotech
MG1655	Erythritol catabolism characterization, host strain of RNA-Seq	K12 F^- *lambda- ilvG- rfb-50 rph-1*	Shanghai Weidi Biotechnology
BL21(DE3)	Promoter characterization, ribosome binding site characterization	F^- *omp*T *hsd*S_B(r_B^- m_B^-) *gal dcm*(DE3)	TransGen Biotech
DH5α λpir	Cloning R6K-ori plasmids	F^- φ80 *lac*ZΔM15 Δ(*lac*ZYA-*arg* F) *LAMpir* U169 *end*A1 *rec*A1 *hsd*R17(r_k^-,m_k^+) *sup*E44λ- *thi* -1 *gyr*A96 *rel*A1 *pho*A	Shanghai Weidi Biotechnology
Nissle 1917 (EcN)	Host strain of simulated intestinal fluid experiment		Mutaflor

All *E. coli* strains used in this study are listed above.

Supplementary Table 2. Genetic parts.

Part name	Type	DNA sequence	Reference
J23100	promoter	ttgacggctagctcagtcctaggtacagtgctagc	1, BBa_J23100
J23105	promoter	tttacggctagctcagtcctaggtactatgctagc	1, BBa_J23105
J23106	promoter	tttacggctagctcagtcctaggtatagtgctagc	1, BBa_J23106
J23109	promoter	tttacagctagctcagtcctagggactgtgctagc	1, BBa_J23109
J23113	promoter	ctgatggctagctcagtcctagggattatgctagc	1, BBa_J23113
J23114	promoter	tttatggctagctcagtcctaggtacaatgctagc	1, BBa_J23114
pEryF-732	promoter	tatcaaacgccttcagcaattctgccgtattgtcgttatggaaacggatgatgcggctgaattagaccgcccggagattaccctcgaccattgtcatcacgcttaccggataggcaaaagcctccgccaaagcggtgcaggacgcgacagacgagatcatgatagcctccacatgaattccagggccccgctcctccgtgcatcctggaaacagatgcgccgcaatcaaaacctatgatagcggcgtgttattaactccaataatgatgttataaagctcgaaagctgtcaatcggatgttttcgaaacaccggtgaaatagcacaatttcgcgccgaatctcgatcacgatctagagaaatccactacataattcgttatcaatcgcactattcacagcaccgtcttttctcgcaaagccagccacaaagatactcacaaacttctatattttcttttattttcaatgaattaaacatatttaaaaccgaagaaacattaagctattgatggaaaacagtgggaaaaccgcactcgaaggcgcggagatttcccctacattcactctttggtgaaaaaaaatgcgccatctagaaaattttacagacaacgtgatagcgttatgttatctccagcatacgccatcgcccgatttcattataaacagggctgcgagcgacgtattttcggtcgcgggaggaatccaaatgtcaatgaaagcacatagccgcc	This work
pEryF-632	promoter	gcttaccggataggcaaaagcctccgccaaagcggtgcaggacgcgacagacgagatcatgatagcctccacatgaattccagggccccgctcctccgtgcatcctggaaacagatgcgccgcaatcaaaacctatgatagcggcgtgttattaactccaataatgatgttataaagctcgaaagctgtcaatcggatgttttcgaaacaccggtgaaatagcacaatttcgcgccgaatctcgatcacgatctagagaaatccactacataattcgttatcaatcgcactattcacagcaccgtcttttctcgcaaagccagccacaaagatactcacaaacttctatattttcttttattttcaatgaattaaacatatttaaaaccgaagaaacattaagctattgatggaaaacagtgggaaaaccgcactcgaaggcgcggagatttcccctacattcactctttggtgaaaaaaaatgcgccatctagaaaattttacagacaacgtgatagcgttatgttatctccagcatacgccatcgcccgatttcattataaacagggctgcgagcgacgtattttcggtcgcgggaggaatccaaatgtcaatgaaagcacatagccgcc	This work
pEryF-532	promoter	catcctggaaacagatgcgccgcaatcaaaacctatgatagcggcgtgttattaactccaataatgatgttataaagctcgaaagctgtcaatcggatgttttcgaaacaccggtgaaatagcacaatttcgcgccgaatctcgatcacgatctagagaaatccactacataattcgttatcaatcgcactattcacagcaccgtcttttctcgcaaagccagccacaaagatactcacaaacttctatattttcttttattttcaatgaattaaacatatttaaaaccgaagaaacattaagctattgatggaaaacagtgggaaaaccgcactcgaaggcgcggagatttcccctacattcactctttggtgaaaaaaaatgcgccatctagaaaattttacagacaacgtgatagcgttatgttatctccagcatacgccatcgcccgatttcattataaacagggctgcgagcgacgtattttcggtcgcgggaggaatccaaatgtcaatgaaagcacatagccgcc	This work
pEryF-432	promoter	tttcgaaacaccggtgaaatagcacaatttcgcgccgaatctcgatcacgatctagagaaatccactacataattcgttatcaatcgcactattcacagcaccgtcttttctcgcaaagccagccacaaagatactcacaaacttctatattttcttttattttcaatgaattaaacatatttaaaaccgaagaaacattaagctattgatggaaaacagtgggaaaaccgcactcgaaggcgcggagatttcccctacattcactctttggtgaaaaaaaatgcgccatctagaaaattttacagacaacgtgatagcgttatgttatctccagcatacgccatcgcccgatttcattataaacagggctgcgagcgacgtattttcggtcgcgggaggaatccaaatgtcaatgaaagcacatagccgcc	This work
pEryF-332	promoter	accgtcttttctcgcaaagccagccacaaagatactcacaaacttctatattttcttttattttcaatgaattaaacatatttaaaaccgaagaaacattaagctattgatggaaaacagtgggaaaaccgcactcgaaggcgcggagatttcccctacattcactctttggtgaaaaaaaatgcgccatctagaaaattttacagacaacgtgat	This work

		agcgttatgttatctccagcatacgccatcgcccgatttcattataaacagggctgcgagcgacgtattttcggtcgcgggaggaatccaaatgtcaatgaaagcacatagccgcc	
pEryF-232	promoter	aagctattgatggaaaacagtgggaaaaccgcactcgaaggcgcggagatttcccctacattcactctttggtgaaaaaaaatgcgccatctagaaaattttacagacaacgtgatagcgttatgttatctccagcatacgccatcgcccgatttcattataaacagggctgcgagcgacgtattttcggtcgcgggaggaatccaaatgtcaatgaaagcacatagccgcc	This work
pEryF-222	promoter	tggaaaacagtgggaaaaccgcactcgaaggcgcggagatttcccctacattcactctttggtgaaaaaaaatgcgccatctagaaaattttacagacaacgtgatagcgttatgttatctccagcatacgccatcgcccgatttcattataaacagggctgcgagcgacgtattttcggtcgcgggaggaatccaaatgtcaatgaaagcacatagccgcc	This work
pEryF-212	promoter	tgggaaaaccgcactcgaaggcgcggagatttcccctacattcactctttggtgaaaaaaaatgcgccatctagaaaattttacagacaacgtgatagcgttatgttatctccagcatacgccatcgcccgatttcattataaacagggctgcgagcgacgtattttcggtcgcgggaggaatccaaatgtcaatgaaagcacatagccgcc	This work
pEryF-202	promoter	gcactcgaaggcgcggagatttcccctacattcactctttggtgaaaaaaaatgcgccatctagaaaattttacagacaacgtgatagcgttatgttatctccagcatacgccatcgcccgatttcattataaacagggctgcgagcgacgtattttcggtcgcgggaggaatccaaatgtcaatgaaagcacatagccgcc	This work
pEryF-192	promoter	gcgcggagatttcccctacattcactctttggtgaaaaaaaatgcgccatctagaaaattttacagacaacgtgatagcgttatgttatctccagcatacgccatcgcccgatttcattataaacagggctgcgagcgacgtattttcggtcgcgggaggaatccaaatgtcaatgaaagcacatagccgcc	This work
pEryF-182	promoter	ttcccctacattcactctttggtgaaaaaaaatgcgccatctagaaaattttacagacaacgtgatagcgttatgttatctccagcatacgccatcgcccgatttcattataaacagggctgcgagcgacgtattttcggtcgcgggaggaatccaaatgtcaatgaaagcacatagccgcc	This work
pEryF-172	promoter	ttcactctttggtgaaaaaaaatgcgccatctagaaaattttacagacaacgtgatagcgttatgttatctccagcatacgccatcgcccgatttcattataaacagggctgcgagcgacgtattttcggtcgcgggaggaatccaaatgtcaatgaaagcacatagccgcc	This work
pEryF-162	promoter	ggtgaaaaaaaatgcgccatctagaaaattttacagacaacgtgatagcgttatgttatctccagcatacgccatcgcccgatttcattataaacagggctgcgagcgacgtattttcggtcgcgggaggaatccaaatgtcaatgaaagcacatagccgcc	This work
pEryF-161	promoter	cgtgaaaaaaaatgcgccatctagaaaattttacagacaacgtgatagcgttatgttatctccagcatacgccatcgcccgatttcattataaacagggctgcgagcgacgtattttcggtcgcgggaggaatccaaatgtcaatgaaagcacatagccgcc	This work
pEryF-160	promoter	cctgaaaaaaaatgcgccatctagaaaattttacagacaacgtgatagcgttatgttatctccagcatacgccatcgcccgatttcattataaacagggctgcgagcgacgtattttcggtcgcgggaggaatccaaatgtcaatgaaagcacatagccgcc	This work
pEryF-159	promoter	cccgaaaaaaaatgcgccatctagaaaattttacagacaacgtgatagcgttatgttatctccagcatacgccatcgcccgatttcattataaacagggctgcgagcgacgtattttcggtcgcgggaggaatccaaatgtcaatgaaagcacatagccgcc	This work
pEryF-158	promoter	ccctaaaaaaaatgcgccatctagaaaattttacagacaacgtgatagcgttatgttatctccagcatacgccatcgcccgatttcattataaacagggctgcgagcgacgtattttcggtcgcgggaggaatccaaatgtcaatgaaagcacatagccgcc	This work
pEryF-157	promoter	cccttaaaaaaatgcgccatctagaaaattttacagacaacgtgatagcgttatgttatctccagcatacgccatcgcccgatttcattataaacagggctgcgagcgacgtattttcggtcgcgggaggaatccaaatgtcaatgaaagcacatagccgcc	This work
pEryF-156	promoter	ccctttaaaaaatgcgccatctagaaaattttacagacaacgtgatagcgttatgttatctccagcatacgccatcgcccgatttcattataaacagggctgcgagcgacgtattttcggtcgcgggaggaatccaaatgtcaatgaaagcacatagccgcc	This work

pEryF-155	promoter	ccctttcaaaaatgcgccatctagaaaattttacagacaacgtgatagcgttatgttatctccagcatacgccatcgcccgatttcattataaacagggctgcgagcgacgtattttcggtcgcgggaggaatccaaatgtcaatgaaagcacatagccgcc	This work
pEryF-154	promoter	ccctttccaaaatgcgccatctagaaaattttacagacaacgtgatagcgttatgttatctccagcatacgccatcgcccgatttcattataaacagggctgcgagcgacgtattttcggtcgcgggaggaatccaaatgtcaatgaaagcacatagccgcc	This work
pEryF-153	promoter	ccctttcccaaatgcgccatctagaaaattttacagacaacgtgatagcgttatgttatctccagcatacgccatcgcccgatttcattataaacagggctgcgagcgacgtattttcggtcgcgggaggaatccaaatgtcaatgaaagcacatagccgcc	This work
pEryF-152	promoter	aatgcgccatctagaaaattttacagacaacgtgatagcgttatgttatctccagcatacgccatcgcccgatttcattataaacagggctgcgagcgacgtattttcggtcgcgggaggaatccaaatgtcaatgaaagcacatagccgcc	This work
pEryF-142	promoter	ctagaaaattttacagacaacgtgatagcgttatgttatctccagcatacgccatcgcccgatttcattataaacagggctgcgagcgacgtattttcggtcgcgggaggaatccaaatgtcaatgaaagcacatagccgcc	This work
pEryF-132	promoter	ttacagacaacgtgatagcgttatgttatctccagcatacgccatcgcccgatttcattataaacagggctgcgagcgacgtattttcggtcgcgggaggaatccaaatgtcaatgaaagcacatagccgcc	This work
pEryF-122	promoter	cgtgatagcgttatgttatctccagcatacgccatcgcccgatttcattataaacagggctgcgagcgacgtattttcggtcgcgggaggaatccaaatgtcaatgaaagcacatagccgcc	This work
pEryF-112	promoter	ttatgttatctccagcatacgccatcgcccgatttcattataaacagggctgcgagcgacgtattttcggtcgcgggaggaatccaaatgtcaatgaaagcacatagccgcc	This work
pEryF-102	promoter	tccagcatacgccatcgcccgatttcattataaacagggctgcgagcgacgtattttcggtcgcgggaggaatccaaatgtcaatgaaagcacatagccgcc	This work
pEryF-159-10	promoter	tatcaaacgccttcagcaattctgccgtattgtcgttatggaaacggatgatgcggctgaattagaccgcccggagattaccctcgaccattgtcatcacgcttaccggataggcaaaagcctccgccaaagcggtgcaggacgcgacagacgagatcatgatagcctccacatgaattccagggccccgctcctccgtgcatcctggaaacagatgcgccgcaatcaaaacctatgatagcggcgtgttattaactccaataatgatgttataaagctcgaaagctgtcaatcggatgttttcgaaacaccggtgaaatagcacaatttcgcgccgaatctcgatcacgatctagagaaatccactacataattcgttatcaatcgcactattcacagcaccgtcttttctcgcaaagccagccacaaagatactcacaaacttctatattttcttttattttcaatgaattaaacatatttaaaaccgaagaaacattaagctattgatggaaaacagtgggaaaaccgcactcgaaggcgcggagatttcccctacattcactctttggtgaaaaaaaatgcgccatctagaaaattttacagacaacgtgatagcgttatgttatctccagcatacgccatcgcccgatttcattataaacagggctgcgagcgacgtattttcggtcgcgggaggaatccaaatgtcaatgaaagca	This work
pEryF-159-20	promoter	tatcaaacgccttcagcaattctgccgtattgtcgttatggaaacggatgatgcggctgaattagaccgcccggagattaccctcgaccattgtcatcacgcttaccggataggcaaaagcctccgccaaagcggtgcaggacgcgacagacgagatcatgatagcctccacatgaattccagggccccgctcctccgtgcatcctggaaacagatgcgccgcaatcaaaacctatgatagcggcgtgttattaactccaataatgatgttataaagctcgaaagctgtcaatcggatgttttcgaaacaccggtgaaatagcacaatttcgcgccgaatctcgatcacgatctagagaaatccactacataattcgttatcaatcgcactattcacagcaccgtcttttctcgcaaagccagccacaaagatactcacaaacttctatattttcttttattttcaatgaattaaacatatttaaaaccgaagaaacattaagctattgatggaaaacagtgggaaaaccgcactcgaaggcgcggagatttcccctacattcactctttggtgaaaaaaaatgcgccatctagaaaattttacagacaacgtgatagcgttatgttatctccagcatacgccatcgcccgatttcattataaacagggctgcgagcgacgtattttcggtcgcgggaggaatccaaatgtc	This work
pEryF-159-30	promoter	tatcaaacgccttcagcaattctgccgtattgtcgttatggaaacggatgatgcggctgaattagaccgcccggagattaccctcgaccattgtcatcacgcttaccggataggcaaaagcctccgccaaagcggtgcaggacgcgacagacgagatcatgatagcctccacatgaattccagggccccgctcctccgtgcatcctggaa	This work

		acagatgcgccgcaatcaaaacctatgatagcggcgtgttattaactccaataatgatgttataaagctcg<br>aaagctgtcaatcggatgttttcgaaacaccggtgaaatagcacaatttcgcgccgaatctcgatcacgat<br>ctagagaaatccactacataattcgttatcaatcgcactattcacagcaccgtcttttctcgcaaagccagcc<br>acaaagatactcacaaacttctatattttcttttattttcaatgaattaaacatatttaaaaccgaagaaacatta<br>agctattgatggaaaacagtgggaaaaccgcactcgaaggcgcggagatttcccctacattcactctttgg<br>tgaaaaaaaatgcgccatctagaaaattttacagacaacgtgatagcgttatgttatctccagcatacgcc<br>atcgcccgatttcattataaacagggctgcgagcgacgtattttcggtcgcgggaggaa	
pEryF-159-40	promoter	tatcaaacgccttcagcaattctgccgtattgtcgttatggaaacggatgatgcggctgaattagaccgccc<br>ggagattaccctcgaccattgtcatcacgcttaccggataggcaaaagcctccgccaaagcggtgcagg<br>acgcgacagacgagatcatgatagcctccacatgaattccagggccccgctcctccgtgcatcctggaa<br>acagatgcgccgcaatcaaaacctatgatagcggcgtgttattaactccaataatgatgttataaagctcg<br>aaagctgtcaatcggatgttttcgaaacaccggtgaaatagcacaatttcgcgccgaatctcgatcacgat<br>ctagagaaatccactacataattcgttatcaatcgcactattcacagcaccgtcttttctcgcaaagccagcc<br>acaaagatactcacaaacttctatattttcttttattttcaatgaattaaacatatttaaaaccgaagaaacatta<br>agctattgatggaaaacagtgggaaaaccgcactcgaaggcgcggagatttcccctacattcactctttgg<br>tgaaaaaaaatgcgccatctagaaaattttacagacaacgtgatagcgttatgttatctccagcatacgcc<br>atcgcccgatttcattataaacagggctgcgagcgacgtattttcggtc	This work
pEryF-159-50	promoter	tatcaaacgccttcagcaattctgccgtattgtcgttatggaaacggatgatgcggctgaattagaccgccc<br>ggagattaccctcgaccattgtcatcacgcttaccggataggcaaaagcctccgccaaagcggtgcagg<br>acgcgacagacgagatcatgatagcctccacatgaattccagggccccgctcctccgtgcatcctggaa<br>acagatgcgccgcaatcaaaacctatgatagcggcgtgttattaactccaataatgatgttataaagctcg<br>aaagctgtcaatcggatgttttcgaaacaccggtgaaatagcacaatttcgcgccgaatctcgatcacgat<br>ctagagaaatccactacataattcgttatcaatcgcactattcacagcaccgtcttttctcgcaaagccagcc<br>acaaagatactcacaaacttctatattttcttttattttcaatgaattaaacatatttaaaaccgaagaaacatta<br>agctattgatggaaaacagtgggaaaaccgcactcgaaggcgcggagatttcccctacattcactctttgg<br>tgaaaaaaaatgcgccatctagaaaattttacagacaacgtgatagcgttatgttatctccagcatacgcc<br>atcgcccgatttcattataaacagggctgcgagcgacgt	This work
pEryF-159-60	promoter	tatcaaacgccttcagcaattctgccgtattgtcgttatggaaacggatgatgcggctgaattagaccgccc<br>ggagattaccctcgaccattgtcatcacgcttaccggataggcaaaagcctccgccaaagcggtgcagg<br>acgcgacagacgagatcatgatagcctccacatgaattccagggccccgctcctccgtgcatcctggaa<br>acagatgcgccgcaatcaaaacctatgatagcggcgtgttattaactccaataatgatgttataaagctcg<br>aaagctgtcaatcggatgttttcgaaacaccggtgaaatagcacaatttcgcgccgaatctcgatcacgat<br>ctagagaaatccactacataattcgttatcaatcgcactattcacagcaccgtcttttctcgcaaagccagcc<br>acaaagatactcacaaacttctatattttcttttattttcaatgaattaaacatatttaaaaccgaagaaacatta<br>agctattgatggaaaacagtgggaaaaccgcactcgaaggcgcggagatttcccctacattcactctttgg<br>tgaaaaaaaatgcgccatctagaaaattttacagacaacgtgatagcgttatgttatctccagcatacgcc<br>atcgcccgatttcattataaacagggctg	This work
pEryF-159-70	promoter	tatcaaacgccttcagcaattctgccgtattgtcgttatggaaacggatgatgcggctgaattagaccgccc<br>ggagattaccctcgaccattgtcatcacgcttaccggataggcaaaagcctccgccaaagcggtgcagg<br>acgcgacagacgagatcatgatagcctccacatgaattccagggccccgctcctccgtgcatcctggaa<br>acagatgcgccgcaatcaaaacctatgatagcggcgtgttattaactccaataatgatgttataaagctcg<br>aaagctgtcaatcggatgttttcgaaacaccggtgaaatagcacaatttcgcgccgaatctcgatcacgat<br>ctagagaaatccactacataattcgttatcaatcgcactattcacagcaccgtcttttctcgcaaagccagcc<br>acaaagatactcacaaacttctatattttcttttattttcaatgaattaaacatatttaaaaccgaagaaacatta<br>agctattgatggaaaacagtgggaaaaccgcactcgaaggcgcggagatttcccctacattcactctttgg<br>tgaaaaaaaatgcgccatctagaaaattttacagacaacgtgatagcgttatgttatctccagcatacgcc	This work

		atcgcccgatttcattata	
pEryF-159-80	promoter	tatcaaacgccttcagcaattctgccgtattgtcgttatggaaacggatgatgcggctgaattagaccgccc<br>ggagattaccctcgaccattgtcatcacgcttaccggataggcaaaagcctccgccaaagcggtgcagg<br>acgcgacagacgagatcatgatagcctccacatgaattccagggccccgctcctccgtgcatcctggaa<br>acagatgcgccgcaatcaaaacctatgatagcggcgtgttattaactccaataatgatgttataaagctcg<br>aaagctgtcaatcggatgttttcgaaacaccggtgaaatagcacaatttcgcgccgaatctcgatcacgat<br>ctagagaaatccactacataattcgttatcaatcgcactattcacagcaccgtcttttctcgcaaagccagcc<br>acaaagatactcacaaacttctatattttcttttattttcaatgaattaaacatatttaaaaccgaagaaacatta<br>agctattgatggaaaacagtgggaaaaccgcactcgaaggcgcggagatttcccctacattcactctttgg<br>tgaaaaaaaatgcgccatctagaaaattttacagacaacgtgatagcgttatgttatctccagcatacgcc<br>atcgcccga	This work
pEryF-159-90	promoter	tatcaaacgccttcagcaattctgccgtattgtcgttatggaaacggatgatgcggctgaattagaccgccc<br>ggagattaccctcgaccattgtcatcacgcttaccggataggcaaaagcctccgccaaagcggtgcagg<br>acgcgacagacgagatcatgatagcctccacatgaattccagggccccgctcctccgtgcatcctggaa<br>acagatgcgccgcaatcaaaacctatgatagcggcgtgttattaactccaataatgatgttataaagctcg<br>aaagctgtcaatcggatgttttcgaaacaccggtgaaatagcacaatttcgcgccgaatctcgatcacgat<br>ctagagaaatccactacataattcgttatcaatcgcactattcacagcaccgtcttttctcgcaaagccagcc<br>acaaagatactcacaaacttctatattttcttttattttcaatgaattaaacatatttaaaaccgaagaaacatta<br>agctattgatggaaaacagtgggaaaaccgcactcgaaggcgcggagatttcccctacattcactctttgg<br>tgaaaaaaaatgcgccatctagaaaattttacagacaacgtgatagcgttatgttatctccagcatacgc	This work
pEryF-159-100	promoter	tatcaaacgccttcagcaattctgccgtattgtcgttatggaaacggatgatgcggctgaattagaccgccc<br>ggagattaccctcgaccattgtcatcacgcttaccggataggcaaaagcctccgccaaagcggtgcagg<br>acgcgacagacgagatcatgatagcctccacatgaattccagggccccgctcctccgtgcatcctggaa<br>acagatgcgccgcaatcaaaacctatgatagcggcgtgttattaactccaataatgatgttataaagctcg<br>aaagctgtcaatcggatgttttcgaaacaccggtgaaatagcacaatttcgcgccgaatctcgatcacgat<br>ctagagaaatccactacataattcgttatcaatcgcactattcacagcaccgtcttttctcgcaaagccagcc<br>acaaagatactcacaaacttctatattttcttttattttcaatgaattaaacatatttaaaaccgaagaaacatta<br>agctattgatggaaaacagtgggaaaaccgcactcgaaggcgcggagatttcccctacattcactctttgg<br>tgaaaaaaaatgcgccatctagaaaattttacagacaacgtgatagcgttatgttatctc	This work
pEryF-159-110	promoter	tatcaaacgccttcagcaattctgccgtattgtcgttatggaaacggatgatgcggctgaattagaccgccc<br>ggagattaccctcgaccattgtcatcacgcttaccggataggcaaaagcctccgccaaagcggtgcagg<br>acgcgacagacgagatcatgatagcctccacatgaattccagggccccgctcctccgtgcatcctggaa<br>acagatgcgccgcaatcaaaacctatgatagcggcgtgttattaactccaataatgatgttataaagctcg<br>aaagctgtcaatcggatgttttcgaaacaccggtgaaatagcacaatttcgcgccgaatctcgatcacgat<br>ctagagaaatccactacataattcgttatcaatcgcactattcacagcaccgtcttttctcgcaaagccagcc<br>acaaagatactcacaaacttctatattttcttttattttcaatgaattaaacatatttaaaaccgaagaaacatta<br>agctattgatggaaaacagtgggaaaaccgcactcgaaggcgcggagatttcccctacattcactctttgg<br>tgaaaaaaaatgcgccatctagaaaattttacagacaacgtgatagcgtt	This work
pEryF-159-120	promoter	tatcaaacgccttcagcaattctgccgtattgtcgttatggaaacggatgatgcggctgaattagaccgccc<br>ggagattaccctcgaccattgtcatcacgcttaccggataggcaaaagcctccgccaaagcggtgcagg<br>acgcgacagacgagatcatgatagcctccacatgaattccagggccccgctcctccgtgcatcctggaa<br>acagatgcgccgcaatcaaaacctatgatagcggcgtgttattaactccaataatgatgttataaagctcg<br>aaagctgtcaatcggatgttttcgaaacaccggtgaaatagcacaatttcgcgccgaatctcgatcacgat<br>ctagagaaatccactacataattcgttatcaatcgcactattcacagcaccgtcttttctcgcaaagccagcc<br>acaaagatactcacaaacttctatattttcttttattttcaatgaattaaacatatttaaaaccgaagaaacatta<br>agctattgatggaaaacagtgggaaaaccgcactcgaaggcgcggagatttcccctacattcactctttgg	This work

		tgaaaaaaaatgcgccatctagaaaattttacagacaacg	
pEryF-159-130	promoter	tatcaaacgccttcagcaattctgccgtattgtcgttatggaaacggatgatgcggctgaattagaccgcccggagattaccctcgaccattgtcatcacgcttaccggataggcaaaagcctccgccaaagcggtgcaggacgcgacagacgagatcatgatagcctccacatgaattccagggccccgctcctccgtgcatcctggaaacagatgcgccgcaatcaaaacctatgatagcggcgtgttattaactccaataatgatgttataaagctcgaaagctgtcaatcggatgttttcgaaacaccggtgaaatagcacaatttcgcgccgaatctcgatcacgatctagagaaatccactacataattcgttatcaatcgcactattcacagcaccgtcttttctcgcaaagccagccacaaagatactcacaaacttctatattttcttttattttcaatgaattaaacatatttaaaaccgaagaaacattaagctattgatggaaaacagtgggaaaaccgcactcgaaggcgcggagatttcccctacattcactctttggtgaaaaaaaatgcgccatctagaaaatttt	This work
pEryF-159-140	promoter	tatcaaacgccttcagcaattctgccgtattgtcgttatggaaacggatgatgcggctgaattagaccgcccggagattaccctcgaccattgtcatcacgcttaccggataggcaaaagcctccgccaaagcggtgcaggacgcgacagacgagatcatgatagcctccacatgaattccagggccccgctcctccgtgcatcctggaaacagatgcgccgcaatcaaaacctatgatagcggcgtgttattaactccaataatgatgttataaagctcgaaagctgtcaatcggatgttttcgaaacaccggtgaaatagcacaatttcgcgccgaatctcgatcacgatctagagaaatccactacataattcgttatcaatcgcactattcacagcaccgtcttttctcgcaaagccagccacaaagatactcacaaacttctatattttcttttattttcaatgaattaaacatatttaaaaccgaagaaacattaagctattgatggaaaacagtgggaaaaccgcactcgaaggcgcggagatttcccctacattcactctttggtgaaaaaaaatgcgccatct	This work
pEryF-159-150	promoter	tatcaaacgccttcagcaattctgccgtattgtcgttatggaaacggatgatgcggctgaattagaccgcccggagattaccctcgaccattgtcatcacgcttaccggataggcaaaagcctccgccaaagcggtgcaggacgcgacagacgagatcatgatagcctccacatgaattccagggccccgctcctccgtgcatcctggaaacagatgcgccgcaatcaaaacctatgatagcggcgtgttattaactccaataatgatgttataaagctcgaaagctgtcaatcggatgttttcgaaacaccggtgaaatagcacaatttcgcgccgaatctcgatcacgatctagagaaatccactacataattcgttatcaatcgcactattcacagcaccgtcttttctcgcaaagccagccacaaagatactcacaaacttctatattttcttttattttcaatgaattaaacatatttaaaaccgaagaaacattaagctattgatggaaaacagtgggaaaaccgcactcgaaggcgcggagatttcccctacattcactctttggtgaaaaaaaa	This work
pEryF-37	promoter	tatcaaacgccttcagcaattctgccgtattgtcgttatggaaacggatgatgcggctgaattagaccgcccggagattaccctcgaccattgtcatcacgcttaccggataggcaaaagcctccgccaaagcggtgcaggacgcgacagacgagatcatgatagcctccacatgaattccagggccccgctcctccgtgcatcctggaaacagatgcgccgcaatcaaaacctatgatagcggcgtgttattaactccaataatgatgttataaagctcgaaagctgtcaatcggatgttttcgaaacaccggtgaaatagcacaatttcgcgccgaatctcgatcacgatctagagaaatccactacataattcgttatcaatcgcactattcacagcaccgtcttttctcgcaaagccagccacaaagatactcacaaacttctatattttcttttattttcaatgaattaaacatatttaaaaccgaagaaacattaagctattgatggaaaacagtgggaaaaccgcactcgaaggcgcggagatttcccctacattcactctttggtgaaaaaaaatgcgccatctagaaaattttacagacaagctgatagcgttatgttatctc	This work
pEryF-35	promoter	tatcaaacgccttcagcaattctgccgtattgtcgttatggaaacggatgatgcggctgaattagaccgcccggagattaccctcgaccattgtcatcacgcttaccggataggcaaaagcctccgccaaagcggtgcaggacgcgacagacgagatcatgatagcctccacatgaattccagggccccgctcctccgtgcatcctggaaacagatgcgccgcaatcaaaacctatgatagcggcgtgttattaactccaataatgatgttataaagctcgaaagctgtcaatcggatgttttcgaaacaccggtgaaatagcacaatttcgcgccgaatctcgatcacgatctagagaaatccactacataattcgttatcaatcgcactattcacagcaccgtcttttctcgcaaagccagccacaaagatactcacaaacttctatattttcttttattttcaatgaattaaacatatttaaaaccgaagaaacattaagctattgatggaaaacagtgggaaaaccgcactcgaaggcgcggagatttcccctacattcactctttggtgaaaaaaaatgcgccatctagaaaattttacagacttgctgatagcgttatgttatctc	This work

pEryF-33	promoter	tatcaaacgccttcagcaattctgccgtattgtcgttatggaaacggatgatgcggctgaattagaccgccc<br>ggagattaccctcgaccattgtcatcacgcttaccggataggcaaaagcctccgccaaagcggtgcagg<br>acgcgacagacgagatcatgatagcctccacatgaattccagggccccgctcctccgtgcatcctggaa<br>acagatgcgccgcaatcaaaacctatgatagcggcgtgttattaactccaataatgatgttataaagctcg<br>aaagctgtcaatcggatgttttcgaaacaccggtgaaatagcacaatttcgcgccgaatctcgatcacgat<br>ctagagaaatccactacataattcgttatcaatcgcactattcacagcaccgtcttttctcgcaaagccagcc<br>acaaagatactcacaaacttctatattttcttttattttcaatgaattaaacatatttaaaaccgaagaaacatta<br>agctattgatggaaaacagtgggaaaaccgcactcgaaggcgcggagatttcccctacattcactctttgg<br>tgaaaaaaaatgcgccatctagaaaattttacagtgttgctgatagcgttatgttatctc	This work
pEryF-31	promoter	tatcaaacgccttcagcaattctgccgtattgtcgttatggaaacggatgatgcggctgaattagaccgccc<br>ggagattaccctcgaccattgtcatcacgcttaccggataggcaaaagcctccgccaaagcggtgcagg<br>acgcgacagacgagatcatgatagcctccacatgaattccagggccccgctcctccgtgcatcctggaa<br>acagatgcgccgcaatcaaaacctatgatagcggcgtgttattaactccaataatgatgttataaagctcg<br>aaagctgtcaatcggatgttttcgaaacaccggtgaaatagcacaatttcgcgccgaatctcgatcacgat<br>ctagagaaatccactacataattcgttatcaatcgcactattcacagcaccgtcttttctcgcaaagccagcc<br>acaaagatactcacaaacttctatattttcttttattttcaatgaattaaacatatttaaaaccgaagaaacatta<br>agctattgatggaaaacagtgggaaaaccgcactcgaaggcgcggagatttcccctacattcactctttgg<br>tgaaaaaaaatgcgccatctagaaaattttactctgttgctgatagcgttatgttatctc	This work
pEryF-29	promoter	tatcaaacgccttcagcaattctgccgtattgtcgttatggaaacggatgatgcggctgaattagaccgccc<br>ggagattaccctcgaccattgtcatcacgcttaccggataggcaaaagcctccgccaaagcggtgcagg<br>acgcgacagacgagatcatgatagcctccacatgaattccagggccccgctcctccgtgcatcctggaa<br>acagatgcgccgcaatcaaaacctatgatagcggcgtgttattaactccaataatgatgttataaagctcg<br>aaagctgtcaatcggatgttttcgaaacaccggtgaaatagcacaatttcgcgccgaatctcgatcacgat<br>ctagagaaatccactacataattcgttatcaatcgcactattcacagcaccgtcttttctcgcaaagccagcc<br>acaaagatactcacaaacttctatattttcttttattttcaatgaattaaacatatttaaaaccgaagaaacatta<br>agctattgatggaaaacagtgggaaaaccgcactcgaaggcgcggagatttcccctacattcactctttgg<br>tgaaaaaaaatgcgccatctagaaaatttttgtctgttgctgatagcgttatgttatctc	This work
pEryF-28	promoter	tatcaaacgccttcagcaattctgccgtattgtcgttatggaaacggatgatgcggctgaattagaccgccc<br>ggagattaccctcgaccattgtcatcacgcttaccggataggcaaaagcctccgccaaagcggtgcagg<br>acgcgacagacgagatcatgatagcctccacatgaattccagggccccgctcctccgtgcatcctggaa<br>acagatgcgccgcaatcaaaacctatgatagcggcgtgttattaactccaataatgatgttataaagctcg<br>aaagctgtcaatcggatgttttcgaaacaccggtgaaatagcacaatttcgcgccgaatctcgatcacgat<br>ctagagaaatccactacataattcgttatcaatcgcactattcacagcaccgtcttttctcgcaaagccagcc<br>acaaagatactcacaaacttctatattttcttttattttcaatgaattaaacatatttaaaaccgaagaaacatta<br>agctattgatggaaaacagtgggaaaaccgcactcgaaggcgcggagatttcccctacattcactctttgg<br>tgaaaaaaaatgcgccatctagaaaatttATGTCTGTTGCtgatagcgttatgttatctc	This work
pEryF-27	promoter	tatcaaacgccttcagcaattctgccgtattgtcgttatggaaacggatgatgcggctgaattagaccgccc<br>ggagattaccctcgaccattgtcatcacgcttaccggataggcaaaagcctccgccaaagcggtgcagg<br>acgcgacagacgagatcatgatagcctccacatgaattccagggccccgctcctccgtgcatcctggaa<br>acagatgcgccgcaatcaaaacctatgatagcggcgtgttattaactccaataatgatgttataaagctcg<br>aaagctgtcaatcggatgttttcgaaacaccggtgaaatagcacaatttcgcgccgaatctcgatcacgat<br>ctagagaaatccactacataattcgttatcaatcgcactattcacagcaccgtcttttctcgcaaagccagcc<br>acaaagatactcacaaacttctatattttcttttattttcaatgaattaaacatatttaaaaccgaagaaacatta<br>agctattgatggaaaacagtgggaaaaccgcactcgaaggcgcggagatttcccctacattcactctttgg<br>tgaaaaaaaatgcgccatctagaaaattaatgtctgttgctgatagcgttatgttatctc	This work
pEryF-25	promoter	tatcaaacgccttcagcaattctgccgtattgtcgttatggaaacggatgatgcggctgaattagaccgccc	This work

		ggagattaccctcgaccattgtcatcacgcttaccggataggcaaaagcctccgccaaagcggtgcagg acgcgacagacgagatcatgatagcctccacatgaattccagggccccgctcctccgtgcatcctggaa acagatgcgccgcaatcaaaacctatgatagcggcgtgttattaactccaataatgatgttataaagctcg aaagctgtcaatcggatgttttcgaaacaccggtgaaatagcacaatttcgcgccgaatctcgatcacgat ctagagaaatccactacataattcgttatcaatcgcactattcacagcaccgtcttttctcgcaaagccagcc acaaagatactcacaaacttctatattttcttttattttcaatgaattaaacatatttaaaaccgaagaaacatta agctattgatggaaaacagtgggaaaaccgcactcgaaggcgcggagatttcccctacattcactctttgg tgaaaaaaaatgcgccatctagaaaaaaaaatgtctgttgctgatagcgttatgttatctc	
pEryF-23	promoter	tatcaaacgccttcagcaattctgccgtattgtcgttatggaaacggatgatgcggctgaattagaccgccc ggagattaccctcgaccattgtcatcacgcttaccggataggcaaaagcctccgccaaagcggtgcagg acgcgacagacgagatcatgatagcctccacatgaattccagggccccgctcctccgtgcatcctggaa acagatgcgccgcaatcaaaacctatgatagcggcgtgttattaactccaataatgatgttataaagctcg aaagctgtcaatcggatgttttcgaaacaccggtgaaatagcacaatttcgcgccgaatctcgatcacgat ctagagaaatccactacataattcgttatcaatcgcactattcacagcaccgtcttttctcgcaaagccagcc acaaagatactcacaaacttctatattttcttttattttcaatgaattaaacatatttaaaaccgaagaaacatta agctattgatggaaaacagtgggaaaaccgcactcgaaggcgcggagatttcccctacattcactctttgg tgaaaaaaaatgcgccatctagaattaaaatgtctgttgctgatagcgttatgttatctc	This work
pEryF-21	promoter	tatcaaacgccttcagcaattctgccgtattgtcgttatggaaacggatgatgcggctgaattagaccgccc ggagattaccctcgaccattgtcatcacgcttaccggataggcaaaagcctccgccaaagcggtgcagg acgcgacagacgagatcatgatagcctccacatgaattccagggccccgctcctccgtgcatcctggaa acagatgcgccgcaatcaaaacctatgatagcggcgtgttattaactccaataatgatgttataaagctcg aaagctgtcaatcggatgttttcgaaacaccggtgaaatagcacaatttcgcgccgaatctcgatcacgat ctagagaaatccactacataattcgttatcaatcgcactattcacagcaccgtcttttctcgcaaagccagcc acaaagatactcacaaacttctatattttcttttattttcaatgaattaaacatatttaaaaccgaagaaacatta agctattgatggaaaacagtgggaaaaccgcactcgaaggcgcggagatttcccctacattcactctttgg tgaaaaaaaatgcgccatctagttttaaaatgtctgttgctgatagcgttatgttatctc	This work
pEryR-732	promoter	ggcggctatgtgctttcattgacatttggattcctcccgcgaccgaaaatacgtcgctcgcagccctgtttata atgaaatcgggcgatggcgtatgctggagataacataacgctatcacgttgtctgtaaaattttctagatggc gcattttttttcaccaaagagtgaatgtaggggaaatctccgcgccttcgagtgcggttttcccactgttttccat caatagcttaatgtttcttcggttttaaatatgtttaattcattgaaaataaaagaaaatatagaagtttgtgagt atctttgtggctggctttgcgagaaaagacggtgctgtgaatagtgcgattgataacgaattatgtagtggatt tctctagatcgtgatcgagattcggcgcgaaattgtgctatttcaccggtgtttcgaaaacatccgattgaca gctttcgagctttataacatcattattggagttaataacacgccgctatcataggttttgattgcggcgcatctgt ttccaggatgcacggaggagcggggccctggaattcatgtggaggctatcatgatctcgtctgtcgcgtcct gcaccgctttggcggaggcttttgcctatccggtaagcgtgatgacaatggtcgagggtaatctccgggcg gtctaattcagccgcatcatccgtttccataacgacaatacggcagaattgctgaaggcgtttgata	This work
pEryR-632	promoter	gagataacataacgctatcacgttgtctgtaaaattttctagatggcgcattttttttcaccaaagagtgaatgt aggggaaatctccgcgccttcgagtgcggttttcccactgttttccatcaatagcttaatgtttcttcggttttaaa tatgtttaattcattgaaaataaaagaaaatatagaagtttgtgagtatctttgtggctggctttgcgagaaaa gacggtgctgtgaatagtgcgattgataacgaattatgtagtggatttctctagatcgtgatcgagattcggc gcgaaattgtgctatttcaccggtgtttcgaaaacatccgattgacagctttcgagctttataacatcattattg gagttaataacacgccgctatcataggttttgattgcggcgcatctgtttccaggatgcacggaggagcgg ggccctggaattcatgtggaggctatcatgatctcgtctgtcgcgtcctgcaccgctttggcggaggcttttgc ctatccggtaagcgtgatgacaatggtcgagggtaatctccgggcggtctaattcagccgcatcatccgttt ccataacgacaatacggcagaattgctgaaggcgtttgata	This work
pEryR-532	promoter	gcggttttcccactgttttccatcaatagcttaatgtttcttcggttttaaatatgtttaattcattgaaaataaaaga	This work

		aaatatagaagtttgtgagtatctttgtggctggctttgcgagaaaagacggtgctgtgaatagtgcgattgataacgaattatgtagtggatttctctagatcgtgatcgagattcggcgcgaaattgtgctatttcaccggtgtttcgaaaacatccgattgacagctttcgagctttataacatcattattggagttaataacacgccgctatcataggttttgattgcggcgcatctgtttccaggatgcacggaggagcggggccctggaattcatgtggaggctatcatgatctcgtctgtcgcgtcctgcaccgctttggcggaggcttttgcctatccggtaagcgtgatgacaatggtcgagggtaatctccgggcggtctaattcagccgcatcatccgtttccataacgacaatacggcagaattgctgaaggcgtttgata	
pEryR-432	promoter	tctttgtggctggctttgcgagaaaagacggtgctgtgaatagtgcgattgataacgaattatgtagtggatttctctagatcgtgatcgagattcggcgcgaaattgtgctatttcaccggtgtttcgaaaacatccgattgacagctttcgagctttataacatcattattggagttaataacacgccgctatcataggttttgattgcggcgcatctgtttccaggatgcacggaggagcggggccctggaattcatgtggaggctatcatgatctcgtctgtcgcgtcctgcaccgctttggcggaggcttttgcctatccggtaagcgtgatgacaatggtcgagggtaatctccgggcggtctaattcagccgcatcatccgtttccataacgacaatacggcagaattgctgaaggcgtttgata	This work
pEryR-332	promoter	cgaaattgtgctatttcaccggtgtttcgaaaacatccgattgacagctttcgagctttataacatcattattggagttaataacacgccgctatcataggttttgattgcggcgcatctgtttccaggatgcacggaggagcggggccctggaattcatgtggaggctatcatgatctcgtctgtcgcgtcctgcaccgctttggcggaggcttttgcctatccggtaagcgtgatgacaatggtcgagggtaatctccgggcggtctaattcagccgcatcatccgtttccataacgacaatacggcagaattgctgaaggcgtttgata	This work
pEryR-322	promoter	ctatttcaccggtgtttcgaaaacatccgattgacagctttcgagctttataacatcattattggagttaataacacgccgctatcataggttttgattgcggcgcatctgtttccaggatgcacggaggagcggggccctggaattcatgtggaggctatcatgatctcgtctgtcgcgtcctgcaccgctttggcggaggcttttgcctatccggtaagcgtgatgacaatggtcgagggtaatctccgggcggtctaattcagccgcatcatccgtttccataacgacaatacggcagaattgctgaaggcgtttgata	This work
pEryR-312	promoter	ggtgtttcgaaaacatccgattgacagctttcgagctttataacatcattattggagttaataacacgccgctatcataggttttgattgcggcgcatctgtttccaggatgcacggaggagcggggccctggaattcatgtggaggctatcatgatctcgtctgtcgcgtcctgcaccgctttggcggaggcttttgcctatccggtaagcgtgatgacaatggtcgagggtaatctccgggcggtctaattcagccgcatcatccgtttccataacgacaatacggcagaattgctgaaggcgtttgata	This work
pEryR-302	promoter	aaacatccgattgacagctttcgagctttataacatcattattggagttaataacacgccgctatcataggttttgattgcggcgcatctgtttccaggatgcacggaggagcggggccctggaattcatgtggaggctatcatgatctcgtctgtcgcgtcctgcaccgctttggcggaggcttttgcctatccggtaagcgtgatgacaatggtcgagggtaatctccgggcggtctaattcagccgcatcatccgtttccataacgacaatacggcagaattgctgaaggcgtttgata	This work
pEryR-292	promoter	ttgacagctttcgagctttataacatcattattggagttaataacacgccgctatcataggttttgattgcggcgcatctgtttccaggatgcacggaggagcggggccctggaattcatgtggaggctatcatgatctcgtctgtcgcgtcctgcaccgctttggcggaggcttttgcctatccggtaagcgtgatgacaatggtcgagggtaatctccgggcggtctaattcagccgcatcatccgtttccataacgacaatacggcagaattgctgaaggcgtttgata	This work
pEryR-282	promoter	tcgagctttataacatcattattggagttaataacacgccgctatcataggttttgattgcggcgcatctgtttccaggatgcacggaggagcggggccctggaattcatgtggaggctatcatgatctcgtctgtcgcgtcctgcaccgctttggcggaggcttttgcctatccggtaagcgtgatgacaatggtcgagggtaatctccgggcggtctaattcagccgcatcatccgtttccataacgacaatacggcagaattgctgaaggcgtttgata	This work
pEryR-272	promoter	taacatcattattggagttaataacacgccgctatcataggttttgattgcggcgcatctgtttccaggatgcacggaggagcggggccctggaattcatgtggaggctatcatgatctcgtctgtcgcgtcctgcaccgctttggcggaggcttttgcctatccggtaagcgtgatgacaatggtcgagggtaatctccgggcggtctaattcagccgcatcatccgtttccataacgacaatacggcagaattgctgaaggcgtttgata	This work

pEryR-262	promoter	attggagttaataacacgccgctatcataggttttgattgcggcgcatctgtttccaggatgcacggaggagcggggccctggaattcatgtggaggctatcatgatctcgtctgtcgcgtcctgcaccgctttggcggaggcttttgcctatccggtaagcgtgatgacaatggtcgagggtaatctccgggcggtctaattcagccgcatcatccgtttccataacgacaatacggcagaattgctgaaggcgtttgata	This work
pEryR-252	promoter	ataacacgccgctatcataggttttgattgcggcgcatctgtttccaggatgcacggaggagcggggccctggaattcatgtggaggctatcatgatctcgtctgtcgcgtcctgcaccgctttggcggaggcttttgcctatccggtaagcgtgatgacaatggtcgagggtaatctccgggcggtctaattcagccgcatcatccgtttccataacgacaatacggcagaattgctgaaggcgtttgata	This work
pEryR-242	promoter	gctatcataggttttgattgcggcgcatctgtttccaggatgcacggaggagcggggccctggaattcatgtggaggctatcatgatctcgtctgtcgcgtcctgcaccgctttggcggaggcttttgcctatccggtaagcgtgatgacaatggtcgagggtaatctccgggcggtctaattcagccgcatcatccgtttccataacgacaatacggcagaattgctgaaggcgtttgata	This work
pEryR-232	promoter	gttttgattgcggcgcatctgtttccaggatgcacggaggagcggggccctggaattcatgtggaggctatcatgatctcgtctgtcgcgtcctgcaccgctttggcggaggcttttgcctatccggtaagcgtgatgacaatggtcgagggtaatctccgggcggtctaattcagccgcatcatccgtttccataacgacaatacggcagaattgctgaaggcgtttgata	This work
pEryR-132	promoter	ctttggcggaggcttttgcctatccggtaagcgtgatgacaatggtcgagggtaatctccgggcggtctaattcagccgcatcatccgtttccataacgacaatacggcagaattgctgaaggcgtttgata	This work
pEryR-32	promoter	caatacggcagaattgctgaaggcgtttgata	This work
pEry2-400	promoter	acgccaatggccagcgccttgaaaccgcgctgaccgcccgcaccatcgctgcatcagtcgaaaacgcggatatgagccgaatcgtgggccttgccggtggaatttccaaggcggaagccatccgcgctgtgctgaaaagcggtcgtctttacggtctcatcaccgatgaacgcacggcaaagacgctcgttgagcagccgaacggaaaataacataataacatttttgcaatggattattttgtgattttgtgatctaacttcgcgcagaaaatgttccgccaataagatcggacgagagcgtgaagcgcgacgacagaagacaggcgattataaaccttctgatcgaaaatcaggccgtcgacctcgacgatctggcggatcggtttgccgtatcaaaa	This work
pEry2-300	promoter	aatttccaaggcggaagccatccgcgctgtgctgaaaagcggtcgtctttacggtctcatcaccgatgaacgcacggcaaagacgctcgttgagcagccgaacggaaaataacataataacatttttgcaatggattattttgtgattttgtgatctaacttcgcgcagaaaatgttccgccaataagatcggacgagagcgtgaagcgcgacgacagaagacaggcgattataaaccttctgatcgaaaatcaggccgtcgacctcgacgatctggcggatcggtttgccgtatcaaaa	This work
pEry2-200	promoter	aacggaaaataacataataacatttttgcaatggattattttgtgattttgtgatctaacttcgcgcagaaaatgttccgccaataagatcggacgagagcgtgaagcgcgacgacagaagacaggcgattataaaccttctgatcgaaaatcaggccgtcgacctcgacgatctggcggatcggtttgccgtatcaaaa	This work
pEry2-100	promoter	cgtgaagcgcgacgacagaagacaggcgattataaaccttctgatcgaaaatcaggccgtcgacctcgacgatctggcggatcggtttgccgtatcaaaa	This work
B0034	RBS	aaagaggagaaa	1, BBa_B0034
eryA-RBS	RBS	ggaatccaaatgtcaatgaaagcacatagccgcc	This work
eryB-RBS	RBS	agactatgggatgcaattgcatccgaaggagcatgaa	This work
eryC-RBS	RBS	ggaatggttcgacaacgctaaaatggtctcttgaggacact	This work
eryD-RBS	RBS	gaagatataaacgatttcaggaaccggaccgctttca	This work
eryE-RBS	RBS	aacgacaatacggcagaattgctgaaggcgtttgata	This work
eryF-RBS	RBS	gaagaactggctcgtcacgcctgtggggctcgaagtgaa	This work
eryG-RBS	RBS	aaaaggatagagcataagacccgacaaagtcgggttgttaa	This work
eryH-RBS	RBS	gctgacgactgcgcataaacctagaggacgaaaatcgagc	This work
eryI-RBS	RBS	ggccaagtgtgccgccaagactcgagctaattaaggagcaat	This work

pR-eryA	promoter-RBS	tatcaaacgccttcagcaattctgccgtattgtcgttatggaaacggatgatgcggctgaattagaccgcccggagattaccctcgaccattgtcatcacgcttaccggataggcaaaagcctccgccaaagcggtgcaggacgcgacagacgagatcatgatagcctccacatgaattccagggccccgctcctccgtgcatcctggaaacagatgcgccgcaatcaaaacctatgatagcggcgtgttattaactccaataatgatgttataaagctcgaaagctgtcaatcggatgttttcgaaacaccggtgaaatagcacaatttcgcgccgaatctcgatcacgatctagagaaatccactacataattcgttatcaatcgcactattcacagcaccgtcttttctcgcaaagccagccacaaagatactcacaaacttctatattttcttttattttcaatgaattaaacatatttaaaaccgaagaaacattaagctattgatggaaaacagtgggaaaaccgcactcgaaggcgcggagatttcccctacattcactctttggtgaaaaaaaatgcgccatctagaaaattttacagacaacgtgatagcgttatgttatctccagcatacgccatcgcccgatttcattataaacagggctgcgagcgacgtattttcggtcgcgggaggaatccaaatgtcaatgaaagcacatagccgcc	This work
pR-eryB	promoter-RBS	cgtgagaaaggtgacatcatcatcgggatcgacgcgggcacgtctgtgctcaaggcagtcgctttcgaccttagcggacgccagatcgaatctgctgccgttcgcaataaatacgataccggcgaacatggcgctgtcacacagtcgctttcccagacgtggcaggactgcgcgcgcgccttgcgcgacctcggtgcaaagatgcccggccttgccgaacgcactgcggcaatcgccgttaccggtcagggtgacggaacctggcttgttggccgcgacaaccagccggtcggcgatgcctggctgtggctcgatgcccgcgcggcaacgacggtgacgcgcctcgccgctggccccgtgaaccgcgcgcgctttgaagcgaccggcactgggttgaacacctgccagcaaggctcgcagatggcgcatatggacagcatcgcgcccgagctgctggacaatgccgaagtcgcgctccactgcaaagactggctctatctcaatctcaccggcgttcgcgccaccgacccttcggaagcaagcttcaccttcggcaatttccgcaatcgccagtatgacgatgtcgtcatcgacgcgctcggtctgaggaaacggcgtggattgctgccggaaatcatcgacggcaccgaagtgcagcatccgctatcccccgaagcggcagctgcaacgggtttgcgggcagggacgccggtgggtctcgcctatgtcgatatggccatgacagcacttggtgcgggcgtgcgcggcggcacagcaggcgccgggtgctcgaccattggttcgacaggcgtccatatgcgcgccaagccggttgccgatatccacctgaacaaggaaggcaccggttacgtcatcgcgctgcccattcccggcatcgtcacccaggtgcagaccaatatgggggcaacgatcaacatcgactggatattgcaggttgctgccgatctcatgtcgacgccagacaagcctgtttcgctcagtgacctcattccccgccttgatgactggtttaatgccagccgtccgggtgcaatcctctatcatccgtatatttcggaagcgggcgaacgcggtcccttcgtcaatgcctatgcccgggccggtttcgtcggtctttccagccgcgaccgtttcccggaaatggtgcgctcggttgtcgagggcctcggaatggcgacgcgcgattgctacacggccatgggtgaaatgcccgcagaattgcgcatcaccggcggtgcagcgcgttcaaaagcattgcgcagcacccttgccgcagcggtgaatgcgcccgtgcgcgtttcctcgcgtgaagaggcgggagccgcaggtgctgccatgatggctgcggtggccattggcgcttatccaagcatggatgcatgtattgcggaatgggtcgagccgctgcttggcccttgcgaggccccggatggtccgcgcgcaaagcactatgaagaactcttcgttgcctatcaggaagcccggctggcactgacgcccgtctgggacaagttggcttccgatagagcatttccagcaaaagcgtgaagcggttttgcgttcggaaatgcggtgaacaaaacgaataaggccggaccttccttccggcacatatagactatgggatgcaattgcatccgaaggagcatgaa	This work
pR-eryC	promoter-RBS	gctgaaccggaaacctgcgatctctttgtcatcggcggtggtatcaacggcgcgggcgtggcccgtgacgccgccgggcgcggcctcaaggtggtgctggcggaaaaagacgatctggcgcagggaacttcgtcacgctccggcaagctggtacatggtggtctgcgttatctcgaatattatgagtttcgccttgtgcgcgaagcgctgatcgagcgcgaagtgctgttgaacgccgctccccatatcatctggccaatgcgtttcgttctgccgcacagcccgcaggaccgtcccgcctggctggtgcggcttggcctgtttctctatgaccacctcggcggccgcaagaagcttcccggcacacgcacgctcgacctgcggcgcgatccggaaggcacgccgatcctcgaccaatataccaagggcttcgaatattccgactgttgggtcgatgatgcccgtctcgttgcattgaatgcggttggcgctgctgaaaaaggcgcgaccattctgacccgcacgcctgtcgtctccgctcgtcgcgaaaaaggcggctggatcgtggaaacgaaaaaccgcgacacgggcgaaacccgcacattccgcgcgcgctgcatcgtcaattgcgccggaccatgggtcacggatgtcatccacaatgtcgccggctccaactcgtcgcgcaatgtgcgtctcgtcaagggcagccacatcatcgttccgaaattctggtcgggcgcaaatgcctatctcgtccagaaccacgaca	This work

		agcgcgttatctttatcaatccctacgagggcgacaaggcgctgatcggcaccaccgacatcgcctatgaaggccgtgccgaagatgttgcagcagatgagaaggaaatcgaatatctgctgactgcagtgaaccgctatttcaaggaaaagctcaggcgtgaagacgtgctgcattccttctccggtgtgcgtccgctgttcgacgacggcaagggcaacccttccgccgtcacccgcgactatgtcttcgacctcgatgaaaccaacgacgcgccgctgctcaacgtctttggcggcaagatcaccaccttccgcgagctggccgagcgcggcatgcatcgcctcaagcacattttcccgaagatgggcggcgactggactcatggcgcaccgcttccgggcggcgaaatcgccaatgccgattatgaaaccttcgccaactccttgcgcgacacctatccgtggatgccgcgcgcgctcgtccaacactacggtcgtctctacggcgcacgcacgaaagacgttgttgcaggcgcacagaacctcgaagggctcggccgtcatttcggcggcaatttccatgaggcggaagtgcgctatctggtggccagggaatgggcaatgacggcggaagacattctctatcgccgcaccaagcactatctgcacctgaccgaagccgaacgcgccgctttcgtggaatggttcgacaacgctaaaatggtctcttgaggacact	
pR-eryD	promoter-RBS	gcccttacgctttctctgaacaccaatccacttgtgaaccgctttgccgaaccggatgacctgattgaaacggttgcgcgcgatctgcgcctgcgcgatctccagctcacacacgagttcatcaatccaagctggcaggctccaaccatccgccgcctgacgcgcgacatggacaaggccttgcagcgtaccggtgttcgcgtcacttcgggcatgaccggcccctatggccgcctcaaccatttcggccatccggacgcagaagtgcgccgttactatgtcgactggttcaagaccttcgccgacattatcggcgatcttggcggcaagtcggtcggcacgcagtttgcgatcttcacctataaggatttcgatgacgctgcgcgccgcgaagatctgatcaagatcgccatcgattgctgggcggaagtcgccgaacatgcgagccgtgccggtctcgactatgtgttctgggaaccgatgagcatcgggcgtgaatttggcgagacgattgccgaatgcatgaagcttcaggatcgcctgaccgccgccgacatggctattccgatgtggatgatggccgatatcgaccacggcgatgtgacttccgccaatccggacgatttcgatccctacgcctgggcacgcgccgtgccgaaagtctcgccgatcattcacatcaagcagagcctgatggacaagggcggacatcgtcctttcacagccgcgttcaatgccaagggacgcatccagccggaaccgcttctgaaagcctttgccgaaggcggcgctgtggacaatgaaatctgcctcgaactgtcgttcaaggagcgcgagccgaatgaccgtgaagtcattccgcagattgcggaaagtgtggctttctgggctccgcacattgacaccggcgctaaggacttgaagatataaacgatttcaggaaccggaccgctttca	This work
pR-eryE	promoter-RBS	ggcggctatgtgctttcattgacatttggattcctcccgcgaccgaaaatacgtcgctcgcagccctgtttataatgaaatcgggcgatggcgtatgctggagataacataacgctatcacgttgtctgtaaaattttctagatggcgcattttttttcaccaaagagtgaatgtaggggaaatctccgcgccttcgagtgcggttttcccactgttttccatcaatagcttaatgtttcttcggttttaaatatgtttaattcattgaaaataaaagaaaatatagaagtttgtgagtatctttgtggctggctttgcgagaaaagacggtgctgtgaatagtgcgattgataacgaattatgtagtggatttctctagatcgtgatcgagattcggcgcgaaattgtgctatttcaccggtgtttcgaaaacatccgattgacagctttcgagctttataacatcattattggagttaataacacgccgctatcataggttttgattgcggcgcatctgtttccaggatgcacggaggagcggggccctggaattcatgtggaggctatcatgatctcgtctgtcgcgtcctgcaccgctttggcggaggcttttgcctatccggtaagcgtgatgacaatggtcgagggtaatctccgggcggtctaattcagccgcatcatccgtttccataacgacaatacggcagaattgctgaaggcgtttgata	This work
pR-eryF	promoter-RBS	agtgcacaaaccagtcaagtttcacaaaaacccgccaagctgagccgcgccgtcgtctggagcgtgatcgctgcgatcgtggttgtcggtgcgattgccttcgacacgaaggtcgtgaagatcgggtctgattccgatgtgcgccagcaggctttctcccccgatgcctacggtgcttccgaattccccaaggtgaaggcgagcgttgagcagcgcgccgtggatgcagtggaagttggaacggctctcgccgccgacaaggccgccgccggaaagaaatatggcgtcggcgacgtcaatccggttgtcccggtcaagtttacaggcaccgttgaggagcgcaaatccaactacaatgtcgtgaaggtcgacggcctgccggaaggcgttgccatccgcgttcagaccggccctgccgtcaacggcaccgatctgcgcgatgcgaccggtgaaatccagttcggccagttcaagaaccagatcgaatatcaaaatgccggttctgccctgaacaacgagatgaagaagcaagtgctttccggcgtcgatgtcgagaatctcgtcggcaagaccgtgacggtggttggcgtgttcaaggtcgtcaatccgaagaactggctcgtcacgcctgtggggctcgaagtgaa	This work
pR-eryG	promoter-RBS	agcaccgcttcgaaaatcgaaggcaaaaacggtgatgtcgttcttgccgcaaacaacgttgccaaatcct	This work

		atggtcgcattcatgccctgaagggcgtgaatttcgagattcgccgtggtcaggtcacgacgcttttcggcgaaaacggcgctggcaaatcaacgttgatgaaagtgctttcgggcgtcatccagccgacttcggggacgatcattctcgatggcgaacctgtgacgttcaattcgtccaccgaagcgcgcgacctcggcatctcgatcatccatcaggaattgagcctcgcgcccaacatgaacgtgcgcgacaacatcttcatggggcgcgaaatccgcaccgccaccggcgttgattttgccgaggaagaacgcctgacgcgcgcgcttctgaaggaactggaagaagacatcgacccgctgacgccggtcgaggaacttcgtctcggccagcagcaggttgttgaaatcgcccgcgccctttcggtcaattcgcgcattctcatcatggatgagccgacttcggcgctcagcgcctcggaagtggaagtcctgttcaaggtcattcgcgacctgacggcgcgcggtgtcgccatcgtctacatttcgcatcatctggaagaagcgttgcagatcaccaatcatgcggtggtgctgcgcgacggaaccatgacggcctatgcgccgcgtgaggaaattgatctggaatggatcgtgcgcaacatggtcggcgagaacttcgatctcggctcgcctccaaccggatatgactggggcgatgtggcgctctctgttgagaacctgaccgttcccgacccgggtggcgcgggcttctcgctggtcgaccgcatgtcgctcaatgtacgcgccggtgaaatcgtctgcatttatggtcttatgggcgccggtcgcacagaactgctggaaaccgttgccggtcgtctcaaggcaagcggcggacgtgttctcctcaaagggcaggacgtttccggcctcaccatcgcacaacgtatcgagaagggtcttgtgctggtgccggaagatcgccagcgcgacggtctcgtccagacgatgacggtcggcaagaacctgtcgctcgccagcatcgccgaaatgaccaagggtctgttcacatcgcgcaagcgtgaaaagcagattgtcgatcagtcgatcaagaatgtgcacatcaagacggatggcggcgaagcagcaatcggttcgctttccggtggtaaccagcagaaggtcgtgatcggcaagatgctggcgaccgaaccggaagtcatcctgcttgatgagccgagccgcgggatcgacatcggggcgaaggcggaagtgttcaagcttctggctgaaaaggcgaagcagggtctggctgtcgtctacacgacttcggaagtcggcgaatgcctcagcatcgctcatcgtatcatcgtcatgcaccgtggacgcatctctgccgaattcggatcggacgtgtccaaggagaagatcatggccgcctcgggcgaagccatggtcggtcactaaaacataatcccgaaaagttgcagacttttcggacaagattatgcgttaaaaacaaaaggatagagcataagacccgacaaagtcgggttgttaa	
pR-eryH	promoter-RBS	acgatccatcgcgatctggattcgctggaacataccggcgtgctgcgcaaagtacgcggcggcgcgacaatcgataccggcacacaatttgaaagcgatttccgcttccgcgaacggcaggacggcgaagcgaagcagaaaatggcccgcgccgcgcttgagcttgttgaacccggcatgacggtgatgattaatgacggctccatggcggctgtccttggcggcatgcttctggaaaagcgcccgctgacagtcatcaccaacaatgccgtcatcatcgatgaactgaagggcgagaacgggatcaatctgattgcgctcggcggaacctattcagccaagttcaatgcgtttttcggcatcctgacggaaggggccctgtcgcatctgagcgccgacatcgccttcatttcctcgcctgcagtcaatggcaagctcgtctatcacatggatgagaatgttgttcgcaccaagcgcgcgatgacggcatccgccgcccgcacctgccttctggtcaatcaccagcggttcggacgccctgccctgcatgtcatggctgatctttccgatttcgacgcgatcataaccgatgcctcccccgacgcgaccgtactggccgatcttgaacaggcgggcattgcactgaccatcgctgacgactgcgcataaacctagaggacgaaaatcgagc	This work
pR-eryI	promoter-RBS	acaaaattctggattggcaccagctggaagatgaacaagacgctggccgaggcccgcattttcgccgaagccttgaaagctgccgatgcaagccgctcgaccgacattcagcgtttcgtcatcccgccctttaccgccgtgcgcgaagtgaaggaaatcctgttcggcacctccgtcaaggtcggcgcgcagaacatgcattgggccgatcagggagcatggaccggcgagatttcgccgctgatgctcaaggactgcaatctcgatatcgtcgaactcggtcattccgagcgccgtgaacatttcggtgaaaccaacgaaacggtcggcctcaaggtcgaagctgcggtgcgccacggcctgatcccactcatctgcatcggtgagacgctggaagaccgcgaaagcggacgcgccgcggaagttcttgaggaagaagtgcgcggtgcactttccaagctttccggtgaccagaagcaggcggaaatcctgtttgcctatgagccggtctgggccatcggcgaaaatggtatcccggcatcggcggaatatgccgatgcgcgccaggcggaaatcatcgcggttgcagaaagcgtactcggtcgtcgtgtgccttgcctctatggcggctcggtcaatccgggcaattgcgaagagctgatcgcttgcccgcacattgacgggcttttcatcggtcgctcggcatggaacgtcgaaggttatctcgacattctggccaagtgtgccgccaagactcgagctaattaaggagcaat	This work
B0015	terminator	ccaggcatcaaataaaacgaaaggctcagtcgaaagactgggcctttcgttttatctgttgtttgtcggtgaa	1, BBa_B0015

		cgctctctactagagtcacactggctcaccttcgggtgggcctttctgcgtttata	
His operon terminator	terminator	tccggcaaaaaagggcaaggtgtcaccaccctgcccttttttctttaaaaccgaaaaga	1, pSB1C3
Bacterial terminator	terminator	gaaatcatccttagcgaaagctaaggattttttttatctgaaat	1, pSB1C3
eryA	CDS	atgcgtgagaaaggtgacatcatcatcgggatcgacgcgggcacgtctgtgctcaaggcagtcgctttcg<br>accttagcggacgccagatcgaatctgctgccgttcgcaataaatacgataccggcgaacatggcgctgt<br>cacacagtcgctttcccagacgtggcaggactgcgcgcgcgccttgcgcgacctcggtgcaaagatgcc<br>cggccttgccgaacgcactgcggcaatcgccgttaccggtcagggtgacggaacctggcttgttggccgc<br>gacaaccagccggtcggcgatgcctggctgtggctcgatgcccgcgcggcaacgacggtgacgcgcct<br>cgccgctggccccgtgaaccgcgcgcgctttgaagcgaccggcactgggttgaacacctgccagcaag<br>gctcgcagatggcgcatatggacagcatcgcgcccgagctgctggacaatgccgaagtcgcgctccact<br>gcaaagactggctctatctcaatctcaccggcgttcgcgccaccgacccttcggaagcaagcttcaccttc<br>ggcaatttccgcaatcgccagtatgacgatgtcgtcatcgacgcgctcggtctgaggaaacggcgtggatt<br>gctgccggaaatcatcgacggcaccgaagtgcagcatccgctatcccccgaagcggcagctgcaacg<br>ggtttgcgggcagggacgccggtgggtctcgcctatgtcgatatggccatgacagcacttggtgcgggcgt<br>gcgcggcggcacagcaggcgccgggtgctcgaccattggttcgacaggcgtccatatgcgcgccaagc<br>cggttgccgatatccacctgaacaaggaaggcaccggttacgtcatcgcgctgcccattcccggcatcgt<br>cacccaggtgcagaccaatatgggggcaacgatcaacatcgactggatattgcaggttgctgccgatctc<br>atgtcgacgccagacaagcctgtttcgctcagtgacctcattccccgccttgatgactggtttaatgccagcc<br>gtccgggtgcaatcctctatcatccgtatatttcggaagcgggcgaacgcggtcccttcgtcaatgcctatgc<br>ccgggccggtttcgtcggtctttccagccgcgaccgtttcccggaaatggtgcgctcggttgtcgagggcct<br>cggaatggcgacgcgcgattgctacacggccatgggtgaaatgcccgcagaattgcgcatcaccggcg<br>gtgcagcgcgttcaaaagcattgcgcagcacccttgccgcagcggtgaatgcgcccgtgcgcgtttcctc<br>gcgtgaagaggcgggagccgcaggtgctgccatgatggctgcggtggccattggcgcttatccaagcat<br>ggatgcatgtattgcggaatgggtcgagccgctgcttggcccttgcgaggccccggatggtccgcgcgca<br>aagcactatgaagaactcttcgttgcctatcaggaagcccggctggcactgacgcccgtctgggacaagt<br>tggcttccgatagagcatttccagcaaaagcgtga	This work
eryB	CDS	atggctgaaccggaaacctgcgatctctttgtcatcggcggtggtatcaacggcgcgggcgtggcccgtg<br>acgccgccgggcgcggcctcaaggtggtgctggcggaaaaagacgatctggcgcagggaacttcgtc<br>acgctccggcaagctggtacatggtggtctgcgttatctcgaatattatgagtttcgccttgtgcgcgaagcg<br>ctgatcgagcgcgaagtgctgttgaacgccgctccccatatcatctggccaatgcgtttcgttctgccgcac<br>agcccgcaggaccgtcccgcctggctggtgcggcttggcctgtttctctatgaccacctcggcggccgcaa<br>gaagcttcccggcacacgcacgctcgacctgcggcgcgatccggaaggcacgccgatcctcgaccaat<br>ataccaagggcttcgaatattccgactgttgggtcgatgatgcccgtctcgttgcattgaatgcggttggcgct<br>gctgaaaaaggcgcgaccattctgacccgcacgcctgtcgtctccgctcgtcgcgaaaaaggcggctgg<br>atcgtggaaacgaaaaaccgcgacacgggcgaaacccgcacattccgcgcgcgctgcatcgtcaattg<br>cgccggaccatgggtcacggatgtcatccacaatgtcgccggctccaactcgtcgcgcaatgtgcgtctc<br>gtcaagggcagccacatcatcgttccgaaattctggtcgggcgcaaatgcctatctcgtccagaaccacg<br>acaagcgcgttatctttatcaatccctacgagggcgacaaggcgctgatcggcaccaccgacatcgccta<br>tgaaggccgtgccgaagatgttgcagcagatgagaaggaaatcgaatatctgctgactgcagtgaaccg<br>ctatttcaaggaaaagctcaggcgtgaagacgtgctgcattccttctccggtgtgcgtccgctgttcgacga<br>cggcaagggcaacccttccgccgtcacccgcgactatgtcttcgacctcgatgaaaccaacgacgcgcc<br>gctgctcaacgtctttggcggcaagatcaccaccttccgcgagctggccgagcgcggcatgcatcgcctc<br>aagcacattttcccgaagatgggcggcgactggactcatggcgcaccgcttccgggcggcgaaatcgcc<br>aatgccgattatgaaaccttcgccaactccttgcgcgacacctatccgtggatgccgcgcgcgctcgtcca	This work

		acactacggtcgtctctacggcgcacgcacgaaagacgttgttgcaggcgcacagaacctcgaagggc tcggccgtcatttcggcggcaatttccatgaggcggaagtgcgctatctggtggccagggaatgggcaat gacggcggaagacattctctatcgccgcaccaagcactatctgcacctgaccgaagccgaacgcgccg ctttcgtggaatggttcgacaacgctaaaatggtctcttga	
eryC	CDS	atggcccttacgctttctctgaacaccaatccacttgtgaaccgctttgccgaaccggatgacctgattgaaa cggttgcgcgcgatctgcgcctgcgcgatctccagctcacacacgagttcatcaatccaagctggcaggc tccaaccatccgccgcctgacgcgcgacatggacaaggccttgcagcgtaccggtgttcgcgtcacttcg ggcatgaccggcccctatggccgcctcaaccatttcggccatccggacgcagaagtgcgccgttactatg tcgactggttcaagaccttcgccgacattatcggcgatcttggcggcaagtcggtcggcacgcagtttgcg atcttcacctataaggatttcgatgacgctgcgcgcccgcgaagatctgatcaagatcgccatcgattgctgg gcggaagtcgccgaacatgcgagccgtgccggtctcgactatgtgttctgggaaccgatgagcatcggg cgtgaatttggcgagacgattgccgaatgcatgaagcttcaggatcgcctgaccgccgccgacatggcta ttccgatgtggatgatggccgatatcgaccacggcgatgtgacttccgccaatccggacgatttcgatccct acgcctgggcacgcgccgtgccgaaagtctcgccgatcattcacatcaagcagagcctgatggacaag ggcggacatcgtcctttcacagccgcgttcaatgccaagggacgcatccagccggaaccgcttctgaaa gcctttgccgaaggcggcgctgtggacaatgaaatctgcctcgaactgtcgttcaaggagcgcgagccg aatgaccgtgaagtcattccgcagattgcggaaagtgtggctttctgggctccgcacattgacaccggcgc taaggacttgaagatataa	This work
eryD	CDS	atggcagatgctgacgattcactggcgctgcgcgccgcatggcttcacttcgtggcgggcatgacccagtc agccgttgcgaagcgccttggtctgccctccgtgaaagcgcatcgccttattgccaaagccgtcgcggac ggtgcggtgaaggtcagtatcgacggcgacattaccgaatgtatcgacctcgaaaatcgtctcgccgatat gtacggcctcgactattgcgaagtcgttcccgacatcggtgaggaaggtctgccgctcatggcgcttggcc atgcgggtgcagattttctgcgccgcgagatcgagcatggcgaccatgaagtcatcggcatcggccatgg ccgcacgctttcggctgcagtcaactatatgccgcgcgtcgtcgccaatgatctgcgcttcgtgtcgctgctc ggcggcctgacgcgcaatttcgccgccaacccccatgacgttatgcaccgtatcgccgaaaagaccggt atgcctgcttatgtgatgccggtgcccttcttcgccaacacggcggaagaccgcgaagtgctgctcgcaca gcgcggcgtcaccaccgttttcgacatgggttgccaagccgaactgaagatcgtcggcatcggcactgtc gatgcgcaggcgcagcttgtcacatccggcatgatagaactggccgaggtcgaagagatcgccagcct cggcggtgttggcgaaatgctcggccatttctttgacgccaatggccagcgccttgaaaccgcgctgaccg cccgcaccatcgctgcatcagtcgaaaacgcggatatgagccgaatcgtgggccttgccggtggaatttc caaggcggaagccatccgcgctgtgctgaaaagcggtcgtctttacggtctcatcaccgatgaacgcac ggcaaagacgctcgttgagcagccgaacggaaaataa	This work
eryE	CDS	atgagtgcacaaaccagtcaagtttcacaaaaacccgccaagctgagccgcgccgtcgtctggagcgt gatcgctgcgatcgtggttgtcggtgcgattgccttcgacacgaaggtcgtgaagatcgggtctgattccga tgtgcgccagcaggctttctcccccgatgcctacggtgcttccgaattccccaaggtgaaggcgagcgttg agcagcgcgccgtggatgcagtggaagttggaacggctctcgccgccgacaaggccgccgccggaa agaaatatggcgtcggcgacgtcaatccggttgtcccggtcaagtttacaggcaccgttgaggagcgcaa atccaactacaatgtcgtgaaggtcgacggcctgccggaaggcgttgccatccgcgttcagaccggccct gccgtcaacggcaccgatctgcgcgatgcgaccggtgaaatccagttcggccagttcaagaaccagatc gaatatcaaaatgccggttctgccctgaacaacgagatgaagaagcaagtgctttccggcgtcgatgtcg agaatctcgtcggcaagaccgtgacggtggttggcgtgttcaaggtcgtcaatccgaagaactggctcgtc acgcctgtggggctcgaagtgaaatga	This work
eryF	CDS	atgagcaccgcttcgaaaatcgaaggcaaaaacggtgatgtcgttcttgccgcaaacaacgttgccaaat cctatggtcgcattcatgccctgaagggcgtgaatttcgagattcgccgtggtcaggtcacgacgcttttcgg cgaaaacggcgctggcaaatcaacgttgatgaaagtgctttcgggcgtcatccagccgacttcggggac gatcattctcgatggcgaacctgtgacgttcaattcgtccaccgaagcgcgcgacctcggcatctcgatcat	This work

		ccatcaggaattgagcctcgcgcccaacatgaacgtgcgcgacaacatcttcatggggcgcgaaatccgcaccgccaccggcgttgattttgccgaggaagaacgcctgacgcgcgcgcttctgaaggaactggaagaagacatcgacccgctgacgccggtcgaggaacttcgtctcggccagcagcaggttgttgaaatcgcccgcgccctttcggtcaattcgcgcattctcatcatggatgagccgacttcggcgctcagcgcctcggaagtggaagtcctgttcaaggtcattcgcgacctgacggcgcgcggtgtcgccatcgtctacatttcgcatcatctggaagaagcgttgcagatcaccaatcatgcggtggtgctgcgcgacggaaccatgacggcctatgcgccgcgtgaggaaattgatctggaatggatcgtgcgcaacatggtcggcgagaacttcgatctcggctcgcctccaaccggatatgactggggcgatgtggcgctctctgttgagaacctgaccgttcccgacccgggtggcgcgggcttctcgctggtcgaccgcatgtcgctcaatgtacgcgccggtgaaatcgtctgcatttatggtcttatgggcgccggtcgcacagaactgctggaaaccgttgccggtcgtctcaaggcaagcggcggacgtgttctcctcaaagggcaggacgtttccggcctcaccatcgcacaacgtatcgagaagggtcttgtgctggtgccggaagatcgccagcgcgacggtctcgtccagacgatgacggtcggcaagaacctgtcgctcgccagcatcgccgaaatgaccaagggtctgttcacatcgcgcaagcgtgaaaagcagattgtcgatcagtcgatcaagaatgtgcacatcaagacggatggcggcgaagcagcaatcggttcgctttccggtggtaaccagcagaaggtcgtgatcggcaagatgctggcgaccgaaccggaagtcatcctgcttgatgagccgagccgcgggatcgacatcggggcgaaggcggaagtgttcaagcttctggctgaaaaggcgaagcagggtctggctgtcgtctacacgacttcggaagtcggcgaatgcctcagcatcgctcatcgtatcatcgtcatgcaccgtggacgcatctctgccgaattcggatcggacgtgtccaaggagaagatcatggccgcctcgggcgaagccatggtcggtcactaa	
eryG	CDS	atgctctggttatggagcactgatagaatgtcagtcacgagcaccaacaaagaaacagcggctccgaacggcgcgaagcggaaacccggcatcgtgcatctgctgctcgaaggtcgtgcattttttgcactgatcgcgatcattgcagttttctcgttcctttcgccttattatttctcggtcgataacttcctcatcatgtcgtcgcacgtggccatttcggcctgcttgccatcggcatgctgctcgtcatcctcaatggcggcatcgatctttccgttggctccacattggggctggccggtgtggtcgccgggttcctgatgcagggcgtgacgctgcaggaattcggcatcattctctatttcccggtctgggccgtggttctcatcacctgtgcgctcggcgcattcgtcggcgcggtcaatggtgtgctcatcgcctatctgcgcgttccggccttcgttgcgacgctcggcgttctctattgcgcgcgtggcgtggcgcttctgatgaccaatggcctgacctacaacaatctcggcggacgtccggagcttggcaatacaggctttgactggctcggtttcaacaagctgttcggcgtgccgatcggcgttctggttctggcggttctcgccatcatctgcgccatcgttctcaaccgcaccgcatttggtcgctggctctatgcgtcgggtggcaacgaacgtgcggctgaactgtccggcgttccggtcaagcgcgtcaaggtgtcggtctatgtcatttccggcatctgcgctgccattgcaggtctggttctttcttcgcagctgacgtcggccggtccgacggcaggcaccacctacgaactgacggcaatcgctgccgtggttatcggtggcgctgcgctcactgggggggcgcggtactatccagggcacacttctcggcgctttcgtgatcggtttcctttccgacggcctcgtgattatcggcgtgtcctcctactggcagaccgtctttaccggcgcggtaatcgtgctcgcggttctgctcaacagcattcaatactcccggcgatga	This work
eryH	CDS	atgacaaaattctggattggcaccagctggaagatgaacaagacgctggccgaggcccgcattttcgccgaagccttgaaagctgccgatgcaagccgctcgaccgacattcagcgtttcgtcatcccgccctttaccgccgtgcgcgaagtgaaggaaatcctgttcggcacctccgtcaaggtcggcgcgcagaacatgcattgggccgatcagggagcatggaccggcgagatttcgccgctgatgctcaaggactgcaatctcgatatcgtcgaactcggtcattccgagcgccgtgaacatttcggtgaaaccaacgaaacggtcggcctcaaggtcgaagctgcggtgcgccacggcctgatcccactcatctgcatcggtgagacgctggaagaccgcgaaagcggacgcgccgcggaagttcttgaggaagaagtgcgcggtgcactttccaagctttccggtgaccagaagcaggcggaaatcctgtttgcctatgagccggtctgggccatcggcgaaaatggtatcccggcatcggcggaatatgccgatgcgcgccaggcggaaatcatcgcggttgcagaaagcgtactcggtcgtcgtgtgccttgcctctatggcggctcggtcaatccgggcaattgcgaagagctgatcgcttgcccgcacattgacgggcttttcatcggtcgctcggcatggaacgtcgaaggttatctcgacattctggccaagtgtgccgccaagactcgagctaattaa	This work

eryI	CDS	atgatgaaagtagcagtagcaggcgacagcgccggcgaaggtctggccaaggttctcgccgatcacctcaaggatcgcttcgaggtttcggaaatctcgcgtacagacgctggcgcagatacattctacgccaacctctccgaccgcgtggcttccgccgttctcgacggcacctatgaccgcgccatcctcgtttgcggcaccggcatcggcgtatgcattgcagccaataaggttccgggcatccgcgccgcgctgacgcacgacacctattcggcagagcgcgcagcgctttccaacaatgcccagatcatcaccatgggcgcccgcgtcatcggctcggaagtagccaagaccattgccgacgccttccttgctgagacctttgacgaaaacggccgttcggctggcaatgtcaacgcgatcaacgaagttgacgcgaagtacaacaagggctga	This work
eryR	CDS	atgacgatccatcgcgatctggattcgctggaacataccggcgtgctgcgcaaagtacgcggcggcgcgacaatcgataccggcacacaatttgaaagcgatttccgcttccgcgaacggcaggacggcgaagcgaagcagaaaatggcccgcgccgcgcttgagcttgttgaacccggcatgacggtgatgattaatgacggctccatggcggctgtccttggcggcatgcttctggaaaagcgcccgctgacagtcatcaccaacaatgccgtcatcatcgatgaactgaagggcgagaacgggatcaatctgattgcgctcggcggaacctattcagccaagttcaatgcgtttttcggcatcctgacggaaggggccctgtcgcatctgagcgccgacatcgccttcatttcctcgcctgcagtcaatggcaagctcgtctatcacatggatgagaatgttgttcgcaccaagcgcgcgatgacggcatccgccgcccgcacctgccttctggtcaatcaccagcggttcggacgccctgccctgcatgtcatggctgatctttccgatttcgacgcgatcataaccgatgcctcccccgacgcgaccgtactggccgatcttgaacaggcgggcattgcactgaccatcgctgacgactgcgcataa	This work
sfGFP	CDS	atgcgtaaaggcgaagagctgttcactggtgtcgtccctattctggtggaactggatggtgatgtcaacggtcataagttttccgtgcgtggcgagggtgaaggtgacgcaactaatggtaaactgacgctgaagttcatctgtactactggtaaactgccggtaccttggccgactctggtaacgacgctgacttatggtgttcagtgctttgctcgttatccggaccatatgaagcagcatgacttcttcaagtccgccatgccggaaggctatgtgcaggaacgcacgatttcctttaaggatgacggcacgtacaaaacgcgtgcggaagtgaaatttgaaggcgataccctggtaaaccgcattgagctgaaaggcattgactttaaagaagacggcaatatcctgggccataagctggaatacaattttaacagccacaatgtttacatcaccgccgataaacaaaaaaatggcattaaagcgaattttaaaattcgccacaacgtggaggatggcagcgtgcagctggctgatcactaccagcaaaacactccaatcggtgatggtcctgttctgctgccagacaatcactatctgagctaccaaagcgttctgtctaaagatccgaacgagaaacgcgatcatatggttctgctggagttcgtaaccgcagcgggcatcacgcatggtatggatgaactgtactaa	2
eryE-sfGFP-6xHis	CDS	atgagtgcacaaaccagtcaagtttcacaaaaacccgccaagctgagccgcgccgtcgtctggagcgtgatcgctgcgatcgtggttgtcggtgcgattgccttcgacacgaaggtcgtgaagatcgggtctgattccgatgtgcgccagcaggctttctcccccgatgcctacggtgcttccgaattccccaaggtgaaggcgagcgttgagcagcgcgccgtggatgcagtggaagttggaacggctctcgccgccgacaaggccgccgccggaaagaaatatggcgtcggcgacgtcaatccggttgtcccggtcaagtttacaggcaccgttgaggagcgcaaatccaactacaatgtcgtgaaggtcgacggcctgccggaaggcgttgccatccgcgttcagaccggccctgccgtcaacggcaccgatctgcgcgatgcgaccggtgaaatccagttcggccagttcaagaaccagatcgaatatcaaaatgccggttctgccctgaacaacgagatgaagaagcaagtgctttccggcgtcgatgtcgagaatctcgtcggcaagaccgtgacggtggttggcgtgttcaaggtcgtcaatccgaagaactggctcgtcacgcctgtggggctcgaagtgaaaatgcgtaaaggcgaagagctgttcactggtgtcgtccctattctggtggaactggatggtgatgtcaacggtcataagttttccgtgcgtggcgagggtgaaggtgacgcaactaatggtaaactgacgctgaagttcatctgtactactggtaaactgccggtaccttggccgactctggtaacgacgctgacttatggtgttcagtgctttgctcgttatccggaccatatgaagcagcatgacttcttcaagtccgccatgccggaaggctatgtgcaggaacgcacgatttcctttaaggatgacggcacgtacaaaacgcgtgcggaagtgaaatttgaaggcgataccctggtaaaccgcattgagctgaaaggcattgactttaaagaagacggcaatatcctgggccataagctggaatacaattttaacagccacaatgtttacatcaccgccgataaacaaaaaatggcattaaagcgaattttaaaattcgccacaacgtggaggatggcagcgtgcagctggctgatcactaccagcaaaacactccaatcggtgatggtcctgttctgctgccagacaatcactatctgagctaccaaag	This work

		cgttctgtctaaagatccgaacgagaaacgcgatcatatggttctgctggagttcgtaaccgcagcgggcatcacgcatggtatggatgaactgtaccatcatcatcatcatcactaataa	
eryG-sfGFP-6xHis	CDS	atgctctggttatggagcactgatagaatgtcagtcacgagcaccaacaaagaaacagcggctccgaacggcgcgaagcggaaacccggcatcgtgcatctgctgctcgaaggtcgtgcatttttttgcactgatcgcgatcattgcagttttctcgttcctttcgccttattatttctcggtcgataacttcctcatcatgtcgtcgcacgtggccattttcggcctgcttgccatcggcatgctgctcgtcatcctcaatggcggcatcgatctttccgttggctccacattggggctggccggtgtggtcgccgggttcctgatgcagggcgtgacgctgcaggaattcggcatcattctctatttcccggtctgggccgtggttctcatcacctgtgcgctcggcgcattcgtcggcgcggtcaatggtgtgctcatcgcctatctgcgcgttccggccttcgttgcgacgctcggcgttctctattgcgcgcgtggcgtggcgcttctgatgaccaatggcctgacctacaacaatctcggcggacgtccggagcttggcaatacaggctttgactggctcggtttcaacaagctgttcggcgtgccgatcggcgttctggttctggcggttctcgccatcatctgcgccatcgttctcaaccgcaccgcatttggtcgctggctctatgcgtcgggtggcaacgaacgtgcggctgaactgtccggcgttccggtcaagcgcgtcaaggtgtcggtctatgtcatttccggcatctgcgctgccattgcaggtctggttctttcttcgcagctgacgtcggccggtccgacggcaggcaccacctacgaactgacggcaatcgctgccgtggttatcggtggcgctgcgctcactgggggcgcggtactatccagggcacacttctcggcgctttcgtgatcggtttcctttccgacggcctcgtgattatcggcgtgtcctcctactggcagaccgtctttaccggcgcggtaatcgtgctcgcggttctgctcaacagcattcaatactcccggcgaatgcgtaaaggcgaagagctgttcactggtgtcgtccctattctggtggaactggatggtgatgtcaacggtcataagttttccgtgcgtggcgagggtgaaggtgacgcaactaatggtaaactgacgctgaagttcatctgtactactggtaaactgccggtaccttggccgactctggtaacgacgctgacttatggtgttcagtgctttgctcgttatccggaccatatgaagcagcatgacttcttcaagtccgccatgccggaaggctatgtgcaggaacgcacgatttcctttaaggatgacggcacgtacaaaacgcgtgcggaagtgaaatttgaaggcgataccctggtaaaccgcattgagctgaaaggcattgactttaaagaagacggcaatatcctgggccataagctggaatacaattttaacagccacaatgtttacatcaccgccgataaacaaaaaaatggcattaaagcgaattttaaaattcgccacaacgtggaggatggcagcgtgcagctggctgatcactaccagcaaaacactccaatcggtgatggtcctgttctgctgccagacaatcactatctgagctaccaaagcgttctgtctaaagatccgaacgagaaacgcgatcatatggttctgctggagttcgtaaccgcagcgggcatcacgcatggtatggatgaactgtaccatcatcatcatcatcactaataa	This work
eryD binding site (eryO)	protein binding site	gaaaaaaaatgcgccatctagaaaatttt	This work

Supplementary Table 3. Gene clusters.

Cluster name	Annotation	DNA sequence	Reference
original erythritol cluster	including all erythritol-associated promoters, RBSs, CDSs (eryA/B/C/D/E/F/G/H/I/R) and terminators	tcatcgccgggagtattgaatgctgttgagcagaaccgcgagcacgattaccgcgccggtaaagacggtctgccagtagg<br>aggacacgccgataatcacgaggccgtcggaaaggaaaccgatcacgaaagcgccgagaagtgtgccctggatagta<br>ccgcgccccccagtgagcgcagcgccaccgataaccacggcagcgattgccgtcagttcgtaggtggtgcctgccgtcg<br>gaccggccgacgtcagctgcgaagaaagaaccagacctgcaatggcagcgcagatgccggaaatgacatagaccga<br>caccttgacgcgcttgaccggaacgccggacagttcagccgcacgttcgttgccacccgacgcatagagccagcgacca<br>aatgcggtgcggttgagaacgatggcgcagatgatggcgagaaccgccagaaccagaacgccgatcggcacgccga<br>acagcttgttgaaaccgagccagtcaaagcctgtattgccaagctccggacgtccgccgagattgttgtaggtcaggccatt<br>ggtcatcagaagcgccacgccacgcgcgcaatagagaacgccgagcgtcgcaacgaaggccggaacgcgcagata<br>ggcgatgagcacaccattgaccgcgccgacgaatgcgccgagcgcacaggtgatgagaaccacggcccagaccggg<br>aaatagagaatgatgccgaattcctgcagcgtcacgccctgcatcaggaacccggcgaccacaccggccagccccaat<br>gtggagccaacggaaagatcgatgccgccattgaggatgacgagcagcatgccgatggcaagcaggccgaaaatggc<br>cacgtgcgacgacatgatgaggaagttatcgaccgagaaataataaggcgaaaggaacgagaaaactgcaatgatcg<br>cgatcagtgcaaaaaatgcacgaccttcgagcagcagatgcacgatgccgggtttccgcttcgcgccgttcggagccgct<br>gtttctttgttggtgctcgtgactgacattctatcagtgctccataaccagagcatttaacaacccgactttgtcgggtcttatgctct<br>atccttttgtttttaacgcataatcttgtccgaaaagtctgcaacttttcgggattatgttttagtgaccgaccatggcttcgcccga<br>ggcggccatgatcttctccttggacacgtccgatccgaattcggcagagatgcgtccacggtgcatgacgatgatacgatga<br>gcgatgctgaggcattcgccgacttccgaagtcgtgtagacgacagccagaccctgcttcgccttttcagccagaagcttga<br>acacttccgccttcgccccgatgtcgatcccgcggctcggctcatcaagcaggatgacttccggttcggtcgccagcatcttg<br>ccgatcacgaccttctgctggttaccaccggaaagcgaaccgattgctgcttcgccgccatccgtcttgatgtgcacattcttg<br>atcgactgatcgacaatctgcttttcacgcttgcgcgatgtgaacagacccttggtcatttcggcgatgctggcgagcgacag<br>gttcttgccgaccgtcatcgtctggacgagaccgtcgcgctggcgatcttccggcaccagcacaagacccttctcgatacgtt<br>gtgcgatggtgaggccggaaacgtcctgccctttgaggagaacacgtccgccgcttgccttgagacgaccggcaacggttt<br>ccagcagttctgtgcgaccggcgcccataagaccataaatgcagacgatttcaccggcgcgtacattgagcgacatgcgg<br>tcgaccagcgagaagcccgcgccacccgggtcgggaacggtcaggttctcaacagagagcgccacatcgccccagtc<br>atatccggttggaggcgagccgagatcgaagttctcgccgaccatgttgcgcacgatccattccagatcaatttcctcacgc<br>ggcgcataggccgtcatggttccgtcgcgcagcaccaccgcatgattggtgatctgcaacgcttcttccagatgatgcgaaa<br>tgtagacgatggcgacaccgcgcgccgtcaggtcgcgaatgaccttgaacaggacttccacttccgaggcgctgagcgc<br>cgaagtcggctcatccatgatgagaatgcgcgaattgaccgaaagggcgcgggcgatttcaacaacctgctgctggccg<br>agacgaagttcctcgaccggcgtcagcgggtcgatgtcttcttccagttccttcagaagcgcgcgcgtcaggcgttcttcctcg<br>gcaaaatcaacgccggtggcggtgcggatttcgcgccccatgaagatgttgtcgcgcacgttcatgttgggcgcgaggctc<br>aattcctgatggatgatcgagatgccgaggtcgcgcgcttcggtggacgaattgaacgtcacaggttcgccatcgagaatg<br>atcgtccccgaagtcggctggatgacgcccgaaagcactttcatcaacgttgatttgccagcgccgttttcgccgaaaagcg<br>tcgtgacctgaccacggcgaatctcgaaattcacgcccttcagggcatgaatgcgaccataggatttggcaacgttgtttgcg<br>gcaagaacgacatcaccgtttttgccttcgattttcgaagcggtgctcatttcacttcgagccccacaggcgtgacgagccag<br>ttcttcggattgacgaccttgaacacgccaaccaccgtcacggtcttgccgacgagattctcgacatcgacgccggaaagc<br>acttgcttcttcatctcgttgttcagggcagaaccggcattttgatattcgatctggttcttgaactggccgaactggatttcaccg<br>gtcgcatcgcgcagatcggtgccgttgacggcagggccggtctgaacgcggatggcaacgccttccggcaggccgtcga<br>ccttcacgacattgtagttggatttgcgctcctcaacggtgcctgtaaacttgaccgggacaaccggattgacgtcgccgacg<br>ccatatttctttccggcggcggccttgtcggcggcgagagccgttccaacttccactgcatccacggcgcgctgctcaacgct<br>cgccttcaccttggggaattcggaagcaccgtaggcatcgggggagaaagcctgctggcgcacatcggaatcagacccg<br>atcttcacgaccttcgtgtcgaaggcaatcgcaccgacaaccacgatcgcagcgatcacgctccagacgacggcgcggc<br>tcagcttggcgggtttttgtgaaacttgactggtttgtgcactcattatcaaacgccttcagcaattctgccgtattgtcgttatgga<br>aacggatgatgcggctgaattagaccgcccggagattaccctcgaccattgtcatcacgcttaccggataggcaaaagcct	This work

		ccgccaaagcggtgcaggacgcgacagacgagatcatgatagcctccacatgaattccagggccccgctcctccgtgca<br>tcctggaaacagatgcgccgcaatcaaaacctatgatagcggcgtgttattaactccaataatgatgttataaagctcgaaa<br>gctgtcaatcggatgttttcgaaacaccggtgaaatagcacaatttcgcgccgaatctcgatcacgatctagagaaatccac<br>tacataattcgttatcaatcgcactattcacagcaccgtcttttctcgcaaagccagccacaaagatactcacaaacttctatat<br>tttcttttattttcaatgaattaaacatatttaaaaccgaagaaacattaagctattgatggaaaacagtgggaaaaccgcactc<br>gaaggcgcggagatttcccctacattcactctttggtgaaaaaaaatgcgccatctagaaaattttacagacaacgtgatag<br>cgttatgttatctccagcatacgccatcgcccgatttcattataaacagggctgcgagcgacgtattttcggtcgcgggagga<br>atccaaatgtcaatgaaagcacatagccgccatgcgtgagaaaggtgacatcatcatcgggatcgacgcgggcacgtct<br>gtgctcaaggcagtcgctttcgaccttagcggacgccagatcgaatctgctgccgttcgcaataaatacgataccggcgaa<br>catggcgctgtcacacagtcgctttcccagacgtggcaggactgcgcgcgcgccttgcgcgacctcggtgcaaagatgcc<br>cggccttgccgaacgcactgcggcaatcgccgttaccggtcagggtgacggaacctggcttgttggccgcgacaaccagc<br>cggtcggcgatgcctggctgtggctcgatgcccgcgcggcaacgacggtgacgcgcctcgccgctggccccgtgaaccg<br>cgcgcgctttgaagcgaccggcactgggttgaacacctgccagcaaggctcgcagatggcgcatatggacagcatcgcg<br>cccgagctgctggacaatgccgaagtcgcgctccactgcaaagactggctctatctcaatctcaccggcgttcgcgccacc<br>gacccttcggaagcaagcttcaccttcggcaatttccgcaatcgccagtatgacgatgtcgtcatcgacgcgctcggtctga<br>ggaaacggcgtggattgctgccggaaatcatcgacggcaccgaagtgcagcatccgctatcccccgaagcggcagctg<br>caacgggtttgcgggcagggacgccggtgggtctcgcctatgtcgatatggccatgacagcacttggtgcgggcgtgcgc<br>ggcggcacagcaggcgccgggtgctcgaccattggttcgacaggcgtccatatgcgcgccaagccggttgccgatatcc<br>acctgaacaaggaaggcaccggttacgtcatcgcgctgcccattcccggcatcgtcacccaggtgcagaccaatatggg<br>ggcaacgatcaacatcgactggatattgcaggttgctgccgatctcatgtcgacgccagacaagcctgtttcgctcagtgac<br>ctcattccccgccttgatgactggtttaatgccagccgtccgggtgcaatcctctatcatccgtatatttcggaagcgggcgaa<br>cgcggtcccttcgtcaatgcctatgcccgggccggtttcgtcggtctttccagccgcgaccgtttcccggaaatggtgcgctcg<br>gttgtcgagggcctcggaatggcgacgcgcgattgctacacggccatgggtgaaatgcccgcagaattgcgcatcaccgg<br>cggtgcagcgcgttcaaaagcattgcgcagcacccttgccgcagcggtgaatgcgcccgtgcgcgtttcctcgcgtgaag<br>aggcgggagccgcaggtgctgccatgatggctgcggtggccattggcgcttatccaagcatggatgcatgtattgcggaat<br>gggtcgagccgctgcttggcccttgcgaggccccggatggtccgcgcgcaaagcactatgaagaactcttcgttgcctatc<br>aggaagcccggctggcactgacgcccgtctgggacaagttggcttccgatagagcatttccagcaaaagcgtgaagcgg<br>ttttgcgttcggaaatgcggtgaacaaaacgaataaggccggaccttccttccggcacatatagactatgggatgcaattgc<br>atccgaaggagcatgaaatggctgaaccggaaacctgcgatctctttgtcatcggcggtggtatcaacggcgcgggcgtg<br>gcccgtgacgccgccgggcgcggcctcaaggtggtgctggcggaaaaagacgatctggcgcagggaacttcgtcacgc<br>tccggcaagctggtacatggtggtctgcgttatctcgaatattatgagtttcgccttgtgcgcgaagcgctgatcgagcgcgaa<br>gtgctgttgaacgccgctccccatatcatctggccaatgcgtttcgttctgccgcacagcccgcaggaccgtcccgcctggct<br>ggtgcggcttggcctgtttctctatgaccacctcggcggccgcaagaagcttcccggcacacgcacgctcgacctgcggcg<br>cgatccggaaggcacgccgatcctcgaccaatataccaagggcttcgaatattccgactgttgggtcgatgatgcccgtctc<br>gttgcattgaatgcggttggcgctgctgaaaaaggcgcgaccattctgacccgcacgcctgtcgtctccgctcgtcgcgaaa<br>aaggcggctggatcgtggaaacgaaaaaccgcgacacgggcgaaacccgcacattccgcgcgcgctgcatcgtcaatt<br>gcgccggaccatgggtcacggatgtcatccacaatgtcgccggctccaactcgtcgcgcaatgtgcgtctcgtcaagggca<br>gccacatcatcgttccgaaattctggtcgggcgcaaatgcctatctcgtccagaaccacgacaagcgcgttatctttatcaat<br>ccctacgagggcgacaaggcgctgatcggcaccaccgacatcgcctatgaaggccgtgccgaagatgttgcagcagat<br>gagaaggaaatcgaatatctgctgactgcagtgaaccgctatttcaaggaaaagctcaggcgtgaagacgtgctgcattcc<br>ttctccggtgtgcgtccgctgttcgacgacggcaagggcaacccttccgccgtcacccgcgactatgtcttcgacctcgatga<br>aaccaacgacgcgccgctgctcaacgtctttggcggcaagatcaccaccttccgcgagctggccgagcgcggcatgcat<br>cgcctcaagcacattttcccgaagatgggcggcgactggactcatggcgcaccgcttccgggcggcgaaatcgccaatgc<br>cgattatgaaaccttcgccaactccttgcgcgacacctatccgtggatgccgcgcgcgctcgtccaacactacggtcgtctct<br>acggcgcacgcacgaaagacgttgttgcaggcgcacagaacctcgaagggctcggccgtcatttcggcggcaatttccat<br>gaggcggaagtgcgctatctggtggccagggaatgggcaatgacggcggaagacattctctatcgccgcaccaagcact	

		atctgcacctgaccgaagccgaacgcgccgctttcgtggaatggttcgacaacgctaaaatggtctcttgaggacactatgg<br>cccttacgctttctctgaacaccaatccacttgtgaaccgctttgccgaaccggatgacctgattgaaacggttgcgcgcgatc<br>tgcgcctgcgcgatctccagctcacacacgagttcatcaatccaagctggcaggctccaaccatccgccgcctgacgcgc<br>gacatggacaaggccttgcagcgtaccggtgttcgcgtcacttcgggcatgaccggcccctatggccgcctcaaccatttcg<br>gccatccggacgcagaagtgcgccgttactatgtcgactggttcaagaccttcgccgacattatcggcgatcttggcggcaa<br>gtcggtcggcacgcagtttgcgatcttcacctataaggatttcgatgacgctgcgcgccgcgaagatctgatcaagatcgcc<br>atcgattgctgggcggaagtcgccgaacatgcgagccgtgccggtctcgactatgtgttctgggaaccgatgagcatcggg<br>cgtgaatttggcgagacgattgccgaatgcatgaagcttcaggatcgcctgaccgccgccgacatggctattccgatgtgg<br>atgatggccgatatcgaccacggcgatgtgacttccgccaatccggacgatttcgatccctacgcctgggcacgcgccgtg<br>ccgaaagtctcgccgatcattcacatcaagcagagcctgatggacaagggcggacatcgtcctttcacagccgcgttcaat<br>gccaagggacgcatccagccggaaccgcttctgaaagcctttgccgaaggcggcgctgtggacaatgaaatctgcctcg<br>aactgtcgttcaaggagcgcgagccgaatgaccgtgaagtcattccgcagattgcggaaagtgtggctttctgggctccgc<br>acattgacaccggcgctaaggacttgaagatataaacgatttcaggaaccggaccgctttcaatggcagatgctgacgatt<br>cactggcgctgcgcgccgcatggcttcacttcgtggcgggcatgacccagtcagccgttgcgaagcgccttggtctgccctc<br>cgtgaaagcgcatcgccttattgccaaagccgtcgcggacggtgcggtgaaggtcagtatcgacggcgacattaccgaat<br>gtatcgacctcgaaaatcgtctcgccgatatgtacggcctcgactattgcgaagtcgttcccgacatcggtgaggaaggtctg<br>ccgctcatggcgcttggccatgcgggtgcagattttctgcgccgcgagatcgagcatggcgaccatgaagtcatcggcatc<br>ggccatggccgcacgctttcggctgcagtcaactatatgccgcgcgtcgtcgccaatgatctgcgcttcgtgtcgctgctcgg<br>cggcctgacgcgcaatttcgccgccaacccccatgacgttatgcaccgtatcgccgaaaagaccggtatgcctgcttatgtg<br>atgccggtgcccttcttcgccaacacggcggaagaccgcgaagtgctgctcgcacagcgcggcgtcaccaccgttttcga<br>catgggttgccaagccgaactgaagatcgtcggcatcggcactgtcgatgcgcaggcgcagcttgtcacatccggcatgat<br>agaactggccgaggtcgaagagatcgccagcctcggcggtgttggcgaaatgctcggccatttctttgacgccaatggcca<br>gcgccttgaaaccgcgctgaccgcccgcaccatcgctgcatcagtcgaaaacgcggatatgagccgaatcgtgggccttg<br>ccggtggaatttccaaggcggaagccatccgcgctgtgctgaaaagcggtcgtctttacggtctcatcaccgatgaacgca<br>cggcaaagacgctcgttgagcagccgaacggaaaataacataataacatttttgcaatggattattttgtgattttgtgatctaa<br>cttcgcgcagaaaatgttccgccaataagatcggacgagagcgtgaagcgcgacgacagaagacaggcgattataaac<br>cttctgatcgaaaatcaggccgtcgacctcgacgatctggcggatcggtttgccgtatcaaaaatgacgatccatcgcgatct<br>ggattcgctggaacataccggcgtgctgcgcaaagtacgcggcggcgcgacaatcgataccggcacacaatttgaaagc<br>gatttccgcttccgcgaacggcaggacggcgaagcgaagcagaaaatggcccgcgccgcgcttgagcttgttgaacccg<br>gcatgacggtgatgattaatgacggctccatggcggctgtccttggcggcatgcttctggaaaagcgcccgctgacagtcat<br>caccaacaatgccgtcatcatcgatgaactgaagggcgagaacgggatcaatctgattgcgctcggcggaacctattcag<br>ccaagttcaatgcgtttttcggcatcctgacggaaggggccctgtcgcatctgagcgccgacatcgccttcatttcctcgcctg<br>cagtcaatggcaagctcgtctatcacatggatgagaatgttgttcgcaccaagcgcgcgatgacggcatccgccgcccgc<br>acctgccttctggtcaatcaccagcggttcggacgccctgccctgcatgtcatggctgatctttccgatttcgacgcgatcataa<br>ccgatgcctcccccgacgcgaccgtactggccgatcttgaacaggcgggcattgcactgaccatcgctgacgactgcgca<br>taaacctagaggacgaaaatcgagcatgacaaaattctggattggcaccagctggaagatgaacaagacgctggccga<br>ggcccgcattttcgccgaagccttgaaagctgccgatgcaagccgctcgaccgacattcagcgtttcgtcatcccgcccttta<br>ccgccgtgcgcgaagtgaaggaaatcctgttcggcacctccgtcaaggtcggcgcgcagaacatgcattgggccgatca<br>gggagcatggaccggcgagatttcgccgctgatgctcaaggactgcaatctcgatatcgtcgaactcggtcattccgagcg<br>ccgtgaacatttcggtgaaaccaacgaaacggtcggcctcaaggtcgaagctgcggtgcgccacggcctgatcccactc<br>atctgcatcggtgagacgctggaagaccgcgaaagcggacgcgccgcggaagttcttgaggaagaagtgcgcggtgca<br>ctttccaagctttccggtgaccagaagcaggcggaaatcctgtttgcctatgagccggtctgggccatcggcgaaaatggta<br>tcccggcatcggcggaatatgccgatgcgcgccaggcggaaatcatcgcggttgcagaaagcgtactcggtcgtcgtgtg<br>ccttgcctctatggcggctcggtcaatccgggcaattgcgaagagctgatcgcttgcccgcacattgacgggcttttcatcggt<br>cgctcggcatggaacgtcgaaggttatctcgacattctggccaagtgtgccgccaagactcgagctaattaaggagcaata<br>tgatgaaagtagcagtagcaggcgacagcgccggcgaaggtctggccaaggttctcgccgatcacctcaaggatcgcttc	

		gaggtttcggaaatctcgcgtacagacgctggcgcagatacattctacgccaacctctccgaccgcgtggcttccgccgttct<br>cgacggcacctatgaccgcgccatcctcgtttgcggcaccggcatcggcgtatgcattgcagccaataaggttccgggcat<br>ccgcgccgcgctgacgcacgacacctattcggcagagcgcgcagcgctttccaacaatgcccagatcatcaccatgggc<br>gcccgcgtcatcggctcggaagtagccaagaccattgccgacgccttccttgctgagacctttgacgaaaacggccgttcg<br>gctggcaatgtcaacgcgatcaacgaagttgacgcgaagtacaacaagggctga	
programmed erythritol catabolism cluster	including all erythritol catabolism-associated promoters, RBSs, CDSs (eryA/B/C/H/I) and terminators	tatcaaacgccttcagcaattctgccgtattgtcgttatggaaacggatgatgcggctgaattagaccgcccggagattaccc<br>tcgaccattgtcatcacgcttaccggataggcaaaagcctccgccaaagcggtgcaggacgcgacagacgagatcatga<br>tagcctccacatgaattccagggccccgctcctccgtgcatcctggaaacagatgcgccgcaatcaaaacctatgatagcg<br>gcgtgttattaactccaataatgatgttataaagctcgaaagctgtcaatcggatgttttcgaaacaccggtgaaatagcaca<br>atttcgcgccgaatctcgatcacgatctagagaaatccactacataattcgttatcaatcgcactattcacagcaccgtcttttct<br>cgcaaagccagccacaaagatactcacaaacttctatattttcttttattttcaatgaattaaacatatttaaaaccgaagaaac<br>attaagctattgatggaaaacagtgggaaaaccgcactcgaaggcgcggagatttcccctacattcactctttggtgaaaaa<br>aaatgcgccatctagaaaattttacagacaacgtgatagcgttatgttatctccagcatacgccatcgcccgatttcattataa<br>acagggctgcgagcgacgtattttcggtcgcgggaggaatccaaatgtcaatgaaagcacatagccgccatgcgtgaga<br>aaggtgacatcatcatcgggatcgacgcgggcacgtctgtgctcaaggcagtcgctttcgaccttagcggacgccagatcg<br>aatctgctgccgttcgcaataaatacgataccggcgaacatggcgctgtcacacagtcgctttcccagacgtggcaggact<br>gcgcgcgcgccttgcgcgacctcggtgcaaagatgcccggccttgccgaacgcactgcggcaatcgccgttaccggtca<br>gggtgacggaacctggcttgttggccgcgacaaccagccggtcggcgatgcctggctgtggctcgatgcccgcgcggca<br>acgacggtgacgcgcctcgccgctggccccgtgaaccgcgcgcgctttgaagcgaccggcactgggttgaacacctgcc<br>agcaaggctcgcagatggcgcatatggacagcatcgcgcccgagctgctggacaatgccgaagtcgcgctccactgca<br>aagactggctctatctcaatctcaccggcgttcgcgccaccgacccttcggaagcaagcttcaccttcggcaatttccgcaat<br>cgccagtatgacgatgtcgtcatcgacgcgctcggtctgaggaaacggcgtggattgctgccggaaatcatcgacggcac<br>cgaagtgcagcatccgctatcccccgaagcggcagctgcaacgggtttgcgggcagggacgccggtgggtctcgcctat<br>gtcgatatggccatgacagcacttggtgcgggcgtgcgcggcggcacagcaggcgccgggtgctcgaccattggttcgac<br>aggcgtccatatgcgcgccaagccggttgccgatatccacctgaacaaggaaggcaccggttacgtcatcgcgctgccca<br>ttcccggcatcgtcacccaggtgcagaccaatatgggggcaacgatcaacatcgactggatattgcaggttgctgccgatct<br>catgtcgacgccagacaagcctgtttcgctcagtgacctcattccccgccttgatgactggtttaatgccagccgtccgggtgc<br>aatcctctatcatccgtatatttcggaagcgggcgaacgcggtcccttcgtcaatgcctatgcccgggccggtttcgtcggtctt<br>tccagccgcgaccgtttcccggaaatggtgcgctcggttgtcgagggcctcggaatggcgacgcgcgattgctacacggc<br>catgggtgaaatgcccgcagaattgcgcatcaccggcggtgcagcgcgttcaaaagcattgcgcagcacccttgccgca<br>gcggtgaatgcgcccgtgcgcgtttcctcgcgtgaagaggcgggagccgcaggtgctgccatgatggctgcggtggccatt<br>ggcgcttatccaagcatggatgcatgtattgcggaatgggtcgagccgctgcttggcccttgcgaggccccggatggtccgc<br>gcgcaaagcactatgaagaactcttcgttgcctatcaggaagcccggctggcactgacgcccgtctgggacaagttggctt<br>ccgatagagcatttccagcaaaagcgtgaagcggttttgcgttcggaaatgcggtgaacaaaacgaataaggccggacct<br>tccttccggcacatatagactatgggatgcaattgcatccgaaggagcatgaaatggctgaaccggaaacctgcgatctctt<br>tgtcatcggcggtggtatcaacggcgcgggcgtggcccgtgacgccgccgggcgcggcctcaaggtggtgctggcggaa<br>aaagacgatctggcgcagggaacttcgtcacgctccggcaagctggtacatggtggtctgcgttatctcgaatattatgagttt<br>cgccttgtgcgcgaagcgctgatcgagcgcgaagtgctgttgaacgccgctccccatatcatctggccaatgcgtttcgttct<br>gccgcacagcccgcaggaccgtcccgcctggctggtgcggcttggcctgtttctctatgaccacctcggcggccgcaaga<br>agcttcccggcacacgcacgctcgacctgcggcgcgatccggaaggcacgccgatcctcgaccaatataccaagggctt<br>cgaatattccgactgttgggtcgatgatgcccgtctcgttgcattgaatgcggttggcgctgctgaaaaaggcgcgaccattct<br>gacccgcacgcctgtcgtctccgctcgtcgcgaaaaaggcggctggatcgtggaaacgaaaaaccgcgacacgggcg<br>aaacccgcacattccgcgcgcgctgcatcgtcaattgcgccggaccatgggtcacggatgtcatccacaatgtcgccggct<br>ccaactcgtcgcgcaatgtgcgtctcgtcaagggcagccacatcatcgttccgaaattctggtcgggcgcaaatgcctatctc<br>gtccagaaccacgacaagcgcgttatctttatcaatccctacgagggcgacaaggcgctgatcggcaccaccgacatcgc<br>ctatgaaggccgtgccgaagatgttgcagcagatgagaaggaaatcgaatatctgctgactgcagtgaaccgctatttcaa	This work

		ggaaaagctcaggcgtgaagacgtgctgcattccttctccggtgtgcgtccgctgttcgacgacggcaagggcaacccttc<br>cgccgtcacccgcgactatgtcttcgacctcgatgaaaccaacgacgcgccgctgctcaacgtctttggcggcaagatcac<br>caccttccgcgagctggccgagcgcggcatgcatcgcctcaagcacattttcccgaagatgggcggcgactggactcatg<br>gcgcaccgcttccgggcggcgaaatcgccaatgccgattatgaaaccttcgccaactccttgcgcgacacctatccgtgga<br>tgccgcgcgcgctcgtccaacactacggtcgtctctacggcgcacgcacgaaagacgttgttgcaggcgcacagaacctc<br>gaagggctcggccgtcatttcggcggcaatttccatgaggcggaagtgcgctatctggtggccagggaatgggcaatgac<br>ggcggaagacattctctatcgccgcaccaagcactatctgcacctgaccgaagccgaacgcgccgctttcgtggaatggtt<br>cgacaacgctaaaatggtctcttgaggacactatggcccttacgctttctctgaacaccaatccacttgtgaaccgctttgccg<br>aaccggatgacctgattgaaacggttgcgcgcgatctgcgcctgcgcgatctccagctcacacacgagttcatcaatccaa<br>gctggcaggctccaaccatccgccgcctgacgcgcgacatggacaaggccttgcagcgtaccggtgttcgcgtcacttcg<br>ggcatgaccggcccctatggccgcctcaaccatttcggccatccggacgcagaagtgcgccgttactatgtcgactggttca<br>agaccttcgccgacattatcggcgatcttggcggcaagtcggtcggcacgcagtttgcgatcttcacctataaggatttcgatg<br>acgctgcgcgccgcgaagatctgatcaagatcgccatcgattgctgggcggaagtcgccgaacatgcgagccgtgccgg<br>tctcgactatgtgttctgggaaccgatgagcatcgggcgtgaatttggcgagacgattgccgaatgcatgaagcttcaggatc<br>gcctgaccgccgccgacatggctattccgatgtggatgatggccgatatcgaccacggcgatgtgacttccgccaatccgg<br>acgatttcgatccctacgcctgggcacgcgccgtgccgaaagtctcgccgatcattcacatcaagcagagcctgatggaca<br>agggcggacatcgtcctttcacagccgcgttcaatgccaagggacgcatccagccggaaccgcttctgaaagcctttgccg<br>aaggcggcgctgtggacaatgaaatctgcctcgaactgtcgttcaaggagcgcgagccgaatgaccgtgaagtcattccg<br>cagattgcggaaagtgtggctttctgggctccgcacattgacaccggcgctaaggacttgaagatataaacgatccatcgc<br>gatctggattcgctggaacataccggcgtgctgcgcaaagtacgcggcggcgcgacaatcgataccggcacacaatttga<br>aagcgatttccgcttccgcgaacggcaggacggcgaagcgaagcagaaaatggcccgcgccgcgcttgagcttgttgaa<br>cccggcatgacggtgatgattaatgacggctccatggcggctgtccttggcggcatgcttctggaaaagcgcccgctgaca<br>gtcatcaccaacaatgccgtcatcatcgatgaactgaagggcgagaacgggatcaatctgattgcgctcggcggaaccta<br>ttcagccaagttcaatgcgtttttcggcatcctgacggaaggggccctgtcgcatctgagcgccgacatcgccttcatttcctcg<br>cctgcagtcaatggcaagctcgtctatcacatggatgagaatgttgttcgcaccaagcgcgcgatgacggcatccgccgcc<br>cgcacctgccttctggtcaatcaccagcggttcggacgccctgccctgcatgtcatggctgatctttccgatttcgacgcgatc<br>ataaccgatgcctcccccgacgcgaccgtactggccgatcttgaacaggcgggcattgcactgaccatcgctgacgactg<br>cgcataaacctagaggacgaaaatcgagcatgacaaaattctggattggcaccagctggaagatgaacaagacgctgg<br>ccgaggcccgcattttcgccgaagccttgaaagctgccgatgcaagccgctcgaccgacattcagcgtttcgtcatcccgc<br>cctttaccgccgtgcgcgaagtgaaggaaatcctgttcggcacctccgtcaaggtcggcgcgcagaacatgcattgggcc<br>gatcagggagcatggaccggcgagatttcgccgctgatgctcaaggactgcaatctcgatatcgtcgaactcggtcattccg<br>agcgccgtgaacatttcggtgaaaccaacgaaacggtcggcctcaaggtcgaagctgcggtgcgccacggcctgatccc<br>actcatctgcatcggtgagacgctggaagaccgcgaaagcggacgcgccgcggaagttcttgaggaagaagtgcgcgg<br>tgcactttccaagctttccggtgaccagaagcaggcggaaatcctgtttgcctatgagccggtctgggccatcggcgaaaat<br>ggtatcccggcatcggcggaatatgccgatgcgcgccaggcggaaatcatcgcggttgcagaaagcgtactcggtcgtcg<br>tgtgccttgcctctatggcggctcggtcaatccgggcaattgcgaagagctgatcgcttgcccgcacattgacgggcttttcat<br>cggtcgctcggcatggaacgtcgaaggttatctcgacattctggccaagtgtgccgccaagactcgagctaattaaggagc<br>aatatgatgaaagtagcagtagcaggcgacagcgccggcgaaggtctggccaaggttctcgccgatcacctcaaggatc<br>gcttcgaggtttcggaaatctcgcgtacagacgctggcgcagatacattctacgccaacctctccgaccgcgtggcttccgc<br>cgttctcgacggcacctatgaccgcgccatcctcgtttgcggcaccggcatcggcgtatgcattgcagccaataaggttccg<br>ggcatccgcgccgcgctgacgcacgacacctattcggcagagcgcgcagcgctttccaacaatgcccagatcatcacca<br>tgggcgcccgcgtcatcggctcggaagtagccaagaccattgccgacgccttccttgctgagacctttgacgaaaacggcc<br>gttcggctggcaatgtcaacgcgatcaacgaagttgacgcgaagtacaacaagggctga	
programmed erythritol transport	including all erythritol transport-	ggcggctatgtgctttcattgacatttggattcctcccgcgaccgaaaatacgtcgctcgcagccctgtttataatgaaatcggg<br>cgatggcgtatgctggagataacataacgctatcacgttgtctgtaaaattttctagatggcgcatttttttcaccaaagagtga<br>atgtaggggaaatctccgcgccttcgagtgcggttttcccactgttttccatcaatagcttaatgtttcttcggttttaaatatgtttaa	This work

cluster	associated promoters, RBSs, CDSs (eryE/F/G) and terminators	ttcattgaaaataaaagaaaatatagaagtttgtgagtatctttgtggctggctttgcgagaaaagacggtgctgtgaatagtg<br>cgattgataacgaattatgtagtggatttctctagatcgtgatcgagattcggcgcgaaattgtgctatttcaccggtgtttcgaa<br>aacatccgattgacagctttcgagctttataacatcattattggagttaataacacgccgctatcataggttttgattgcggcgca<br>tctgtttccaggatgcacggaggagcggggccctggaattcatgtggaggctatcatgatctcgtctgtcgcgtcctgcaccg<br>ctttggcggaggcttttgcctatccggtaagcgtgatgacaatggtcgagggtaatctccgggcggtctaattcagccgcatc<br>atccgtttccataacgacaatacggcagaattgctgaaggcgtttgataatgagtgcacaaaccagtcaagtttcacaaaaa<br>cccgccaagctgagccgcgccgtcgtctggagcgtgatcgctgcgatcgtggttgtcggtgcgattgccttcgacacgaag<br>gtcgtgaagatcgggtctgattccgatgtgcgccagcaggctttctcccccgatgcctacggtgcttccgaattccccaaggt<br>gaaggcgagcgttgagcagcgcgccgtggatgcagtggaagttggaacggctctcgccgccgacaaggccgccgccg<br>gaaagaaatatggcgtcggcgacgtcaatccggttgtcccggtcaagtttacaggcaccgttgaggagcgcaaatccaac<br>tacaatgtcgtgaaggtcgacggcctgccggaaggcgttgccatccgcgttcagaccggccctgccgtcaacggcaccga<br>tctgcgcgatgcgaccggtgaaatccagttcggccagttcaagaaccagatcgaatatcaaaatgccggttctgccctgaa<br>caacgagatgaagaagcaagtgctttccggcgtcgatgtcgagaatctcgtcggcaagaccgtgacggtggttggcgtgtt<br>caaggtcgtcaatccgaagaactggctcgtcacgcctgtggggctcgaagtgaaatgagcaccgcttcgaaaatcgaag<br>gcaaaaacggtgatgtcgttcttgccgcaaacaacgttgccaaatcctatggtcgcattcatgccctgaagggcgtgaatttc<br>gagattcgccgtggtcaggtcacgacgcttttcggcgaaaacggcgctggcaaatcaacgttgatgaaagtgctttcgggc<br>gtcatccagccgacttcggggacgatcattctcgatggcgaacctgtgacgttcaattcgtccaccgaagcgcgcgacctcg<br>gcatctcgatcatccatcaggaattgagcctcgcgcccaacatgaacgtgcgcgacaacatcttcatggggcgcgaaatc<br>cgcaccgccaccggcgttgattttgccgaggaagaacgcctgacgcgcgcgcttctgaaggaactggaagaagacatcg<br>acccgctgacgccggtcgaggaacttcgtctcggccagcagcaggttgttgaaatcgcccgcgccctttcggtcaattcgcg<br>cattctcatcatggatgagccgacttcggcgctcagcgcctcggaagtggaagtcctgttcaaggtcattcgcgacctgacg<br>gcgcgcggtgtcgccatcgtctacatttcgcatcatctggaagaagcgttgcagatcaccaatcatgcggtggtgctgcgcg<br>acggaaccatgacggcctatgcgccgcgtgaggaaattgatctggaatggatcgtgcgcaacatggtcggcgagaacttc<br>gatctcggctcgcctccaaccggatatgactggggcgatgtggcgctctctgttgagaacctgaccgttcccgacccgggtg<br>gcgcgggcttctcgctggtcgaccgcatgtcgctcaatgtacgcgccggtgaaatcgtctgcatttatggtcttatgggcgccg<br>gtcgcacagaactgctggaaaccgttgccggtcgtctcaaggcaagcggcggacgtgttctcctcaaagggcaggacgttt<br>ccggcctcaccatcgcacaacgtatcgagaagggtcttgtgctggtgccggaagatcgccagcgcgacggtctcgtccag<br>acgatgacggtcggcaagaacctgtcgctcgccagcatcgccgaaatgaccaagggtctgttcacatcgcgcaagcgtg<br>aaaagcagattgtcgatcagtcgatcaagaatgtgcacatcaagacggatggcggcgaagcagcaatcggttcgctttcc<br>ggtggtaaccagcagaaggtcgtgatcggcaagatgctggcgaccgaaccggaagtcatcctgcttgatgagccgagcc<br>gcgggatcgacatcggggcgaaggcggaagtgttcaagcttctggctgaaaaggcgaagcagggtctggctgtcgtctac<br>acgacttcggaagtcggcgaatgcctcagcatcgctcatcgtatcatcgtcatgcaccgtggacgcatctctgccgaattcg<br>gatcggacgtgtccaaggagaagatcatggccgcctcgggcgaagccatggtcggtcactaaaacataatcccgaaaa<br>gttgcagacttttcggacaagattatgcgttaaaaacaaaaggatagagcataagacccgacaaagtcgggttgttaaatg<br>ctctggttatggagcactgatagaatgtcagtcacgagcaccaacaaagaaacagcggctccgaacggcgcgaagcgg<br>aaacccggcatcgtgcatctgctgctcgaaggtcgtgcatttttttgcactgatcgcgatcattgcagttttctcgttcctttcgcctt<br>attatttctcggtcgataacttcctcatcatgtcgtcgcacgtggccattttcggcctgcttgccatcggcatgctgctcgtcatcct<br>caatggcggcatcgatctttccgttggctccacattggggctggccggtgtggtcgccgggttcctgatgcagggcgtgacgc<br>tgcaggaattcggcatcattctctatttcccggtctgggccgtggttctcatcacctgtgcgctcggcgcattcgtcggcgcggtc<br>aatggtgtgctcatcgcctatctgcgcgttccggccttcgttgcgacgctcggcgttctctattgcgcgcgtggcgtggcgcttct<br>gatgaccaatggcctgacctacaacaatctcggcggacgtccggagcttggcaatacaggctttgactggctcggtttcaac<br>aagctgttcggcgtgccgatcggcgttctggttctggcggttctcgccatcatctgcgccatcgttctcaaccgcaccgcatttg<br>gtcgctggctctatgcgtcgggtggcaacgaacgtgcggctgaactgtccggcgttccggtcaagcgcgtcaaggtgtcggt<br>ctatgtcatttccggcatctgcgctgccattgcaggtctggttctttcttcgcagctgacgtcggccggtccgacggcaggcacc<br>acctacgaactgacggcaatcgctgccgtggttatcggtggcgctgcgctcactgggggcgcggtactatccagggcac<br>acttctcggcgctttcgtgatcggtttcctttccgacggcctcgtgattatcggcgtgtcctcctactggcagaccgtctttaccgg	

		cgcggtaatcgtgctcgcggttctgctcaacagcattcaatactcccggcgatga	

Supplementary Table 4. Vectors.

Vector name	Antibiotic resistance gene	Origin of replication	Reference
pSB1C3	CmR	ColE1	1, pSB1C3
pET22b	AmpR	ColE1+rop	This work
pFB1	AmpR	p15A	2
pKD4	AmpR	R6K	3

Vector pSB1C3 was obtained from "iGEM *Registry of Standard Biological Parts*" registry distribution kits (Spring 2016 Distribution and Spring 2017 Distribution).

Supplementary Table 5. Plasmids.

Plasmid name	Biobricks	Vector	Antibiotic resistance gene	Origin of replication
pFB147	pEryF-732 - eryA - eryB - eryC - eryH - eryI	pSB1C3	CmR	ColE1
pFB148	J23100 - eryA - eryB - eryC - eryH - eryI	pSB1C3	CmR	ColE1
pFB149	J23105 - eryA - eryB - eryC - eryH - eryI	pSB1C3	CmR	ColE1
pFB150	J23106 - eryA - eryB - eryC - eryH - eryI	pSB1C3	CmR	ColE1
pFB151	J23109 - eryA - eryB - eryC - eryH - eryI	pSB1C3	CmR	ColE1
pFB152	J23114 - eryA - eryB - eryC - eryH - eryI	pSB1C3	CmR	ColE1
pFB153	pEryF-732 - eryA - eryB - eryC - eryH	pSB1C3	CmR	ColE1
pFB154	pEryF-732 - eryA - eryB - eryC	pSB1C3	CmR	ColE1
pFB155	pEryF-732 - eryA - eryB	pSB1C3	CmR	ColE1
pFB156	pEryF-732 - eryA	pSB1C3	CmR	ColE1
pFB157	pEryR-732 - eryE - eryF - eryG	pFB1	AmpR	p15A
pFB158	araC - paraBAD - B0034 - (6xHis) - eryA - B0015	pSB1C3	CmR	ColE1
pFB159	araC - paraBAD - B0034 - (6xHis) - eryB - B0015	pSB1C3	CmR	ColE1
pFB160	araC - paraBAD - B0034 - (6xHis) - eryC - B0015	pSB1C3	CmR	ColE1
pFB161	pT7 - lacO - RBS - (6xHis) - eryD - T7 terminator	pET22b	AmpR	ColE1 + rop
pFB162	araC - paraBAD - B0034 - eryE - sfGFP - (6xHis) - B0015	pSB1C3	CmR	ColE1
pFB163	pT7 - lacO - RBS - (6xHis) - eryF - T7 terminator	pET22b	AmpR	ColE1 + rop
pFB164	araC - paraBAD - B0034 - eryG - sfGFP - (6xHis) - B0015	pSB1C3	CmR	ColE1
pFB165	pT7 - lacO - RBS - (6xHis) - eryH - T7 terminator	pET22b	AmpR	ColE1 + rop
pFB166	pT7 - lacO - RBS - (6xHis) - eryI - T7 terminator	pET22b	AmpR	ColE1 + rop
pFB167	pT7 - lacO - RBS - (6xHis) - eryR - T7 terminator	pET22b	AmpR	ColE1 + rop
pFB168	J23100 - eryA-RBS - sfGFP - (6xHis) - B0015	pSB1C3	CmR	ColE1
pFB169	J23100 - eryB-RBS - sfGFP - (6xHis) - B0015	pSB1C3	CmR	ColE1
pFB170	J23100 - eryC-RBS - sfGFP - (6xHis) - B0015	pSB1C3	CmR	ColE1
pFB171	J23100 - eryD-RBS - sfGFP - (6xHis) - B0015	pSB1C3	CmR	ColE1
pFB172	J23100 - eryE-RBS - sfGFP - (6xHis) - B0015	pSB1C3	CmR	ColE1

pFB173	J23100 - eryF-RBS - sfGFP - (6xHis) - B0015	pSB1C3	CmR	ColE1
pFB174	J23100 - eryG-RBS - sfGFP - (6xHis) - B0015	pSB1C3	CmR	ColE1
pFB175	J23100 - eryH-RBS - sfGFP - (6xHis) - B0015	pSB1C3	CmR	ColE1
pFB176	J23100 - eryI-RBS - sfGFP - (6xHis) - B0015	pSB1C3	CmR	ColE1
pFB177	pR-eryA - sfGFP - (6xHis) - B0015	pSB1C3	CmR	ColE1
pFB178	pR-eryB - sfGFP - (6xHis) - B0015	pSB1C3	CmR	ColE1
pFB179	pR-eryC - sfGFP - (6xHis) - B0015	pSB1C3	CmR	ColE1
pFB180	pR-eryD - sfGFP - (6xHis) - B0015	pSB1C3	CmR	ColE1
pFB181	pR-eryE - sfGFP - (6xHis) - B0015	pSB1C3	CmR	ColE1
pFB182	pR-eryF - sfGFP - (6xHis) - B0015	pSB1C3	CmR	ColE1
pFB183	pR-eryG - sfGFP - (6xHis) - B0015	pSB1C3	CmR	ColE1
pFB184	pR-eryH - sfGFP - (6xHis) - B0015	pSB1C3	CmR	ColE1
pFB185	pR-eryI - sfGFP - (6xHis) - B0015	pSB1C3	CmR	ColE1
pFB186	pEryF-732 - B0034 - sfGFP - (6xHis) - B0015	pSB1C3	CmR	ColE1
pFB187	pEryF-632 - B0034 - sfGFP - (6xHis) - B0015	pSB1C3	CmR	ColE1
pFB188	pEryF-532 - B0034 - sfGFP - (6xHis) - B0015	pSB1C3	CmR	ColE1
pFB189	pEryF-432 - B0034 - sfGFP - (6xHis) - B0015	pSB1C3	CmR	ColE1
pFB190	pEryF-332 - B0034 - sfGFP - (6xHis) - B0015	pSB1C3	CmR	ColE1
pFB191	pEryF-232 - B0034 - sfGFP - (6xHis) - B0015	pSB1C3	CmR	ColE1
pFB192	pEryF-222 - B0034 - sfGFP - (6xHis) - B0015	pSB1C3	CmR	ColE1
pFB193	pEryF-212 - B0034 - sfGFP - (6xHis) - B0015	pSB1C3	CmR	ColE1
pFB194	pEryF-202 - B0034 - sfGFP - (6xHis) - B0015	pSB1C3	CmR	ColE1
pFB195	pEryF-192 - B0034 - sfGFP - (6xHis) - B0015	pSB1C3	CmR	ColE1
pFB196	pEryF-182 - B0034 - sfGFP - (6xHis) - B0015	pSB1C3	CmR	ColE1
pFB197	pEryF-172 - B0034 - sfGFP - (6xHis) - B0015	pSB1C3	CmR	ColE1
pFB198	pEryF-162 - B0034 - sfGFP - (6xHis) - B0015	pSB1C3	CmR	ColE1
pFB199	pEryF-161 - B0034 - sfGFP - (6xHis) - B0015	pSB1C3	CmR	ColE1
pFB200	pEryF-160 - B0034 - sfGFP - (6xHis) - B0015	pSB1C3	CmR	ColE1
pFB201	pEryF-159 - B0034 - sfGFP - (6xHis) - B0015	pSB1C3	CmR	ColE1
pFB202	pEryF-158 - B0034 - sfGFP - (6xHis) - B0015	pSB1C3	CmR	ColE1
pFB203	pEryF-157 - B0034 - sfGFP - (6xHis) - B0015	pSB1C3	CmR	ColE1
pFB204	pEryF-156 - B0034 - sfGFP - (6xHis) - B0015	pSB1C3	CmR	ColE1
pFB205	pEryF-155 - B0034 - sfGFP - (6xHis) - B0015	pSB1C3	CmR	ColE1
pFB206	pEryF-154 - B0034 - sfGFP - (6xHis) - B0015	pSB1C3	CmR	ColE1
pFB207	pEryF-153 - B0034 - sfGFP - (6xHis) - B0015	pSB1C3	CmR	ColE1
pFB208	pEryF-152 - B0034 - sfGFP - (6xHis) - B0015	pSB1C3	CmR	ColE1
pFB209	pEryF-142 - B0034 - sfGFP - (6xHis) - B0015	pSB1C3	CmR	ColE1
pFB210	pEryF-132 - B0034 - sfGFP - (6xHis) - B0015	pSB1C3	CmR	ColE1
pFB211	pEryF-122 - B0034 - sfGFP - (6xHis) - B0015	pSB1C3	CmR	ColE1
pFB212	pEryF-112 - B0034 - sfGFP - (6xHis) - B0015	pSB1C3	CmR	ColE1
pFB213	pEryF-102 - B0034 - sfGFP - (6xHis) - B0015	pSB1C3	CmR	ColE1
pFB214	pEryF-159-10 - B0034 - sfGFP - (6xHis) - B0015	pSB1C3	CmR	ColE1
pFB215	pEryF-159-20 - B0034 - sfGFP - (6xHis) - B0015	pSB1C3	CmR	ColE1
pFB216	pEryF-159-30 - B0034 - sfGFP - (6xHis) - B0015	pSB1C3	CmR	ColE1

pFB217	pEryF-159-40 - B0034 - sfGFP - (6xHis) - B0015	pSB1C3	CmR	ColE1
pFB218	pEryF-159-50 - B0034 - sfGFP - (6xHis) - B0015	pSB1C3	CmR	ColE1
pFB219	pEryF-159-60 - B0034 - sfGFP - (6xHis) - B0015	pSB1C3	CmR	ColE1
pFB220	pEryF-159-70 - B0034 - sfGFP - (6xHis) - B0015	pSB1C3	CmR	ColE1
pFB221	pEryF-159-80 - B0034 - sfGFP - (6xHis) - B0015	pSB1C3	CmR	ColE1
pFB222	pEryF-159-90 - B0034 - sfGFP - (6xHis) - B0015	pSB1C3	CmR	ColE1
pFB223	pEryF-159-100 - B0034 - sfGFP - (6xHis) - B0015	pSB1C3	CmR	ColE1
pFB224	pEryF-159-110 - B0034 - sfGFP - (6xHis) - B0015	pSB1C3	CmR	ColE1
pFB225	pEryF-159-120 - B0034 - sfGFP - (6xHis) - B0015	pSB1C3	CmR	ColE1
pFB226	pEryF-159-130 - B0034 - sfGFP - (6xHis) - B0015	pSB1C3	CmR	ColE1
pFB227	pEryF-159-140 - B0034 - sfGFP - (6xHis) - B0015	pSB1C3	CmR	ColE1
pFB228	pEryF-159-150 - B0034 - sfGFP - (6xHis) - B0015	pSB1C3	CmR	ColE1
pFB229	pEryF-37 - B0034 - sfGFP - (6xHis) - B0015	pSB1C3	CmR	ColE1
pFB230	pEryF-35 - B0034 - sfGFP - (6xHis) - B0015	pSB1C3	CmR	ColE1
pFB231	pEryF-33 - B0034 - sfGFP - (6xHis) - B0015	pSB1C3	CmR	ColE1
pFB232	pEryF-31 - B0034 - sfGFP - (6xHis) - B0015	pSB1C3	CmR	ColE1
pFB233	pEryF-29 - B0034 - sfGFP - (6xHis) - B0015	pSB1C3	CmR	ColE1
pFB234	pEryF-28 - B0034 - sfGFP - (6xHis) - B0015	pSB1C3	CmR	ColE1
pFB235	pEryF-27 - B0034 - sfGFP - (6xHis) - B0015	pSB1C3	CmR	ColE1
pFB236	pEryF-25 - B0034 - sfGFP - (6xHis) - B0015	pSB1C3	CmR	ColE1
pFB237	pEryF-23 - B0034 - sfGFP - (6xHis) - B0015	pSB1C3	CmR	ColE1
pFB238	pEryF-21 - B0034 - sfGFP - (6xHis) - B0015	pSB1C3	CmR	ColE1
pFB239	pEryR-732 - B0034 - sfGFP - (6xHis) - B0015	pSB1C3	CmR	ColE1
pFB240	pEryR-632 - B0034 - sfGFP - (6xHis) - B0015	pSB1C3	CmR	ColE1
pFB241	pEryR-532 - B0034 - sfGFP - (6xHis) - B0015	pSB1C3	CmR	ColE1
pFB242	pEryR-432 - B0034 - sfGFP - (6xHis) - B0015	pSB1C3	CmR	ColE1
pFB243	pEryR-332 - B0034 - sfGFP - (6xHis) - B0015	pSB1C3	CmR	ColE1
pFB244	pEryR-322 - B0034 - sfGFP - (6xHis) - B0015	pSB1C3	CmR	ColE1
pFB245	pEryR-312 - B0034 - sfGFP - (6xHis) - B0015	pSB1C3	CmR	ColE1
pFB246	pEryR-302 - B0034 - sfGFP - (6xHis) - B0015	pSB1C3	CmR	ColE1
pFB247	pEryR-292 - B0034 - sfGFP - (6xHis) - B0015	pSB1C3	CmR	ColE1
pFB248	pEryR-282 - B0034 - sfGFP - (6xHis) - B0015	pSB1C3	CmR	ColE1
pFB249	pEryR-272 - B0034 - sfGFP - (6xHis) - B0015	pSB1C3	CmR	ColE1
pFB250	pEryR-262 - B0034 - sfGFP - (6xHis) - B0015	pSB1C3	CmR	ColE1
pFB251	pEryR-252 - B0034 - sfGFP - (6xHis) - B0015	pSB1C3	CmR	ColE1
pFB252	pEryR-242 - B0034 - sfGFP - (6xHis) - B0015	pSB1C3	CmR	ColE1
pFB253	pEryR-232 - B0034 - sfGFP - (6xHis) - B0015	pSB1C3	CmR	ColE1
pFB254	pEryR-132 - B0034 - sfGFP - (6xHis) - B0015	pSB1C3	CmR	ColE1
pFB255	pEryR-32 - B0034 - sfGFP - (6xHis) - B0015	pSB1C3	CmR	ColE1
pFB256	pEry2-400 - B0034 - sfGFP - (6xHis) - B0015	pSB1C3	CmR	ColE1
pFB257	pEry2-300 - B0034 - sfGFP - (6xHis) - B0015	pSB1C3	CmR	ColE1
pFB258	pEry2-200 - B0034 - sfGFP - (6xHis) - B0015	pSB1C3	CmR	ColE1
pFB259	pEry2-100 - B0034 - sfGFP - (6xHis) - B0015	pSB1C3	CmR	ColE1
pFB260	J23105 - B0034 - eryD	pFB1	AmpR	p15A

pFB261	J23106 - B0034 - eryD	pFB1	AmpR	p15A
pFB262	J23109 - B0034 - eryD	pFB1	AmpR	p15A
pFB263	J23113 - B0034 - eryD	pFB1	AmpR	p15A
pFB264	J23114 - B0034 - eryD	pFB1	AmpR	p15A
pFB265	J23105 - B0034 - eryR	pFB1	AmpR	p15A
pFB266	J23106 - B0034 - eryR	pFB1	AmpR	p15A
pFB267	J23109 - B0034 - eryR	pFB1	AmpR	p15A
pFB268	J23113 - B0034 - eryR	pFB1	AmpR	p15A
pFB269	J23114 - B0034 - eryR	pFB1	AmpR	p15A
pFB270	J23100 - eryO - B0034 - sfGFP - (6xHis) - B0015	pSB1C3	CmR	ColE1
pFB271	J23105 - eryO - B0034 - sfGFP - (6xHis) - B0015	pSB1C3	CmR	ColE1
pFB272	J23106 - eryO - B0034 - sfGFP - (6xHis) - B0015	pSB1C3	CmR	ColE1
pFB273	J23109 - eryO - B0034 - sfGFP - (6xHis) - B0015	pSB1C3	CmR	ColE1
pFB274	J23113 - eryO - B0034 - sfGFP - (6xHis) - B0015	pSB1C3	CmR	ColE1
pFB275	J23114 - eryO - B0034 - sfGFP - (6xHis) - B0015	pSB1C3	CmR	ColE1
pFB276	pT7 - eryO - RBS - sfGFP - (6xHis) - B0015	pSB1C3	CmR	ColE1
pFB277	pT7 - RBS - sfGFP - (6xHis) - B0015	pSB1C3	CmR	ColE1
pFB278	alsC-homoL - FRT - KanR - FRT - alsC-homoR	pKD4	AmpR / KanR	R6K
pFB279	araH-homoL - FRT - KanR - FRT - araH-homoR	pKD4	AmpR / KanR	R6K
pFB280	glpF-homoL - FRT - KanR - FRT - glpF-homoR	pKD4	AmpR / KanR	R6K
pFB281	malG-homoL - FRT - KanR - FRT - malG-homoR	pKD4	AmpR / KanR	R6K
pFB282	mglC-homoL - FRT - KanR - FRT - mglC-homoR	pKD4	AmpR / KanR	R6K
pFB283	rbsC-homoL - FRT - KanR - FRT - rbsC-homoR	pKD4	AmpR / KanR	R6K
pFB284	xylH-homoL - FRT - KanR - FRT - xylH-homoR	pKD4	AmpR / KanR	R6K
pFB285	J23100 - B0034 - sfGFP - (6xHis) - B0015	pFB1	AmpR	p15A
pFB286	J23100 - B0034 - sfGFP - (6xHis) - B0015	pSB1C3	CmR	ColE1
pFB287	J23105 - B0034 - sfGFP - (6xHis) - B0015	pSB1C3	CmR	ColE1
pFB288	J23106 - B0034 - sfGFP - (6xHis) - B0015	pSB1C3	CmR	ColE1
pFB289	J23109 - B0034 - sfGFP - (6xHis) - B0015	pSB1C3	CmR	ColE1
pFB290	J23113 - B0034 - sfGFP - (6xHis) - B0015	pSB1C3	CmR	ColE1
pFB291	J23114 - B0034 - sfGFP - (6xHis) - B0015	pSB1C3	CmR	ColE1
pFB292	pEryF-32 - B0034 - sfGFP - (6xHis) - B0015	pSB1C3	CmR	ColE1
pFB293	J23105 - B0034 - talA - tktB	pFB1	AmpR	p15A
pFB294	J23106 - B0034 - talA - tktB	pFB1	AmpR	p15A
pFB295	J23109 - B0034 - talA - tktB	pFB1	AmpR	p15A
pFB296	J23113 - B0034 - talA - tktB	pFB1	AmpR	p15A
pFB297	J23114 - B0034 - talA - tktB	pFB1	AmpR	p15A
pFB298	J23105 - B0034 - talB	pFB1	AmpR	p15A
pFB299	J23106 - B0034 - talB	pFB1	AmpR	p15A
pFB300	J23113 - B0034 - talB	pFB1	AmpR	p15A
pFB301	J23114 - B0034 - talB	pFB1	AmpR	p15A
pFB302	J23105 - B0034 - tktA	pFB1	AmpR	p15A
pFB303	J23106 - B0034 - tktA	pFB1	AmpR	p15A
pFB304	J23113 - B0034 - tktA	pFB1	AmpR	p15A

pFB305	J23114 - B0034 - tktA	pFB1	AmpR	p15A
pFB306	yiaMNO-homoL - FRT - KanR - FRT - yiaMNO-homoR	pKD4	AmpR / KanR	R6K
pFB307	fryBCA-homoL - FRT - KanR - FRT - fryBCA-homoR	pKD4	AmpR / KanR	R6K
pFB308	garP-homoL - FRT - KanR - FRT - garP-homoR	pKD4	AmpR / KanR	R6K
pFB309	gntT-homoL - FRT - KanR - FRT - gntT-homoR	pKD4	AmpR / KanR	R6K
pFB310	ulaABC-homoL - FRT - KanR - FRT - ulaABC-homoR	pKD4	AmpR / KanR	R6K
pFB311	agaBCD-homoL - FRT - KanR - FRT - agaBCD-homoR	pKD4	AmpR / KanR	R6K
pFB312	agaVW-homoL - FRT - KanR - FRT - agaVW-homoR	pKD4	AmpR / KanR	R6K
pFB313	ydjK-homoL - FRT - KanR - FRT - ydjK-homoR	pKD4	AmpR / KanR	R6K
pFB314	yjiJ-homoL - FRT - KanR - FRT - yjiJ-homoR	pKD4	AmpR / KanR	R6K
pFB315	yagG-homoL - FRT - KanR - FRT - yagG-homoR	pKD4	AmpR / KanR	R6K
pFB316	yaaU-homoL - FRT - KanR - FRT - yaaU-homoR	pKD4	AmpR / KanR	R6K
pFB317	yicJ-homoL - FRT - KanR - FRT - yicJ-homoR	pKD4	AmpR / KanR	R6K
pFB318	frvAB-homoL - FRT - KanR - FRT - frvAB-homoR	pKD4	AmpR / KanR	R6K
pFB319	frwCB-homoL - FRT - KanR - FRT - frwCB-homoR	pKD4	AmpR / KanR	R6K
pFB320	nanT-homoL - FRT - KanR - FRT - nanT-homoR	pKD4	AmpR / KanR	R6K
pFB321	yfaV-homoL - FRT - KanR - FRT - yfaV-homoR	pKD4	AmpR / KanR	R6K
pFB322	sgcBCA-homoL - FRT - KanR - FRT - sgcBCA-homoR	pKD4	AmpR / KanR	R6K
pFB323	dgoT-homoL - FRT - KanR - FRT - dgoT-homoR	pKD4	AmpR / KanR	R6K
pFB324	mngA-homoL - FRT - KanR - FRT - mngA-homoR	pKD4	AmpR / KanR	R6K
pFB325	ugpBAEC-homoL - FRT - KanR - FRT - ugpBAEC-homoR	pKD4	AmpR / KanR	R6K
pFB326	ycjNOP-homoL - FRT - KanR - FRT - ycjNOP-homoR	pKD4	AmpR / KanR	R6K
pFB327	uhpT-homoL - FRT - KanR - FRT - uhpT-homoR	pKD4	AmpR / KanR	R6K
pFB328	ydjE-homoL - FRT - KanR - FRT - ydjE-homoR	pKD4	AmpR / KanR	R6K
pFB329	yphFED-homoL - FRT - KanR - FRT - yphFED-homoR	pKD4	AmpR / KanR	R6K
pFB330	yhjDE-homoL - FRT - KanR - FRT - yhjDE-homoR	pKD4	AmpR / KanR	R6K
pFB331	ytfT-homoL - FRT - KanR - FRT - ytfT-homoR	pKD4	AmpR / KanR	R6K
pFB332	srlAEB-homoL - FRT - KanR - FRT - srlAEB-homoR	pKD4	AmpR / KanR	R6K
pFB333	yfcJ-homoL - FRT - KanR - FRT - yfcJ-homoR	pKD4	AmpR / KanR	R6K
pFB334	lsrACDB-homoL - FRT - KanR - FRT - lsrACDB-homoR	pKD4	AmpR / KanR	R6K
pFB335	melB-homoL - FRT - KanR - FRT - melB-homoR	pKD4	AmpR / KanR	R6K
pFB336	mhpT-homoL - FRT - KanR - FRT - mhpT-homoR	pKD4	AmpR / KanR	R6K
pFB337	yjhF-homoL - FRT - KanR - FRT - yjhF-homoR	pKD4	AmpR / KanR	R6K
pFB338	glvCB-homoL - FRT - KanR - FRT - glvCB-homoR	pKD4	AmpR / KanR	R6K
pFB339	yidK-homoL - FRT - KanR - FRT - yidK-homoR	pKD4	AmpR / KanR	R6K
pFB340	glcC - pglcD - B0034 - sfGFP - (6xHis) - B0015	pFB1	AmpR	p15A

Unless otherwise mentioned, all plasmids above were constructed by Gibson Assembly and conducted in the strain Mach1-T1. All plasmids with R6K origin of replication were conducted in the strain DH5α λpir.

All constructed plasmids were sequenced correctly.

All “6xHis” represents a “six-repeat-histidine” amino acid sequence located in *N*-terminus or *C*-terminus of each protein.

Supplementary Table 6. Primers.

Primer name	Sequence (5' to 3')	Purpose	Reference
VF2	tgccacctgacgtctaagaa	pSB1C3 and pFB1 derived plasmids sequencing, colony PCR	1
VR	attaccgcctttgagtgagc	pSB1C3 and pFB1 derived plasmids sequencing, colony PCR	1
27F	agagtttgatcctggctcag	Prokaryotic organism 16s rRNA sequence amplification	This work
1492R	ggttaccttgttacgactt	Prokaryotic organism 16s rRNA sequence amplification	This work

All primers above were synthesized by GENEWIZ.

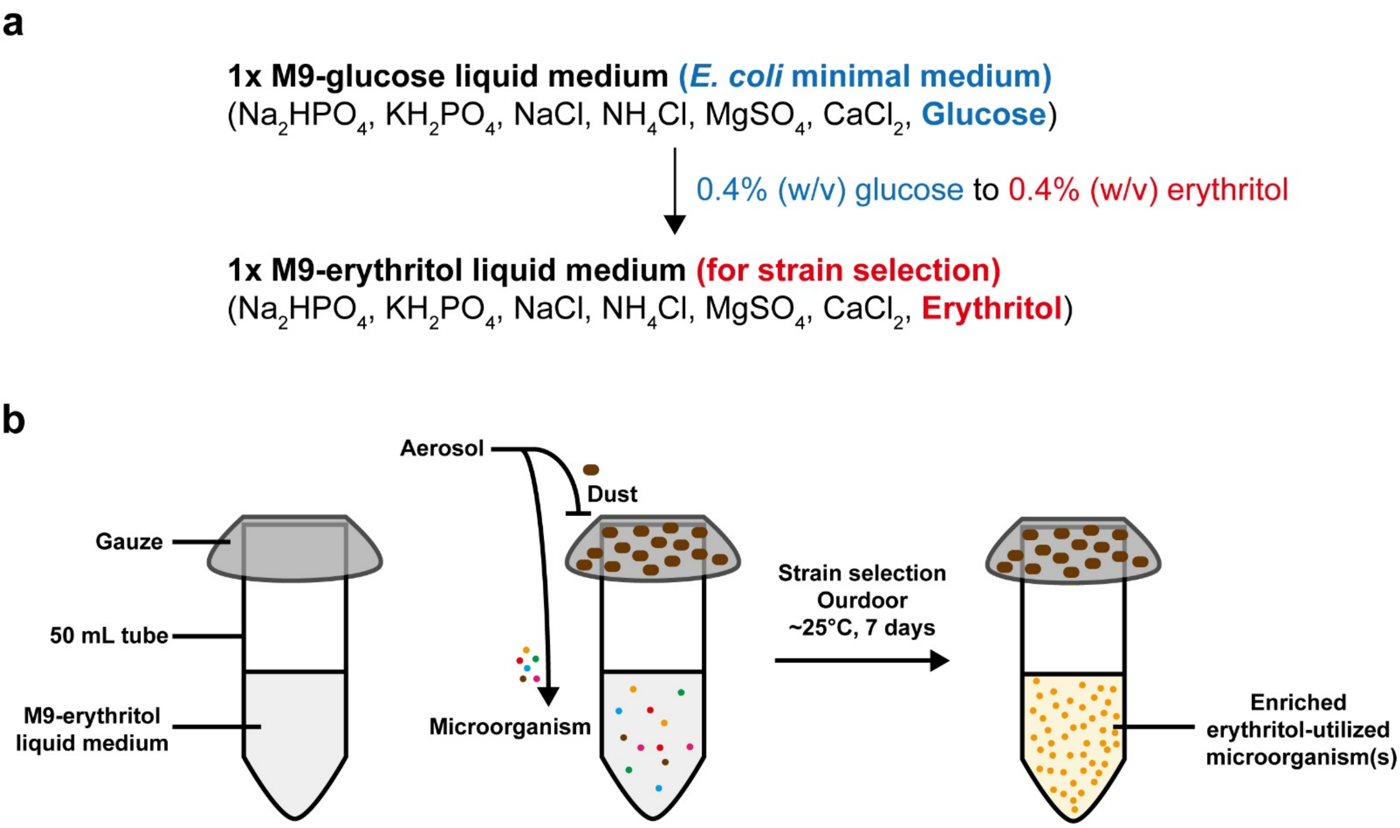

Supplementary Figure 1. Isolation of microorganisms growing in the erythritol-based M9 medium.

(**a**) 1x M9-erythritol liquid medium is derived from 1x M9-glucose liquid medium by changing 0.4% (w/v) glucose into 0.4% (w/v) erythritol and other components are the same. 1 L 1x M9-erythritol liquid medium consists of 12.8 g $Na_2HPO_4 \cdot 7H_2O$, 3 g KH_2PO_4, 0.5 g NaCl, 1 g NH_4Cl, 200 μL 1 M $MgSO_4$, 10 μL 1 M $CaCl_2$, and 4 g erythritol.

(**b**) Schematic of screening erythritol-utilized microorganisms. A 50 mL centrifuge tube (without lid) contains approximately 25 mL 1x M9-erythritol liquid medium. The covered gauze can block aerosol dust, but aerosol microorganisms can fall into the liquid medium. After 7 days of outdoor stationary culture (approximately 25°C), the microorganisms which can utilize erythritol as sole carbon source were screened out and enriched, then the liquid culture was separated on LB-agar plate for picking up single colony.

a

16s rRNA BLAST alignments (primer pair: 27F / 1492R)

Strain	Query coverage	Percent identity	Accession
Ochrobactrum sp. strain SNH-K10	100%	100%	MN493878.1
Ochrobactrum anthropi strain SCA-1	100%	100%	MH448104.1
Ochrobactrum sp. TE-101	100%	100%	KC998944.1
Ochrobactrum sp. C14	100%	100%	KT361086.1
Ochrobactrum anthropi strain LSP34	100%	100%	MW485664.1
Ochrobactrum sp. strain LSC9	100%	100%	MW485524.1
Ochrobactrum anthropi strain DP5	100%	99.92%	MT534544.1
Ochrobactrum sp. strain JWDC2	100%	99.92%	MT448953.1
Ochrobactrum sp. strain JWJC3	100%	99.92%	MT448947.1
Ochrobactrum anthropi strain L1-W	100%	99.92%	MT093466.1
......	......	......	......

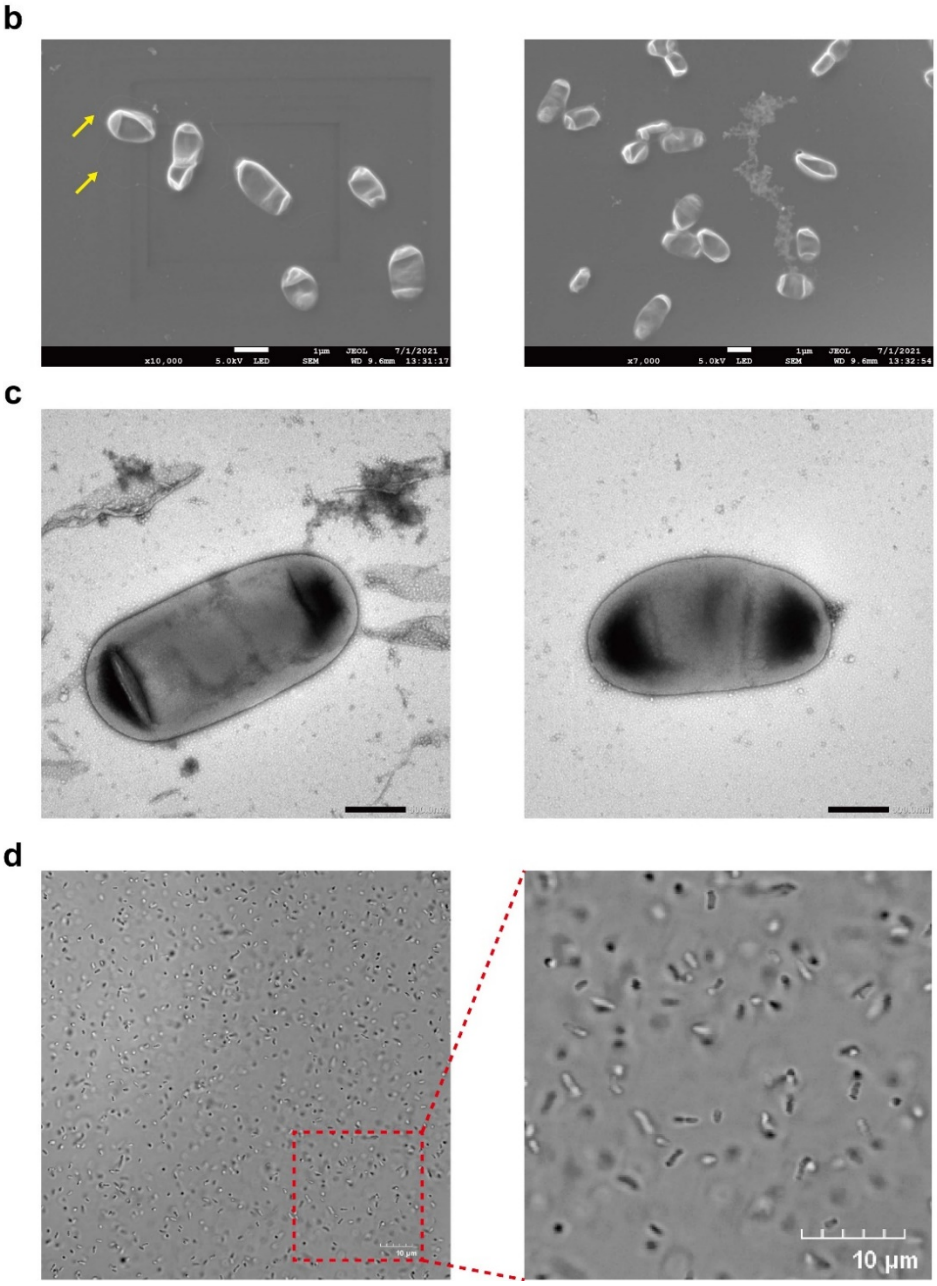

Supplementary Figure 2. Characterization of the isolated *Ochrobactrum* spp. strain.

(**a**) BLAST[4,5] alignments of screened-out *Ochrobactrum* spp. 16s rRNA sequence.

(**b**) Scanning electron microscopy images of *Ochrobactrum* spp.. Yellow arrows indicate peritrichous flagella.

(**c**) Transmission electron microscopy images of *Ochrobactrum* spp..

(**d**) Confocal laser scanning microscopy images of *Ochrobactrum* spp..

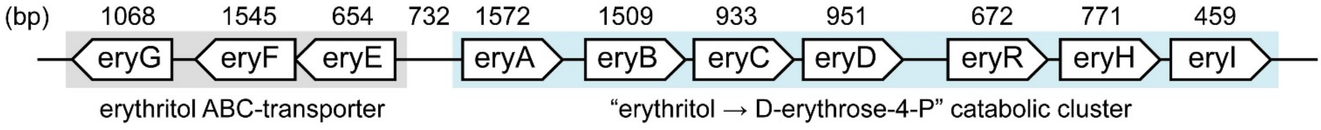

eryE: DUF (Domain of Unknown Function) 2291 family protein (peripheral erythritol-binding protein)
eryF: Sugar ABC transporter ATP-binding protein (erythritol ABC transporter ATP-binding protein)
eryG: ABC transporter permease (erythritol ABC transporter permease)

eryA: Carbohydrate kinase (erythritol kinase)
eryB: Glycerol-3-phosphate dehydrogenase (erythritol-4-phosphate dehydrogenase)
eryC: TIM barrel protein (L-3-tetrulose-4-phosphate to D-3-tetrulose-4-phosphate isomerase)
eryD: Sugar-binding transcriptional regulator (erythritol-binding transcriptional regulator)
eryR: DeoR/GlpR family DNA-binding transcription regulator (Unknown)
eryH: Triose-phosphate isomerase (D-3-tetrulose-4-phosphate to D-erythrulose-4-phosphate isomerase)
eryI: RpiB/LacA/LacB family sugar-phosphate isomerase
(D-erythrulose-4-phosphate to D-erythrose-4-phosphate isomerase)

Supplementary Figure 3. Annotation of erythritol catabolism-associated genes.
Schematic of erythritol catabolism-associated cluster is shown with ten different genes. Black annotations represent "protein BLAST alignment" results of each gene, red annotations represent each gene's detailed function.

eryA nucleotide BLAST alignments

Strain	Query coverage	Percent identity	Accession
Brucella anthropi strain T16R-87	100%	99.81%	CP044971.1
Ochrobactrum sp. MT180101	100%	98.28%	CP061773.1
Ochrobactrum anthropi	100%	98.03%	LT671862.1
Brucella anthropi strain PBO	100%	98.03%	CP064062.1
Ochrobactrum anthropi strain OAB	100%	97.96%	CP008819.1
Brucella anthropi strain FDAARGOS_1039	100%	97.96%	CP066053.1
Ochrobactrum anthropi ATCC 49188	100%	97.96%	CP000759.1
Ochrobactrum sp. WY7	100%	97.77%	CP049797.1
Ochrobactrum sp. EEELCW01	100%	97.65%	CP047599.1
Brucella intermedia strain ZJ499	98%	86.87%	CP061040.1

eryB nucleotide BLAST alignments

Strain	Query coverage	Percent identity	Accession
Brucella anthropi strain T16R-87	100%	99.93%	CP044971.1
Ochrobactrum sp. EEELCW01	100%	98.54%	CP047599.1
Ochrobactrum sp. MT180101	100%	98.54%	CP061773.1
Ochrobactrum anthropi	100%	98.41%	LT671862.1
Brucella anthropi strain PBO	100%	98.34%	CP064062.1
Ochrobactrum anthropi strain OAB	100%	96.95%	CP008819.1
Brucella anthropi strain FDAARGOS_1039	100%	96.95%	CP066053.1
Ochrobactrum anthropi ATCC 49188	100%	96.95%	CP000759.1
Ochrobactrum sp. WY7	100%	95.96%	CP049797.1
Brucella intermedia strain ZJ499	100%	92.58%	CP061040.1

eryC nucleotide BLAST alignments

Strain	Query coverage	Percent identity	Accession
Brucella anthropi strain T16R-87	100%	100.00%	CP044971.1
Ochrobactrum anthropi	100%	99.25%	LT671862.1
Brucella anthropi strain PBO	100%	99.25%	CP064062.1
Ochrobactrum sp. MT180101	100%	98.50%	CP061773.1
Ochrobactrum sp. EEELCW01	99%	97.53%	CP047599.1
Ochrobactrum anthropi strain OAB	100%	97.21%	CP008819.1
Brucella anthropi strain FDAARGOS_1039	100%	97.21%	CP066053.1
Ochrobactrum anthropi ATCC 49188	100%	97.21%	CP000759.1
Ochrobactrum sp. WY7	100%	97.11%	CP049797.1
Brucella sp. 09RB8471	99%	90.57%	CP019346.1

eryD nucleotide BLAST alignments

Strain	Query coverage	Percent identity	Accession
Brucella anthropi strain T16R-87	100%	100.00%	CP044971.1
Ochrobactrum anthropi	100%	97.58%	LT671862.1
Brucella anthropi strain PBO	100%	97.48%	CP064062.1
Ochrobactrum anthropi strain OAB	100%	97.16%	CP008819.1
Brucella anthropi strain FDAARGOS_1039	100%	97.16%	CP066053.1
Ochrobactrum anthropi ATCC 49188	100%	97.16%	CP000759.1
Ochrobactrum sp. WY7	100%	96.85%	CP049797.1
Ochrobactrum sp. EEELCW01	100%	96.42%	CP047599.1
Ochrobactrum sp. MT180101	100%	96.42%	CP061773.1
Brucella intermedia strain ZJ499	96%	87.92%	CP061040.1

eryE nucleotide BLAST alignments

Strain	Query coverage	Percent identity	Accession
Brucella anthropi strain T16R-87	100%	100.00%	CP044971.1
Ochrobactrum sp. MT180101	100%	100.00%	CP061773.1
Ochrobactrum sp. WY7	100%	98.78%	CP049797.1
Ochrobactrum anthropi strain OAB	100%	98.62%	CP008819.1
Brucella anthropi strain FDAARGOS_1039	100%	98.62%	CP066053.1
Ochrobactrum anthropi ATCC 49188	100%	98.62%	CP000759.1
Brucella anthropi strain PBO	100%	98.01%	CP064062.1
Ochrobactrum anthropi	100%	97.71%	LT671862.1
Ochrobactrum sp. EEELCW01	100%	96.94%	CP047599.1
Brucella intermedia strain ZJ499	100%	90.38%	CP061040.1

eryF nucleotide BLAST alignments

Strain	Query coverage	Percent identity	Accession
Brucella anthropi strain T16R-87	100%	99.87%	CP044971.1
Ochrobactrum anthropi	100%	98.77%	LT671862.1
Ochrobactrum sp. MT180101	100%	98.77%	CP061773.1
Ochrobactrum anthropi strain OAB	100%	98.45%	CP008819.1
Brucella anthropi strain FDAARGOS_1039	100%	98.45%	CP066053.1
Brucella anthropi strain PBO chromosome 1	100%	98.45%	CP064062.1
Ochrobactrum anthropi ATCC 49188	100%	98.45%	CP000759.1
Ochrobactrum sp. EEELCW01	100%	98.38%	CP047599.1
Ochrobactrum sp. WY7	100%	98.25%	CP049797.1
Brucella intermedia strain ZJ499	99%	93.13%	CP061040.1

eryG nucleotide BLAST alignments

Strain	Query coverage	Percent identity	Accession
Ochrobactrum sp. MT180101	100%	100.00%	CP061773.1
Brucella anthropi strain T16R-87	100%	99.91%	CP044971.1
Brucella anthropi strain PBO	100%	98.78%	CP064062.1
Ochrobactrum anthropi	100%	98.13%	LT671862.1
Ochrobactrum anthropi strain OAB	100%	98.13%	CP008819.1
Ochrobactrum sp. WY7	100%	98.13%	CP049797.1
Brucella anthropi strain FDAARGOS_1039	100%	98.13%	CP066053.1
Ochrobactrum anthropi ATCC 49188	100%	98.13%	CP000759.1
Ochrobactrum sp. EEELCW01	100%	97.85%	CP047599.1
Brucella intermedia strain ZJ499	99%	91.68%	CP061040.1

eryH nucleotide BLAST alignments

Strain	Query coverage	Percent identity	Accession
Brucella anthropi strain T16R-87	100%	100.00%	CP044971.1
Ochrobactrum anthropi	100%	100.00%	LT671862.1
Ochrobactrum sp. EEELCW01	100%	99.22%	CP047599.1
Ochrobactrum sp. MT180101	100%	97.67%	CP061773.1
Ochrobactrum anthropi strain OAB	100%	96.89%	CP008819.1
Ochrobactrum sp. WY7	100%	96.89%	CP049797.1
Brucella anthropi strain FDAARGOS_1039	100%	96.89%	CP066053.1
Ochrobactrum anthropi ATCC 49188	100%	96.89%	CP000759.1
Brucella anthropi strain PBO	100%	96.24%	CP064062.1
Brucella sp. BO3	100%	87.81%	CP047233.1

eryI nucleotide BLAST alignments

Strain	Query coverage	Percent identity	Accession
Brucella anthropi strain T16R-87	100%	100.00%	CP044971.1
Ochrobactrum anthropi genome assembly	100%	100.00%	LT671862.1
Ochrobactrum sp. MT180101	100%	99.56%	CP061773.1
Ochrobactrum sp. WY7	100%	99.13%	CP049797.1
Ochrobactrum anthropi strain OAB	100%	98.91%	CP008819.1
Brucella anthropi strain FDAARGOS_1039	100%	98.91%	CP066053.1
Brucella anthropi strain PBO chromosome 1	100%	98.91%	CP064062.1
Ochrobactrum anthropi ATCC 49188	100%	98.91%	CP000759.1
Ochrobactrum sp. EEELCW01	100%	97.39%	CP047599.1
Ochrobactrum sp. PW1	99%	93.86%	LC171366.1

eryR nucleotide BLAST alignments

Strain	Query coverage	Percent identity	Accession
Brucella anthropi strain T16R-87	100%	100.00%	CP044971.1
Ochrobactrum anthropi	100%	100.00%	LT671862.1
Ochrobactrum sp. EEELCW01	100%	100.00%	CP047599.1
Brucella anthropi strain PBO	99%	98.66%	CP064062.1
Ochrobactrum sp. MT180101	100%	98.51%	CP061773.1
Ochrobactrum anthropi strain OAB	100%	98.36%	CP008819.1
Ochrobactrum sp. WY7	100%	98.36%	CP049797.1
Brucella anthropi strain FDAARGOS_1039	100%	98.36%	CP066053.1
Ochrobactrum anthropi ATCC 49188	100%	98.36%	CP000759.1
Ochrobactrum sp. PW1	94%	88.89%	LC171366.1

Supplementary Figure 4. Nucleotide BLAST alignments of erythritol catabolism-associated genes.
Top ten nucleotide alignments of every erythritol catabolism-associated genes (eryA/B/C/D/E/F/G/H/I/R) in *Ochrobactrum* spp..

eryA protein BLAST alignments

Strain	Query coverage	Percent identity	Accession
Brucella anthropi	99%	99.81%	WP_151615846.1
Brucella/Ochrobactrum group	99%	99.62%	WP_036587141.1
Brucella anthropi	99%	99.43%	WP_011982654.1
Ochrobactrum sp. UNC390CL2Tsu3S39	99%	99.24%	WP_029928109.1
Brucella/Ochrobactrum group	99%	99.24%	WP_209418660.1
Brucella anthropi	99%	99.04%	WP_151607003.1
Brucella anthropi	99%	98.85%	WP_151612751.1
Brucella/Ochrobactrum group	99%	99.04%	WP_094515042.1
Brucella/Ochrobactrum group	99%	99.04%	WP_010660871.1
Brucella tritici	99%	97.90%	WP_151557001.1

eryB protein BLAST alignments

Strain	Query coverage	Percent identity	Accession
Brucella anthropi	99%	99.80%	WP_036587140.1
Brucella/Ochrobactrum group	99%	100.00%	WP_043061612.1
Brucella anthropi	99%	99.60%	WP_151663531.1
Brucella/Ochrobactrum group	99%	99.60%	WP_094515041.1
Brucella anthropi	99%	99.40%	MBE0559574.1
Ochrobactrum sp. EEELCW01	99%	99.40%	WP_209418661.1
Brucella anthropi	99%	99.20%	WP_011982653.1
Ochrobactrum sp. UNC390CL2Tsu3S39	99%	99.20%	WP_029928112.1
Brucella anthropi	99%	99.40%	WP_151614594.1
Brucella anthropi	99%	99.00%	WP_061346705.1

eryC protein BLAST alignments

Strain	Query coverage	Percent identity	Accession
Brucella/Ochrobactrum group	99%	100.00%	WP_036587138.1
Brucella/Ochrobactrum group	99%	99.68%	WP_010660869.1
Brucella anthropi	99%	99.35%	WP_213506816.1
Brucella anthropi	99%	99.35%	WP_125300786.1
Ochrobactrum sp. EEELCW01	99%	99.03%	WP_209418662.1
Ochrobactrum sp. UNC390CL2Tsu3S39	99%	98.71%	WP_029928115.1
Brucella/Ochrobactrum group	99%	99.03%	WP_094515040.1
Brucella anthropi	99%	99.03%	WP_011982652.1
Brucella anthropi	99%	98.71%	WP_151607006.1
Brucella anthropi	99%	98.39%	WP_061346706.1

eryD protein BLAST alignments

Strain	Query coverage	Percent identity	Accession
Brucella/Ochrobactrum group	94%	100.00%	WP_011982651.1
Brucella/Ochrobactrum group	94%	99.67%	WP_010660868.1
Brucella/Ochrobactrum group	94%	99.67%	WP_094515039.1
Brucella anthropi	94%	99.34%	WP_194531703.1
Brucella/Ochrobactrum group	94%	99.34%	WP_151607008.1
Brucella anthropi	94%	99.67%	WP_125300787.1
Brucella tritici	94%	99.34%	WP_151557007.1
Brucella tritici	94%	99.34%	WP_151676965.1
Brucella anthropi	94%	99.34%	WP_061346707.1
Brucella/Ochrobactrum group	94%	99.00%	WP_109986727.1

eryE protein BLAST alignments

Strain	Query coverage	Percent identity	Accession
Brucella/Ochrobactrum group	99%	100.00%	WP_011982655.1
Brucella/Ochrobactrum group	99%	99.54%	WP_010660872.1
Brucella/Ochrobactrum group	99%	97.24%	WP_121986228.1
Brucella anthropi	99%	99.54%	WP_125334792.1
Brucella/Ochrobactrum group	99%	99.08%	WP_105529314.1
Brucella anthropi	99%	99.08%	WP_194531702.1
unclassified Ochrobactrum	99%	96.77%	WP_114215989.1
Brucella cytisi	99%	98.62%	WP_071630412.1
Brucella oryzae	99%	97.24%	WP_211693538.1
Brucella oryzae	99%	96.31%	WP_104755620.1

eryF protein BLAST alignments

Strain	Query coverage	Percent identity	Accession
Brucella/Ochrobactrum group	99%	100.00%	WP_036587143.1
Brucella anthropi	99%	99.81%	WP_011982656.1
Brucella/Ochrobactrum group	99%	99.61%	WP_010660873.1
Brucella/Ochrobactrum group	99%	99.61%	WP_151607001.1
Brucella/Ochrobactrum group	99%	99.42%	WP_094515043.1
Ochrobactrum sp. MYb49	99%	99.42%	WP_105529313.1
Brucella anthropi	99%	99.42%	WP_061346701.1
Ochrobactrum sp. UNC390CL2Tsu3S39	99%	99.42%	WP_029928107.1
Brucella cytisi	99%	99.03%	WP_071630413.1
Brucella cytisi	99%	98.83%	NKC51647.1

eryG protein BLAST alignments

Strain	Query coverage	Percent identity	Accession
Brucella/Ochrobactrum group	97%	100.00%	WP_010660874.1
Brucella/Ochrobactrum group	97%	99.71%	WP_151663530.1
Brucella pecoris	97%	99.71%	WP_140020911.1
Brucella/Ochrobactrum group	97%	99.42%	WP_114215987.1
Brucella/Ochrobactrum group	97%	99.42%	WP_061346700.1
Brucella oryzae	97%	99.42%	WP_104755618.1
Brucella daejeonensis	97%	98.55%	WP_183651231.1
Brucella vulpis	97%	98.27%	CUW45266.1
Ochrobactrum sp. CGA5	97%	98.27%	WP_139975251.1
Brucella	97%	98.55%	WP_008511001.1

eryH protein BLAST alignments

Strain	Query coverage	Percent identity	Accession
Brucella/Ochrobactrum group	99%	100.00%	WP_036587137.1
Brucella/Ochrobactrum group	99%	99.22%	WP_029928118.1
Brucella/Ochrobactrum group	99%	99.22%	WP_151607009.1
Brucella anthropi	99%	98.83%	WP_151663532.1
Brucella lupini	99%	98.83%	WP_094515038.1
Brucella/Ochrobactrum group	99%	98.44%	WP_036582067.1
Brucella anthropi	99%	98.05%	WP_125300788.1
Brucella anthropi	99%	98.05%	WP_061346709.1
Brucella anthropi	99%	98.05%	WP_010660866.1
Brucella anthropi	99%	97.66%	WP_011982649.1

eryI protein BLAST alignments

Strain	Query coverage	Percent identity	Accession
Brucella anthropi ATCC 49188	99%	100.00%	ABS15613.1
Brucella/Ochrobactrum group	98%	100.00%	WP_029928121.1
Brucella cytisi	98%	99.34%	WP_071630405.1
Brucella tritici	98%	99.34%	WP_151557013.1
Brucella	98%	99.34%	WP_010660865.1
Brucella anthropi	98%	99.34%	WP_125300863.1
Brucella/Ochrobactrum group	98%	98.68%	WP_151607012.1
unclassified Ochrobactrum	98%	98.67%	WP_114215994.1
Brucella melitensis bv. 1 str. 16M	98%	97.35%	5IFZ_A
Brucella abortus str. 2308	98%	97.35%	EEP61887.1

eryR protein BLAST alignments

Strain	Query coverage	Percent identity	Accession
Brucella anthropi	99%	100.00%	WP_210342020.1
Brucella anthropi	99%	100.00%	WP_210271813.1
Brucella anthropi	99%	100.00%	HBQ33941.1
Brucella/Ochrobactrum group	99%	100.00%	WP_010660867.1
Ochrobactrum sp. P20RRXII	99%	100.00%	WP_011982650.1
Brucella/Ochrobactrum group	99%	100.00%	WP_029928117.1
Brucella	99%	99.55%	WP_061346708.1
Brucella anthropi	99%	99.55%	WP_194531704.1
Brucella tritici	99%	98.65%	WP_151557009.1
Brucella/Ochrobactrum group	99%	97.76%	WP_109986726.1

Supplementary Figure 5. Protein BLAST alignments of erythritol catabolism-associated genes.

Top ten protein alignments of every erythritol catabolism-associated genes (eryA/B/C/D/E/F/G/H/I/R) in *Ochrobactrum* spp..

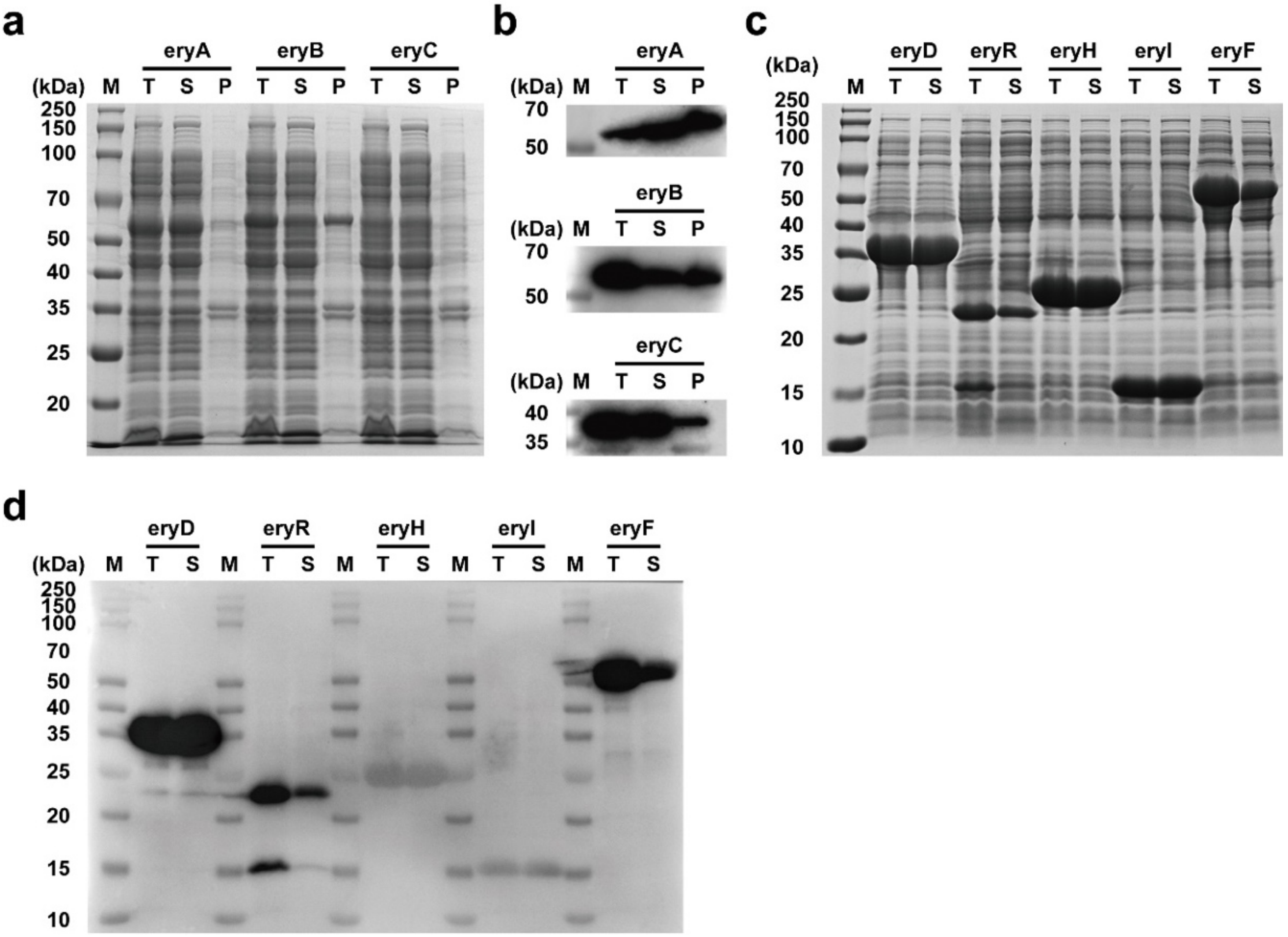

Supplementary Figure 6. *In vivo* expression of erythritol catabolism-associated genes.

(**a**) SDS-PAGE analysis of gene expression of eryA (with N-terminal 6xHis tag, 56.2 kDa), eryB (with N-terminal 6xHis tag, 57.2 kDa), and eryC (with N-terminal 6xHis tag, 35.8 kDa). All these three genes were expressed by plasmids pFB158, pFB159, and pFB160, respectively. *E. coli* Mach1-T1 harboring each above plasmid was initially inoculated with overnight culture (5 mL LB at 37°C and 250 rpm for 16 h) as a volume ratio of 1:500, and then incubated in 5 mL new LB medium at 37°C and 250 rpm. When OD_{600} reached 0.6, 1% arabinose (w/v) was added and incubated for another 16 h at 20°C and 250 rpm. Then, cell pellets were resuspended (equal volume) with 1x phosphate-buffered saline (pH 7.4) and lysed by sonication. The total cell lysate "T", supernatant "S", and pellets "P" were analyzed by SDS-PAGE. (**b**) Western-Blot analysis of the same samples in (**a**). (**c**) SDS-PAGE analysis of gene expression of eryD (with N-terminal 6xHis tag, 34.5 kDa), eryR (with N-terminal 6xHis tag, 24.8 kDa), eryH (with N-terminal 6xHis tag, 28.8 kDa), eryI (with N-terminal 6xHis tag, 16.6 kDa), and eryF (with N-terminal 6xHis tag, 56.2 kDa). All these five genes were expressed by plasmids pFB161, pFB167, pFB165, pFB166, and pFB163, respectively. *E. coli* BL21(DE3) harboring each above plasmid was initially inoculated with overnight culture (5 mL LB at 37°C and 250 rpm for 16 h) as a volume ratio of 1:500, and then incubated in 5 mL new LB medium at 37°C and 250 rpm. When OD_{600} reached 0.6, 0.5 mM IPTG was added and incubated for another 16 h at 20°C and 250 rpm. Then, cell pellets were resuspended (equal volume) with 1x phosphate-buffered saline (pH 7.4) and lysed by sonication. The total cell lysate "T", supernatant "S", and pellets "P" were analyzed by SDS-PAGE. (**d**) Western-Blot analysis of the same samples in (**c**).

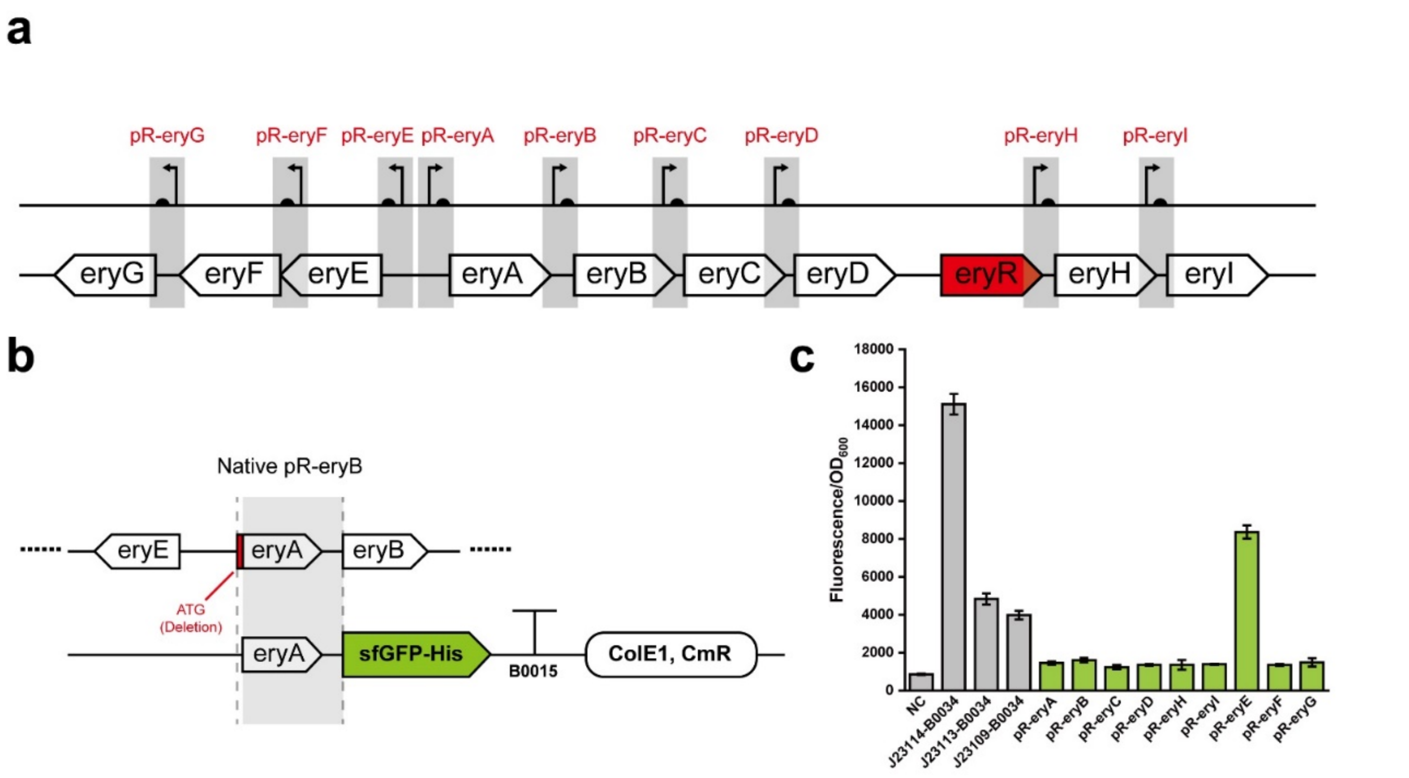

Supplementary Figure 7. Native promoter-RBS (pR-) composite part characterization in the erythritol gene cluster. (**a**) Schematic of promoter-RBS (pR-) composite part localization. Note that the function of eryR was not successfully characterized, thus here we did not test pR-eryR. (**b**) Schematic procedure of "pR-ery" reporter plasmid construction (pFB177 to pFB185). Here we illustrate the construction of pR-eryB-sfGFP reporter plasmid as an example. The DNA sequence between "eryA CDS except the initiation codon ATG" and "the last nucleotide before eryB CDS" was considered as pR-eryB. This part was then assembled with the following sfGFP (6xHis) and a terminator B0015 in the vector pSB1C3 (pFB178). (**c**) Normalized fluorescence of all nine "pR-ery" parts. All the measurements were performed in *E. coli* Mach1-T1 with three biological replicates. "NC" represents *E. coli* Mach1-T1 without plasmids. The other three gray columns correspond to pFB291, pFB290, and pFB289, respectively, to act as positive controls (iGEM standard biological parts). The results indicate that all nine native composite parts are not strong regulatory elements compared to the reference "J23114-B0034".

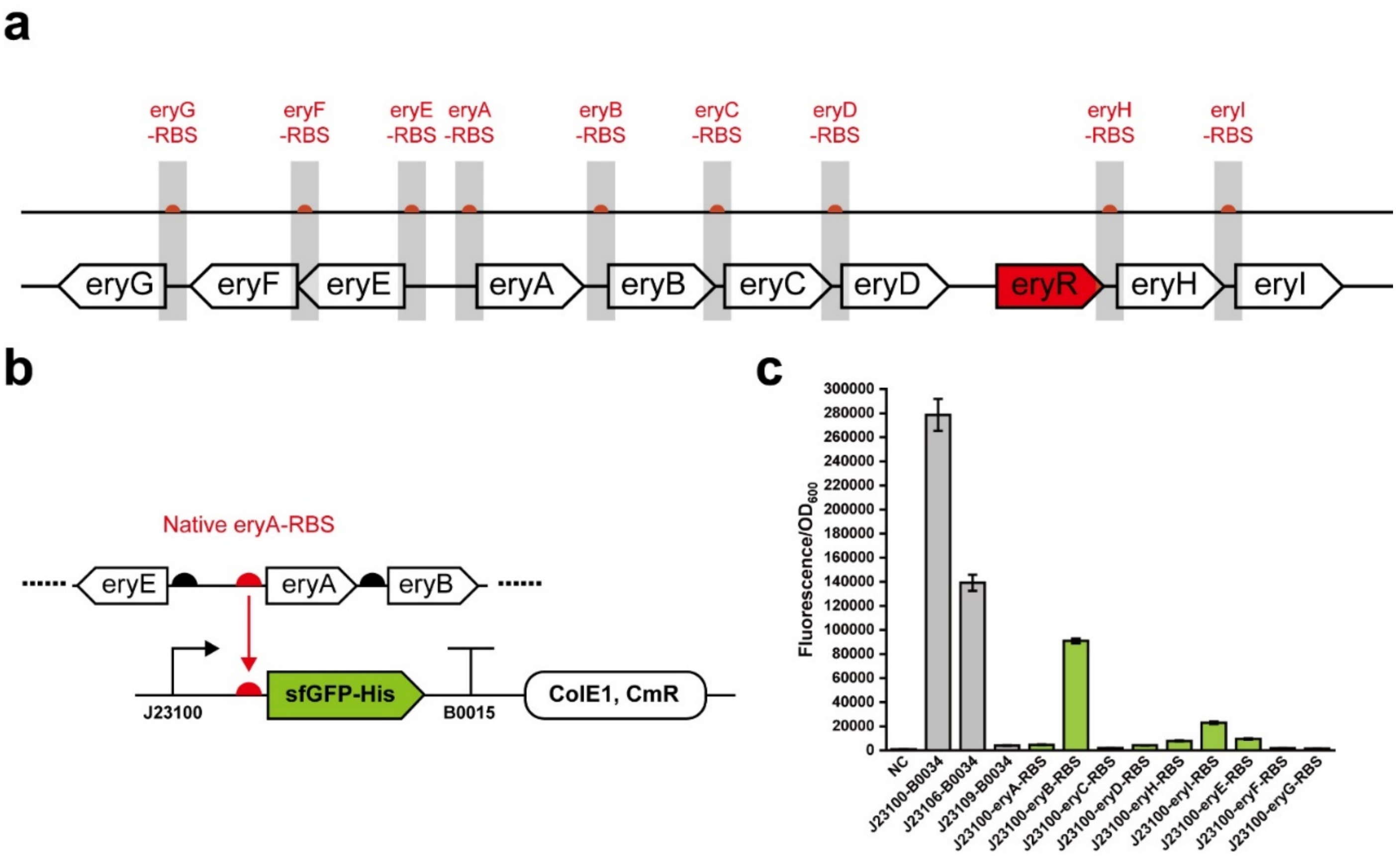

Supplementary Figure 8. Native RBS characterization in the erythritol gene cluster.
(**a**) Schematic of RBS localization. Note that the function of eryR was not successfully characterized, thus here we did not test eryR-RBS. (**b**) Schematic procedure of "ery-RBS" reporter plasmid construction (pFB168 to pFB176). Here we illustrate the construction of J23100-eryA-RBS-sfGFP reporter plasmid as an example. The putative "eryA-RBS" sequence (approximately 40 bp) was assembled with the promoter J23100, sfGFP (6xHis), and a terminator B0015 in the vector pSB1C3 (pFB168). (**c**) Normalized fluorescence of all nine RBS parts. All the measurements were performed in *E. coli* Mach1-T1 with three biological replicates. "NC" represents *E. coli* Mach1-T1 without plasmids. The other three gray columns correspond to pFB286, pFB288, and pFB289, respectively, to act as positive controls (iGEM standard biological parts). The results indicate that "eryB-RBS" is a relatively strong RBS, but the other eight RBSs are not strong.

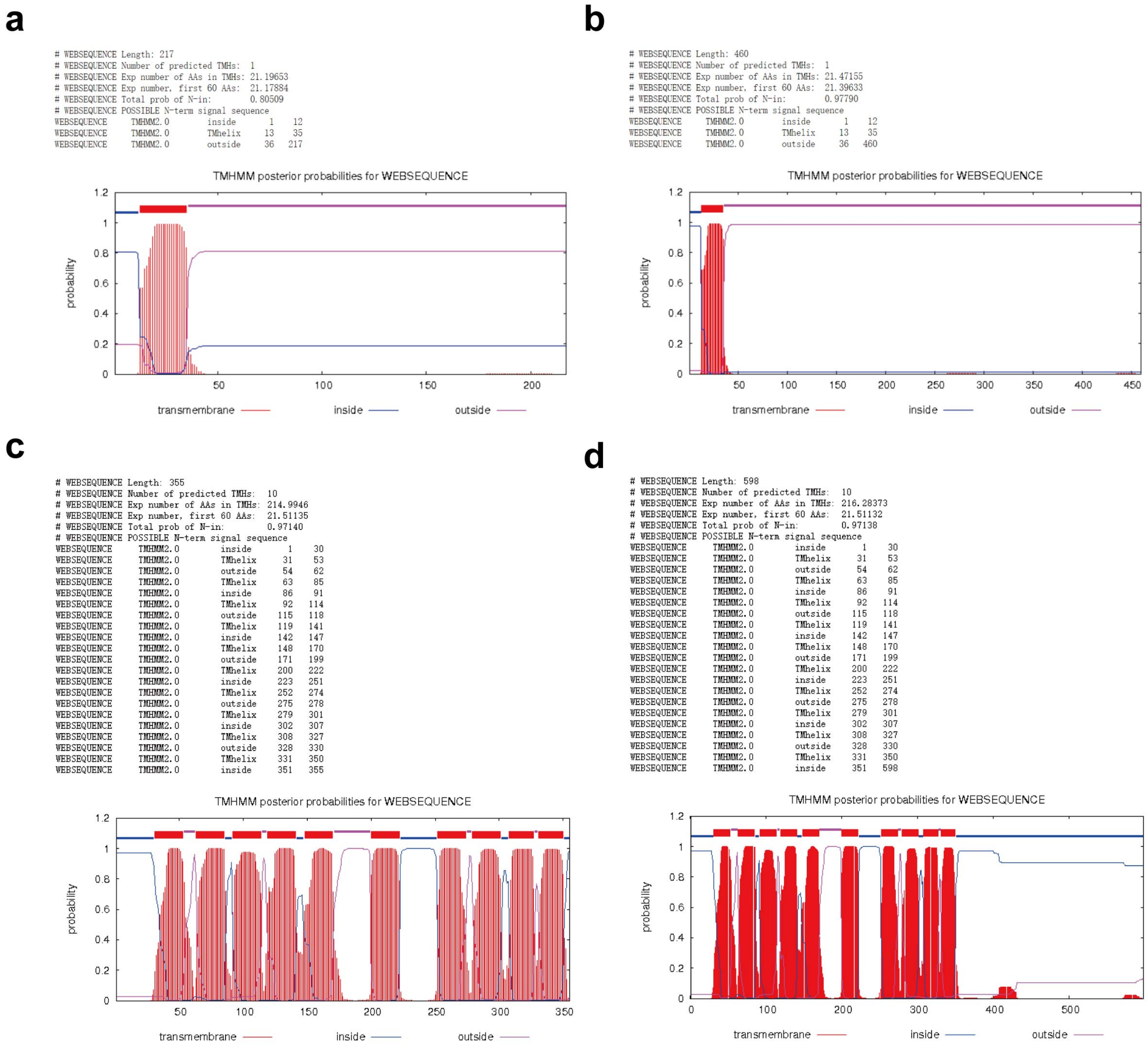

Supplementary Figure 9. Prediction of eryE and eryG transmembrane topology by TMHMM - 2.0[6].

(**a**) eryE transmembrane topology prediction indicates that it is a single-pass membrane protein.

(**b**) eryE-sfGFP fusion protein prediction indicates the same topology as eryE.

(**c**) eryG transmembrane topology prediction indicates that it is a ten times transmembrane protein.

(**d**) eryG-sfGFP fusion protein prediction indicates the same topology as eryG.

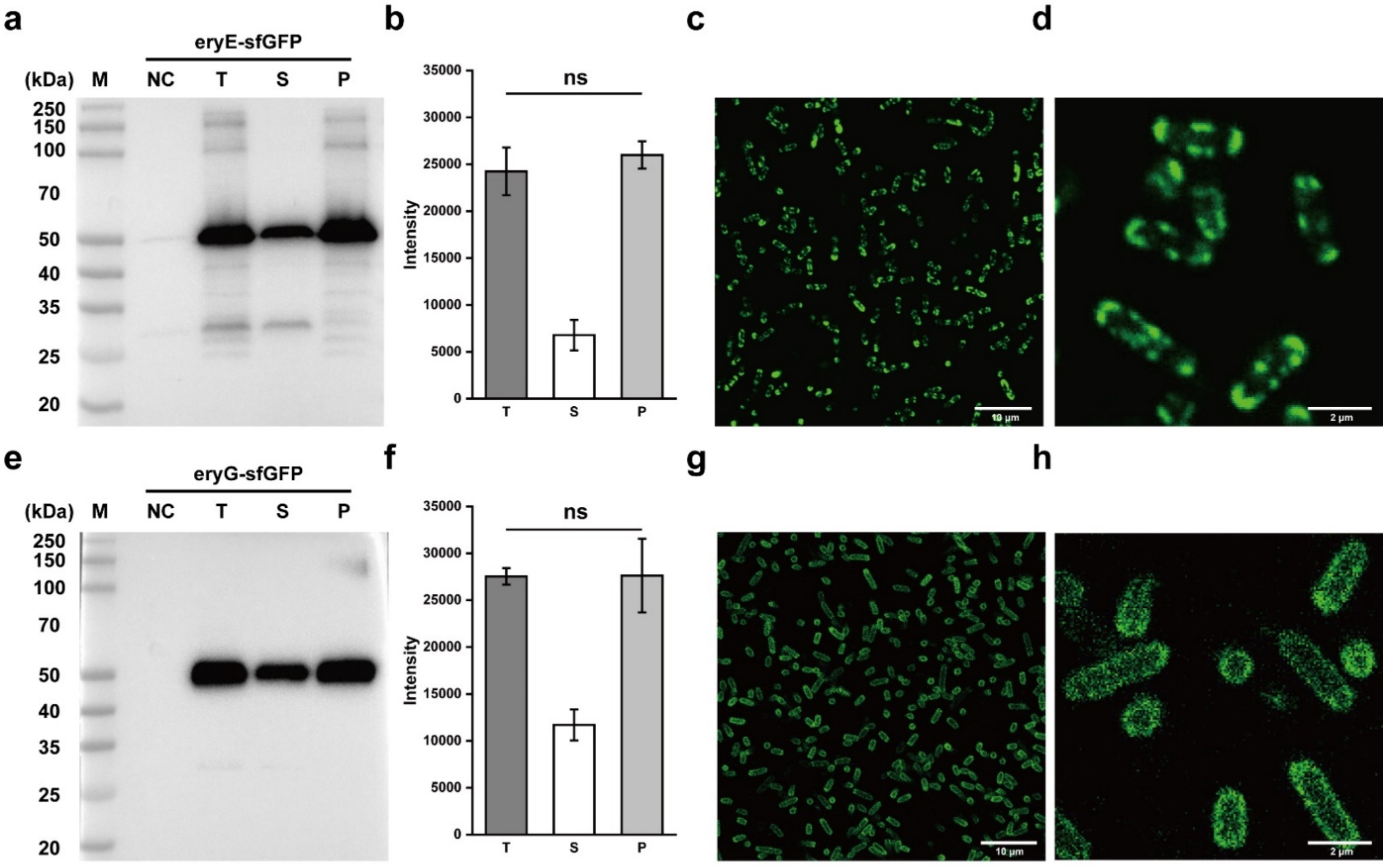

Supplementary Figure 10. eryE-sfGFP and eryG-sfGFP characterization.

(**a**) Western-Blot analysis of eryE-sfGFP(6xHis) (50.4 kDa). *E. coli* Mach1-T1 harboring the plasmid pFB162 was initially inoculated with overnight culture (5 mL LB medium at 37°C and 250 rpm for 16 h) as a volume ratio of 1:500, and then incubated in 5 mL new LB medium at 37°C and 250 rpm. When OD_{600} reached 0.6, 1% arabinose (w/v) was added and incubated for another 6 h at 30°C and 250 rpm. Then, cell pellets were resuspended (equal volume) with 1x phosphate-buffered saline (pH 7.4) and lysed by sonication. The total cell lysate “T”, supernatant “S”, and pellets “P” were analyzed by Western-Blot. “NC” represents the total cell lysate of *E. coli* Mach1-T1 harboring pFB162 without arabinose induction. (**b**) Quantitative analysis and comparison of protein (eryE-sfGFP) band densities determined by ImageJ. The data showed that protein band densities from the total and pellet fractions were similar without significant difference (ns indicates p-value > 0.05). Each value (mean ± s.d.) is calculated with three biological replicates and the error bar represents the standard deviation (s.d.). (**c**) Confocal laser scanning microscopy image of *E. coli* Mach1-T1 expressing eryE-sfGFP. Green border indicates the transmembrane localization of eryE-sfGFP. (**d**) Partially enlarged view of the image in (**c**). (**e**) Western-Blot analysis of eryG-sfGFP(6xHis) (64.6 kDa). *E. coli* Mach1-T1 harboring the plasmid pFB164 was initially incubated in 5 mL LB medium at 37°C and 250 rpm. When OD_{600} reached 0.6, 1% arabinose (w/v) was added and incubated for another 6 h at 30°C and 250 rpm. Then, cell pellets were resuspended (equal volume) with 1x phosphate-buffered saline (pH 7.4) and lysed by sonication. The total cell lysate “T”, supernatant “S”, and pellets “P” were analyzed by Western-Blot. “NC” represents the total cell lysate of *E. coli* Mach1-T1 harboring plasmid pFB164 without arabinose induction. (**f**) Quantitative analysis and comparison of protein (eryG-sfGFP) band densities determined by ImageJ. The data showed that protein band densities from the total and pellet fractions were similar without significant difference (ns indicates p-value > 0.05). Each value (mean ± s.d.) is calculated with three biological replicates and the error bar represents the standard deviation (s.d.). (**g**) Confocal laser scanning microscopy image of *E. coli* Mach1-T1 expressing eryG-sfGFP. Green border indicates the transmembrane localization of eryG-sfGFP. (**h**) Partially enlarged view of the image in (**g**).

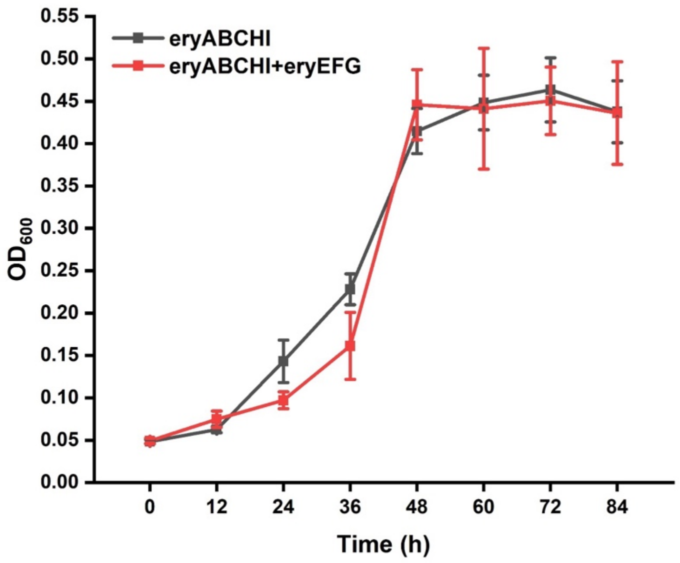

Supplementary Figure 11. Comparison of cell growth between two strains with and without the erythritol ABC-transporter (i.e., eryE/F/G).

Two strains of *E. coli* MG1655 were used, each harboring the erythritol catabolic cluster (pFB147, black line) or harboring both the erythritol catabolic cluster and the erythritol ABC-transporter (pFB157, red line). Both strains were cultivated in M9-erythritol medium for 84 h and OD_{600} values were measured after each 12 h. The growth curves were calculated with three biological replicates and the error bars represent the standard deviation (s.d.).

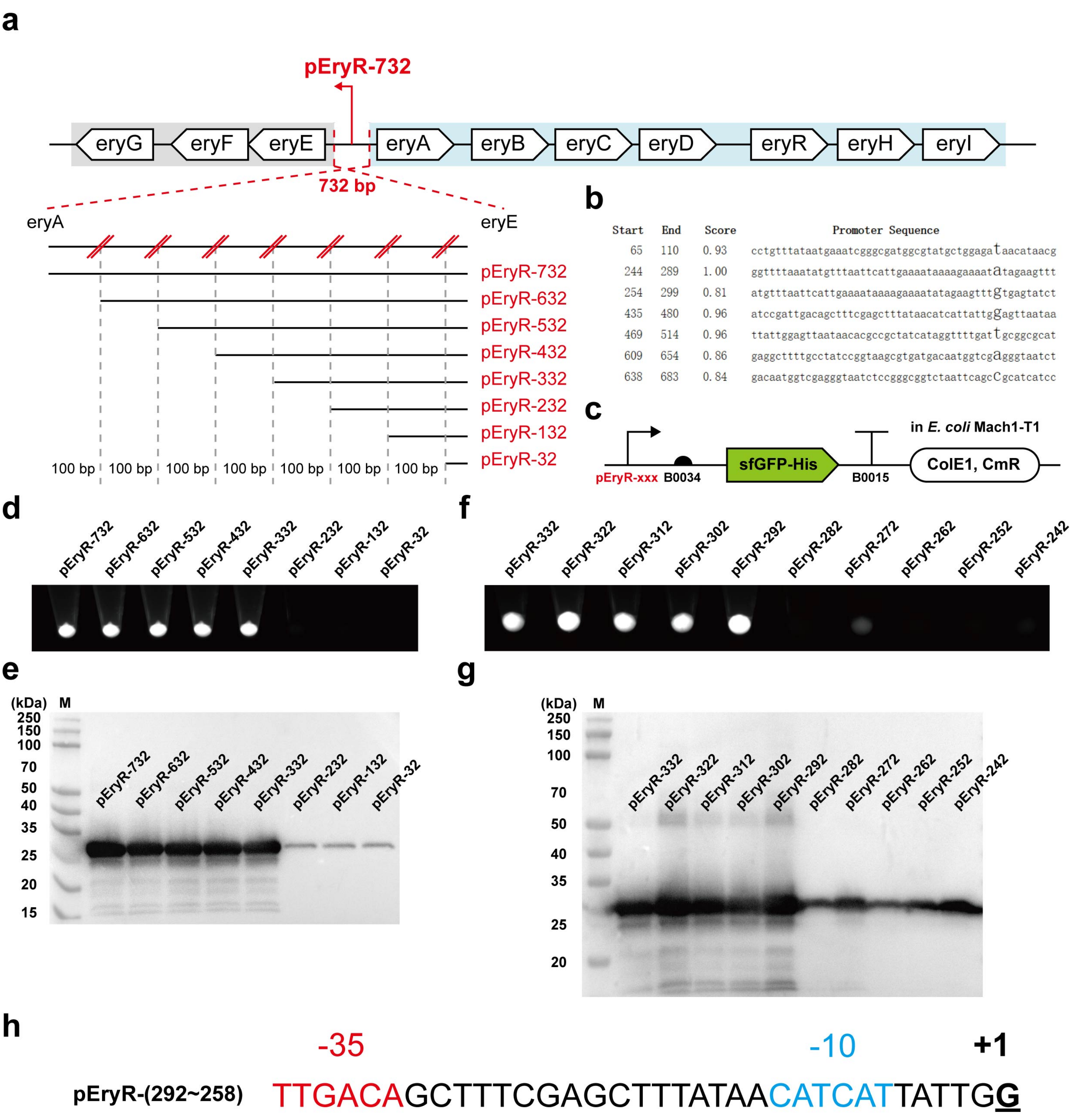

Supplementary Figure 12. pEryR characterization.

(**a**) Schematic characterization process of pEryR. The 732 bp DNA gap between eryA and eryE was initially acquired as pEryR-732, and then 100 bp-gradient truncated pEryR variants were constructed into B0034-sfGFP (6xHis)-based reporter plasmids (pFB239 to pFB243, pFB253 to pFB255).

(**b**) Promoter prediction result of pEryR-732 by "BDGP: Neural Network Promoter Prediction"[7].

(**c**) Schematic B0034-sfGFP (6xHis)-based reporter plasmid design. All the following characterization is in *E. coli* strain Mach1-T1.

(**d**) *E. coli* Mach1-T1 pellets were collected from 1.5 mL overnight LB culture (37°C, 250 rpm for 16 h). Brighter pellets indicated more sfGFP expression. The result showed that the core promoter region might be located between pEryR-332 and pEryR-232. Fluorescence images were generated by UVP ChemStudio (analytikjena).

(**e**) Western-Blot analysis of sfGFP (6xHis) relative expression level, the samples were the same as (**d**), the cell pellets were resuspended (equal volume) with 1x phosphate-buffered saline (pH 7.4) and lysed by sonication, the total cell lysate was analyzed.
(**f**) 10 bp-gradient truncated pEryR variants characterization. The process was the same as (**d**), and the result showed that the core promoter region might be located between pEryR-292 and pEryR-282.
(**g**) Western-Blot analysis of sfGFP (6xHis) relative expression level, the samples were the same as (**f**).
(**h**) Schematic of pEryR core region sequence after the truncated characterization. -35, -10, and +1 regions were shown as red, blue, and underlined bold black, respectively.

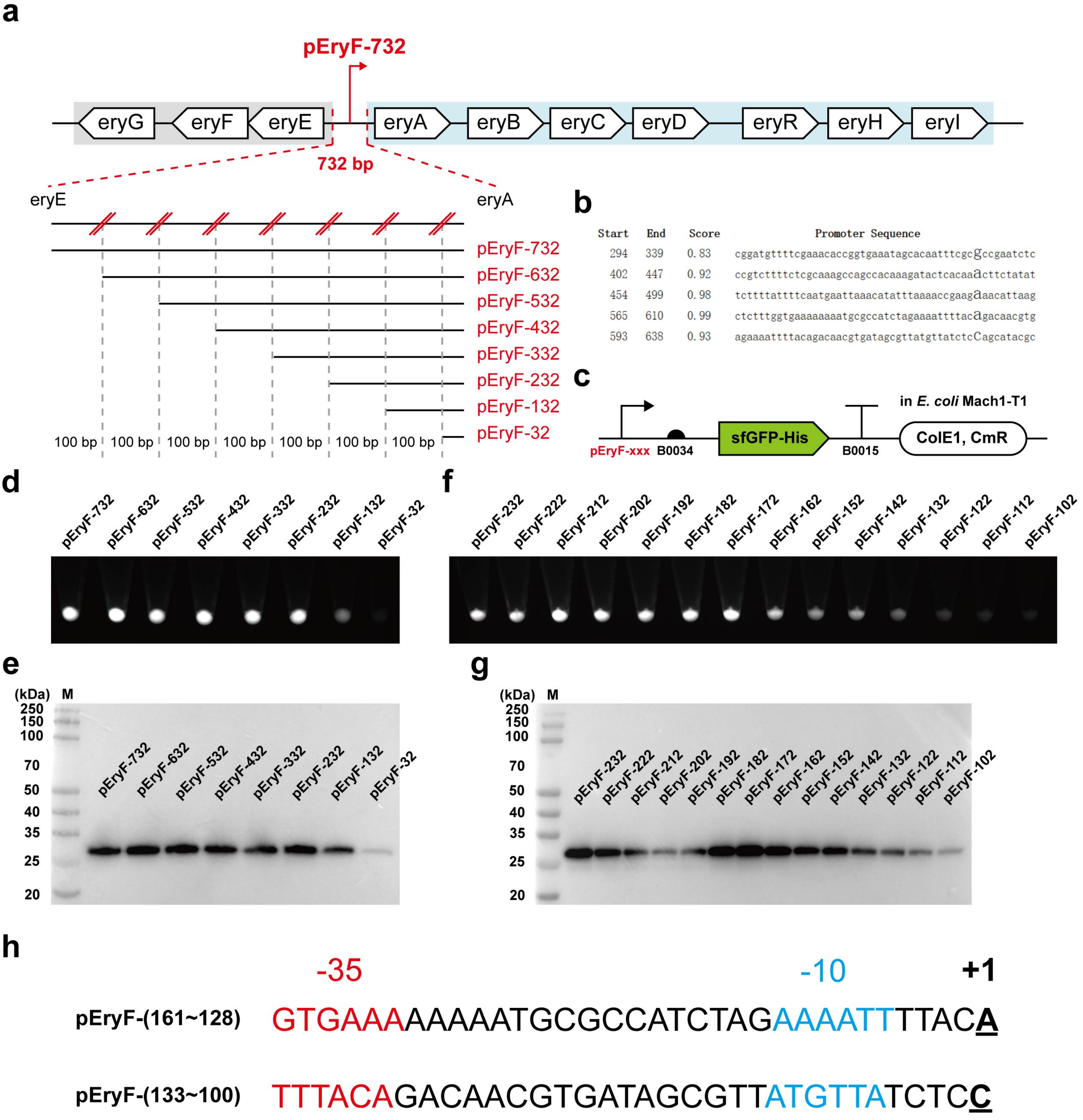

Supplementary Figure 13. pEryF characterization.

(**a**) Schematic characterization process of pEryF. The 732 bp DNA gap between eryE and eryA was initially acquired as pEryF-732, and then 100 bp-gradient truncated pEryF variants were constructed into B0034-sfGFP (6xHis)-based reporter plasmids (pFB186 to pFB191, pFB210, and pFB292).

(**b**) Promoter prediction result of pEryF-732 by "BDGP: Neural Network Promoter Prediction"[7].

(**c**) Schematic B0034-sfGFP (6xHis)-based reporter plasmid design. All the following characterization is in *E. coli* strain Mach1-T1.

(**d**) *E. coli* Mach1-T1 pellets were collected from 1.5 mL overnight LB culture (37°C, 250 rpm for 16 h). Brighter pellets indicated more sfGFP expression. The result showed that the core promoter region might be located between pEryF-232 and pEryF-32. Fluorescence images were generated by UVP ChemStudio (analytikjena).
(**e**) Western-Blot analysis of sfGFP (6xHis) relative expression level, the samples were the same as (**d**), the cell pellets were resuspended (equal volume) with 1x phosphate-buffered saline (pH 7.4) and lysed by sonication, the total cell lysate was analyzed.
(**f**) 10 bp-gradient truncated pEryF variants characterization. The process was the same as (**d**), the result showed that the core promoter region might be located between pEryF-172 and pEryF-102.
(**g**) Western-Blot analysis of sfGFP (6xHis) relative expression level, the samples were the same as (**f**).
(**h**) Schematic of two pEryF core region sequences after the truncated characterization. -35, -10, and +1 regions were shown as red, blue, and underlined bold black, respectively.

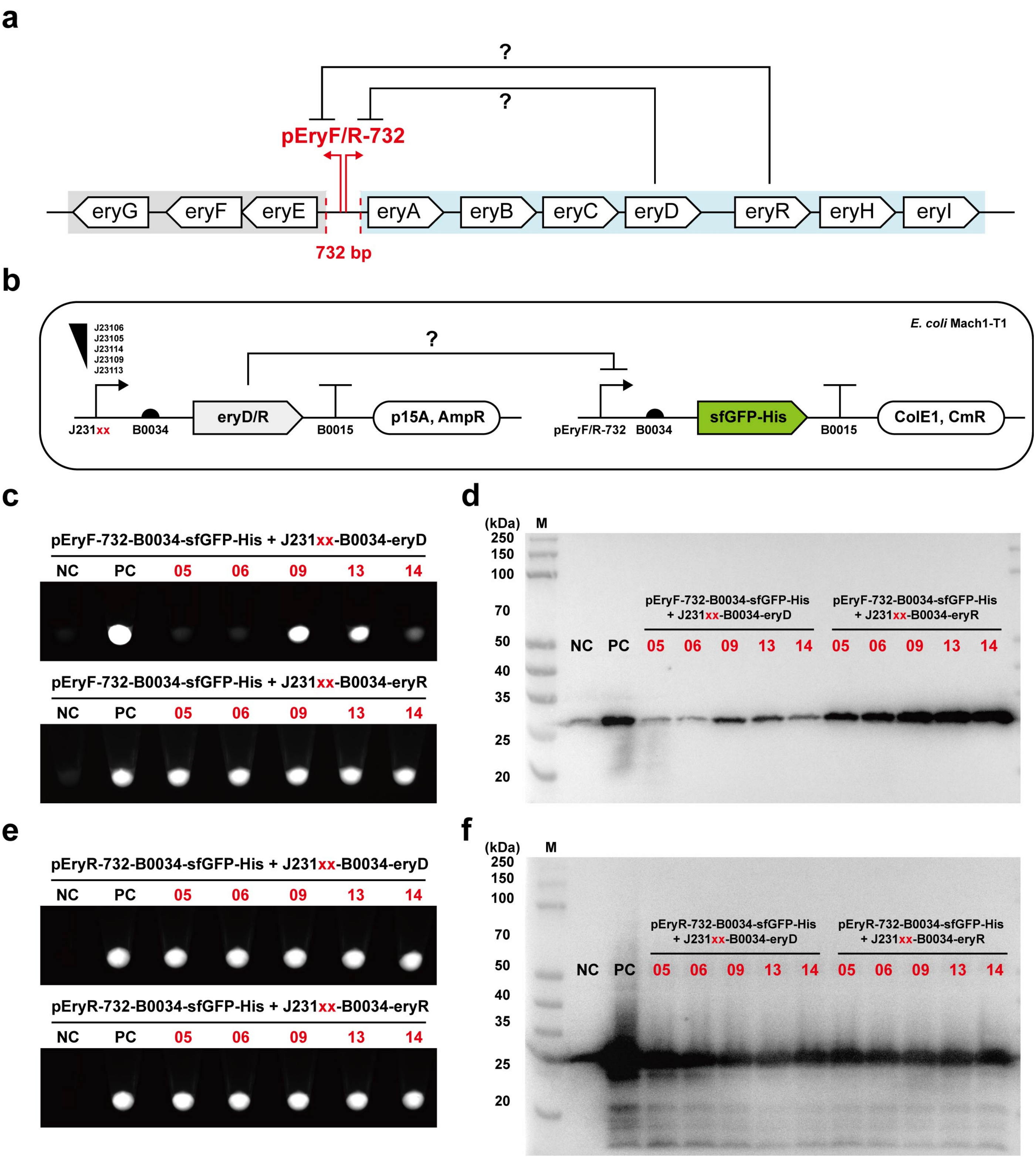

Supplementary Figure 14. eryD and eryR characterization.

(**a**) Schematic characterization process of eryD and eryR. We assumed that eryD/eryR might regulate erythritol cluster, and the long DNA gap between eryE and eryA might locate eryD/eryR binding sites in both forward and reverse directions.

(**b**) Schematic compatible plasmids design in *E. coli* Mach1-T1. Strength-gradient promoters were organized with RBS B0034, eryD/eryR coding sequences, and terminator B0015 (pFB260 to pFB269). The other two compatible plasmids (pFB186 and pFB239) were used as reporters.

(**c**) *E. coli* Mach1-T1 pellets were collected from 1.5 mL overnight LB culture (37°C, 250 rpm for 16 h). Brighter pellets indicated more sfGFP expression. The result showed that there was a putative eryD DNA-binding site on pEryF-732, but eryR not. Furthermore, a higher expression level of eryD caused lower sfGFP expression. "NC" represents *E. coli* Mach1-T1 without plasmids, and "PC" represents *E. coli* Mach1-T1 with plasmid pFB186. Fluorescence images were generated by UVP ChemStudio (analytikjena).
(**d**) Western-Blot analysis of sfGFP (6xHis) relative expression level, the samples were the same as (**c**), the cell pellets were resuspended (equal volume) with 1x phosphate-buffered saline (pH 7.4) and lysed by sonication, the total cell lysate was analyzed.
(**e**) *E. coli* Mach1-T1 pellets were collected from 1.5 mL overnight LB culture (37°C, 250 rpm for 16 h). Brighter pellets indicated more sfGFP expression. The result showed that there were not any eryD or eryR DNA-binding sites on pEryR-732. "NC" represents *E. coli* Mach1-T1 without plasmids, and "PC" represents *E. coli* Mach1-T1 with plasmid pFB239. Fluorescence images were generated by UVP ChemStudio (analytikjena).
(**f**) Western-Blot analysis of sfGFP (6xHis) relative expression level, the samples were the same as (**e**), the cell pellets were resuspended (equal volume) with 1x phosphate-buffered saline (pH 7.4) and lysed by sonication, the total cell lysate was analyzed.

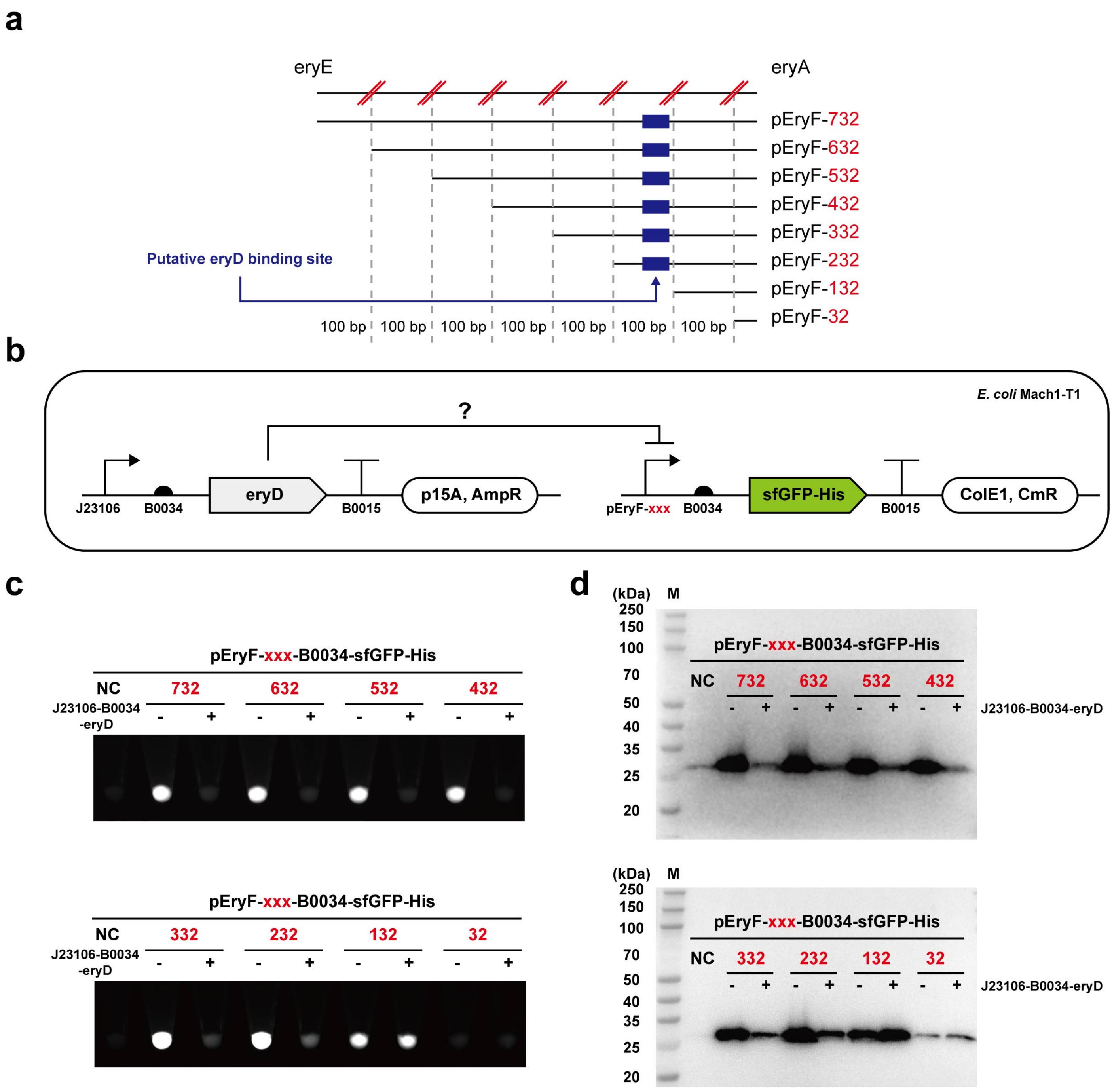

Supplementary Figure 15. The first step for characterization of eryD binding site prefix.

(**a**, **b**) Schematic characterization process of putative eryD binding site (eryO).

(**c**) *E. coli* Mach1-T1 pellets were collected from 1.5 mL overnight LB culture (37°C, 250 rpm for 16 h). Brighter pellets indicated more sfGFP expression. "NC" represents *E. coli* Mach1-T1 without plasmids, and the "-/+" means the *E. coli* strains without or with plasmid pFB261. The result showed that the eryO region might be located between pEryF-232 and pEryF-132. Fluorescence images were generated by UVP ChemStudio (analytikjena).

(**d**) Western-Blot analysis of sfGFP (6xHis) relative expression level, the samples were the same as (**c**), the cell pellets were resuspended (equal volume) with 1x phosphate-buffered saline (pH 7.4) and lysed by sonication, the total cell lysate was analyzed.

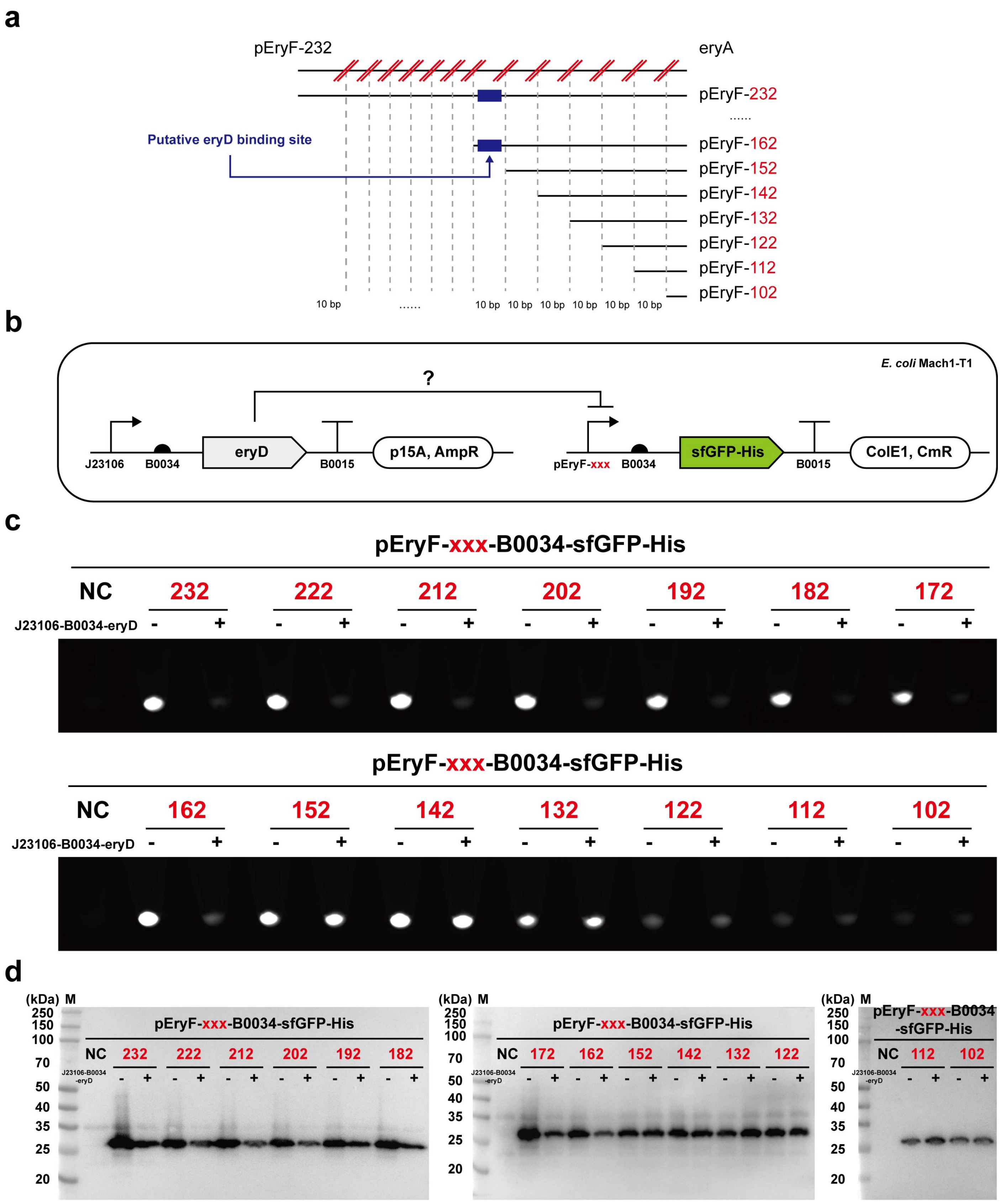

Supplementary Figure 16. The second step for characterization of eryD binding site prefix.

(**a**, **b**) Schematic characterization process of putative eryD binding site (eryO).

(**c**) *E. coli* Mach1-T1 pellets were collected from 1.5 mL overnight LB culture (37°C, 250 rpm for 16 h). Brighter pellets indicated more sfGFP expression. "NC" represents *E. coli* Mach1-T1 without plasmids, and the "-/+" means the *E. coli*

strains without or with plasmid pFB261. The result showed that the prefix of eryO region might be located between pEryF-162 and pEryF-152. Fluorescence images were generated by UVP ChemStudio (analytikjena).
(**d**) Western-Blot analysis of sfGFP (6xHis) relative expression level, the samples were the same as (**c**), the cell pellets were resuspended (equal volume) with 1x phosphate-buffered saline (pH 7.4) and lysed by sonication, the total cell lysate was analyzed.

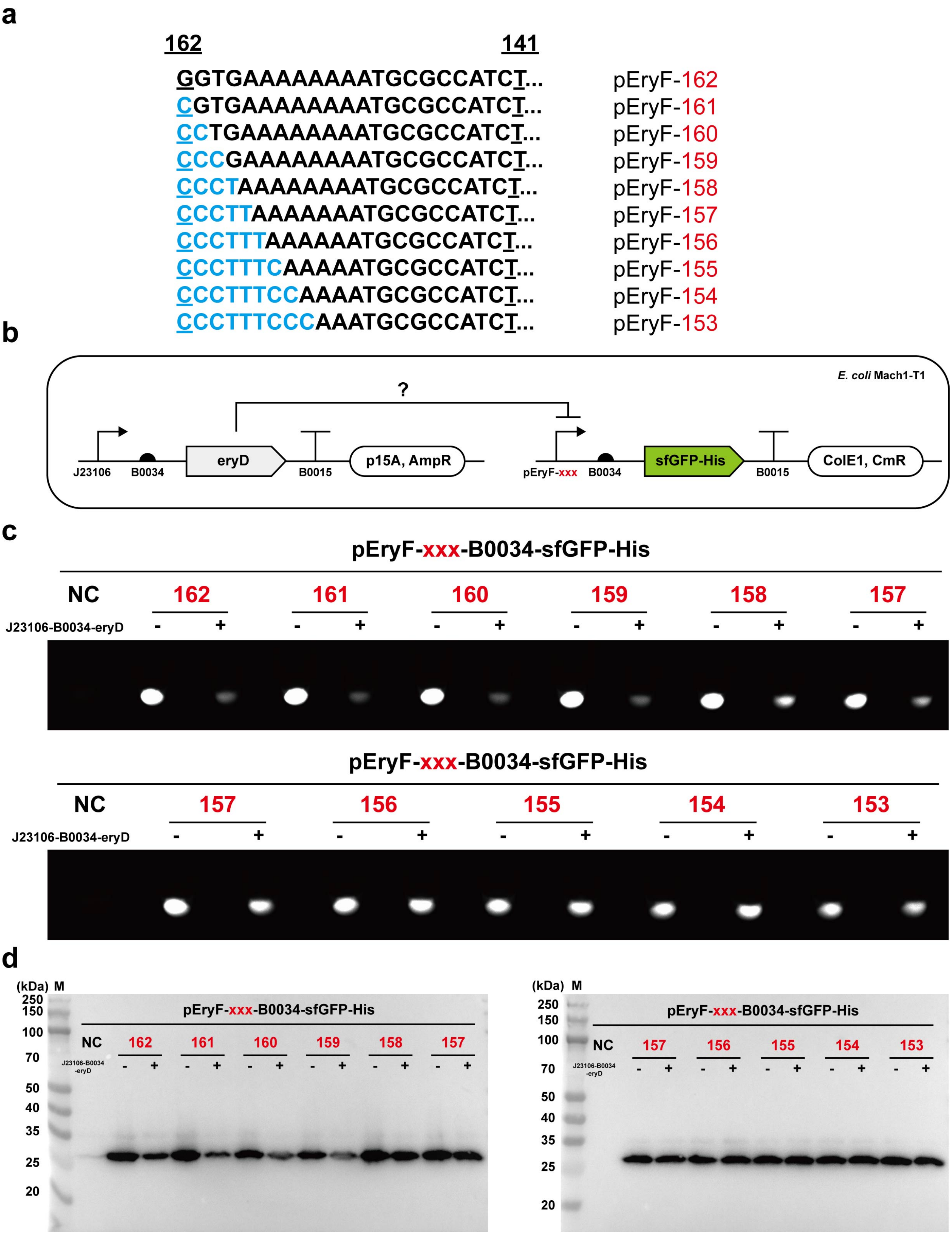
a
162
141
GGTGAAAAAAAATGCGCCATCT... pEryF-162
CGTGAAAAAAAATGCGCCATCT... pEryF-161
CCTGAAAAAAAATGCGCCATCT... pEryF-160
CCCGAAAAAAAATGCGCCATCT... pEryF-159
CCCTAAAAAAAATGCGCCATCT... pEryF-158
CCCTTAAAAAAATGCGCCATCT... pEryF-157
CCCTTTAAAAAATGCGCCATCT... pEryF-156
CCCTTTCAAAAATGCGCCATCT... pEryF-155
CCCTTTCCAAAATGCGCCATCT... pEryF-154
CCCTTTCCCAAATGCGCCATCT... pEryF-153
b
E. coli Mach1-T1
?
J23106
B0034
eryD
B0015
p15A, AmpR
pEryF-xxx
B0034
sfGFP-His
B0015
ColE1, CmR
c
pEryF-xxx-B0034-sfGFP-His
NC
162
161
160
159
158
157
J23106-B0034-eryD
- + - + - + - + - + - +
pEryF-xxx-B0034-sfGFP-His
NC
157
156
155
154
153
J23106-B0034-eryD
- + - + - + - + - +
d
(kDa) M
250
150
100
70
50
40
35
25
20
pEryF-xxx-B0034-sfGFP-His
NC
162
161
160
159
158
157
J23106-B0034-eryD
- + - + - + - + - + - +
(kDa) M
250
150
100
70
50
40
35
25
20
pEryF-xxx-B0034-sfGFP-His
NC
157
156
155
154
153
J23106-B0034-eryD
- + - + - + - + - +

Supplementary Figure 17. The third step for characterization of eryD binding site prefix.
(**a**, **b**) Schematic characterization process of putative eryD binding site (eryO).
(**c**) *E. coli* Mach1-T1 pellets were collected from 1.5 mL overnight LB culture (37°C, 250 rpm for 16 h). Brighter pellets indicated more sfGFP expression. “NC” represents *E. coli* Mach1-T1 without plasmids, and the “-/+” means the *E. coli* strains without or with plasmid pFB261. The result showed that the prefix of eryO was located in pEryF-159 (5’-GAAAAAAAAT…-3’). Fluorescence images were generated by UVP ChemStudio (analytikjena).
(**d**) Western-Blot analysis of sfGFP (6xHis) relative expression level, the samples were the same as (**c**), the cell pellets were resuspended (equal volume) with 1x phosphate-buffered saline (pH 7.4) and lysed by sonication, the total cell lysate was analyzed.

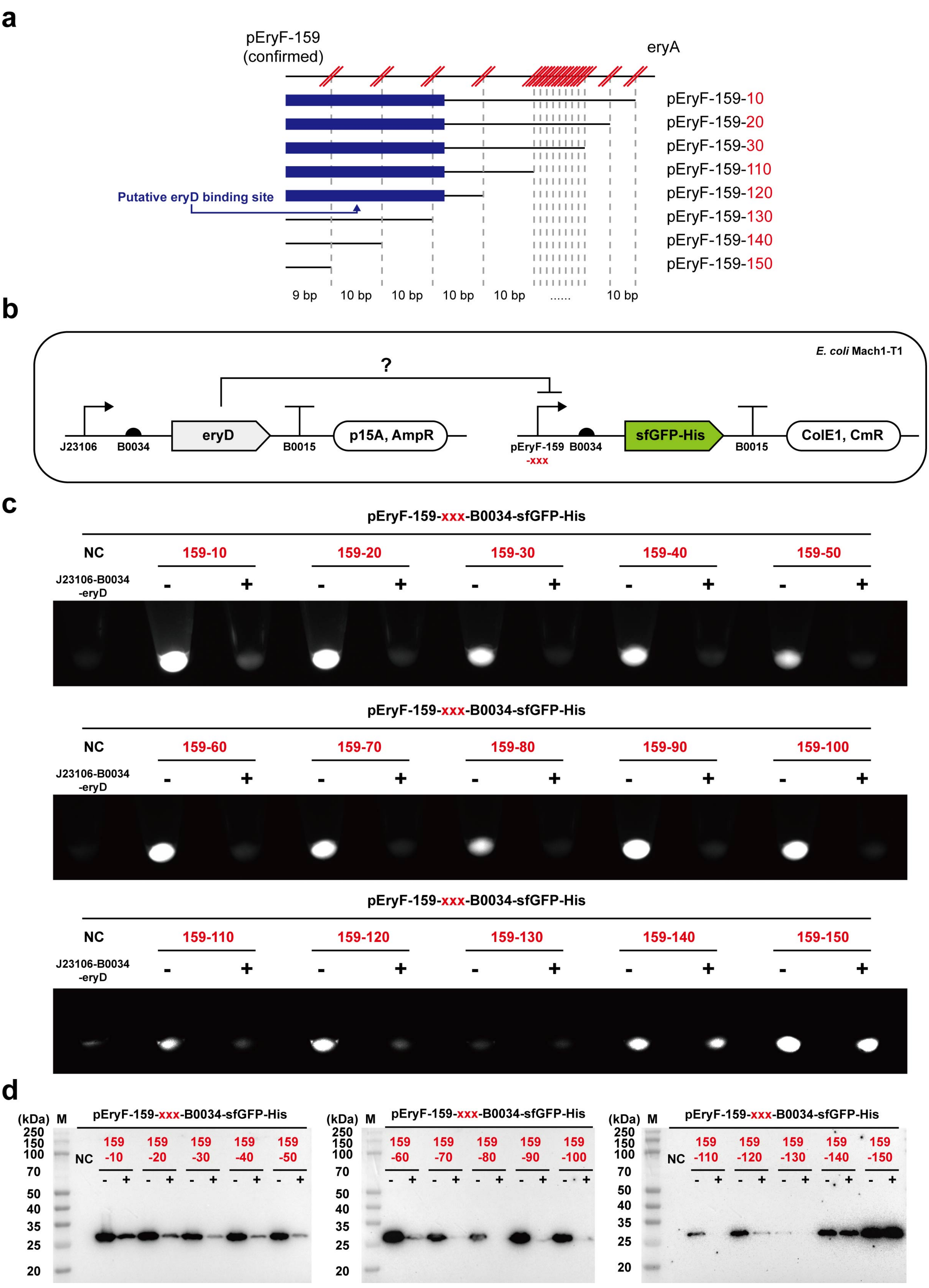

a
pEryF-159
(confirmed)
eryA
pEryF-159-10
pEryF-159-20
pEryF-159-30
pEryF-159-110
pEryF-159-120
pEryF-159-130
pEryF-159-140
pEryF-159-150
Putative eryD binding site
9 bp
10 bp
10 bp
10 bp
10 bp
......
10 bp
b
E. coli Mach1-T1
?
J23106
B0034
eryD
B0015
p15A, AmpR
pEryF-159
-xxx
B0034
sfGFP-His
B0015
ColE1, CmR
c
pEryF-159-xxx-B0034-sfGFP-His
NC
159-10
159-20
159-30
159-40
159-50
J23106-B0034
-eryD
- + - + - + - + - +
pEryF-159-xxx-B0034-sfGFP-His
NC
159-60
159-70
159-80
159-90
159-100
J23106-B0034
-eryD
- + - + - + - + - +
pEryF-159-xxx-B0034-sfGFP-His
NC
159-110
159-120
159-130
159-140
159-150
J23106-B0034
-eryD
- + - + - + - + - +
d
(kDa)
M
pEryF-159-xxx-B0034-sfGFP-His
250
150
100
70
50
40
35
25
20
NC
159 -10
159 -20
159 -30
159 -40
159 -50
- + - + - + - + - +
(kDa)
M
pEryF-159-xxx-B0034-sfGFP-His
250
150
100
70
50
40
35
25
20
159 -60
159 -70
159 -80
159 -90
159 -100
- + - + - + - + - +
(kDa)
M
pEryF-159-xxx-B0034-sfGFP-His
250
150
100
70
50
40
35
25
20
NC
159 -110
159 -120
159 -130
159 -140
159 -150
- + - + - + - + - +

Supplementary Figure 18. The first step for characterization of eryD binding site suffix.
(**a**, **b**) Schematic characterization process of putative eryD binding site (eryO).
(**c**) *E. coli* Mach1-T1 pellets were collected from 1.5 mL overnight LB culture (37°C, 250 rpm for 16 h). Brighter pellets indicated more sfGFP expression. "NC" represents *E. coli* Mach1-T1 without plasmids, and the "-/+" means the *E. coli* strain without or with plasmid pFB261. The result showed that the suffix of eryO was located between pEryF-159-120 and pEryF-159-140. Fluorescence images were generated by UVP ChemStudio (analytikjena).
(**d**) Western-Blot analysis of sfGFP (6xHis) relative expression level, the samples were the same as (**c**), the cell pellets were resuspended (equal volume) with 1x phosphate-buffered saline (pH 7.4) and lysed by sonication, the total cell lysate was analyzed.

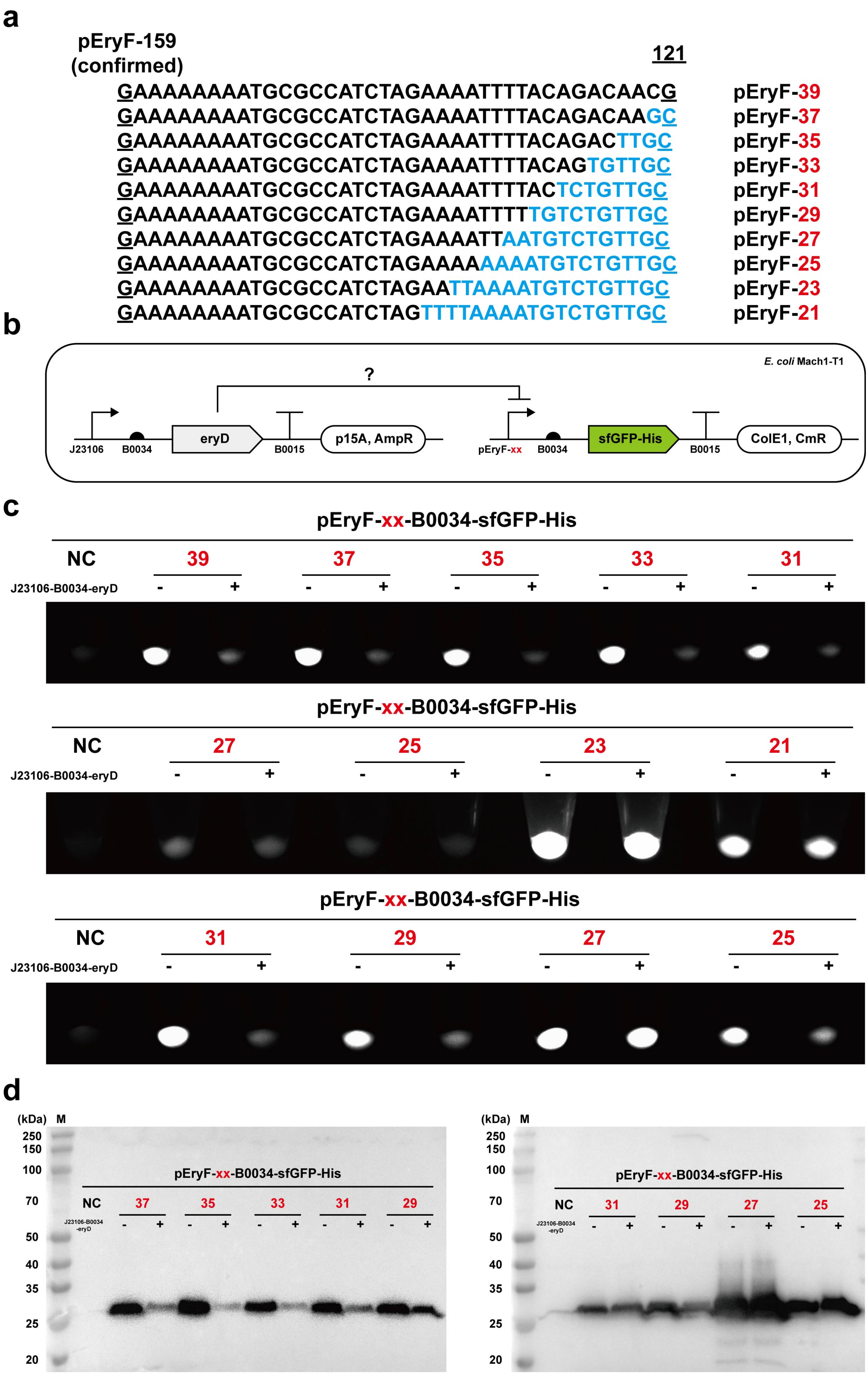
a
pEryF-159
(confirmed)
121
GAAAAAAAATGCGCCATCTAGAAAATTTTACAGACAACG pEryF-39
GAAAAAAAATGCGCCATCTAGAAAATTTTACAGACAAGC pEryF-37
GAAAAAAAATGCGCCATCTAGAAAATTTTACAGACTTGC pEryF-35
GAAAAAAAATGCGCCATCTAGAAAATTTTACAGTGTTGC pEryF-33
GAAAAAAAATGCGCCATCTAGAAAATTTTACTCTGTTGC pEryF-31
GAAAAAAAATGCGCCATCTAGAAAATTTTTGTCTGTTGC pEryF-29
GAAAAAAAATGCGCCATCTAGAAAATTAATGTCTGTTGC pEryF-27
GAAAAAAAATGCGCCATCTAGAAAAAAAATGTCTGTTGC pEryF-25
GAAAAAAAATGCGCCATCTAGAATTAAAATGTCTGTTGC pEryF-23
GAAAAAAAATGCGCCATCTAGTTTTAAAATGTCTGTTGC pEryF-21
b
E. coli Mach1-T1
?
J23106
B0034
eryD
B0015
p15A, AmpR
pEryF-xx
B0034
sfGFP-His
B0015
ColE1, CmR
c
pEryF-xx-B0034-sfGFP-His
NC
39
37
35
33
31
J23106-B0034-eryD
- + - + - + - + - +
pEryF-xx-B0034-sfGFP-His
NC
27
25
23
21
J23106-B0034-eryD
- + - + - + - +
pEryF-xx-B0034-sfGFP-His
NC
31
29
27
25
J23106-B0034-eryD
- + - + - + - +
d
(kDa) M
250
150
100
70
50
40
35
25
20
pEryF-xx-B0034-sfGFP-His
NC
37
35
33
31
29
J23106-B0034-eryD
- + - + - + - + - +
(kDa) M
250
150
100
70
50
40
35
25
20
pEryF-xx-B0034-sfGFP-His
NC
31
29
27
25
J23106-B0034-eryD
- + - + - + - +

Supplementary Figure 19. The second step for characterization of eryD binding site suffix.
(**a**, **b**) Schematic characterization process of putative eryD binding site (eryO).
(**c**) *E. coli* Mach1-T1 pellets were collected from 1.5 mL overnight LB culture (37°C, 250 rpm for 16 h). Brighter pellets indicated more sfGFP expression. "NC" represents *E. coli* Mach1-T1 without plasmids, and the "-/+" means the *E. coli* strains without or with plasmid pFB261. The result showed that the suffix of eryO was located between pEryF-29 and pEryF-27. Fluorescence images were generated by UVP ChemStudio (analytikjena).
(**d**) Western-Blot analysis of sfGFP (6xHis) relative expression level, the samples were the same as (**c**), the cell pellets were resuspended (equal volume) with 1x phosphate-buffered saline (pH 7.4) and lysed by sonication, the total cell lysate was analyzed.

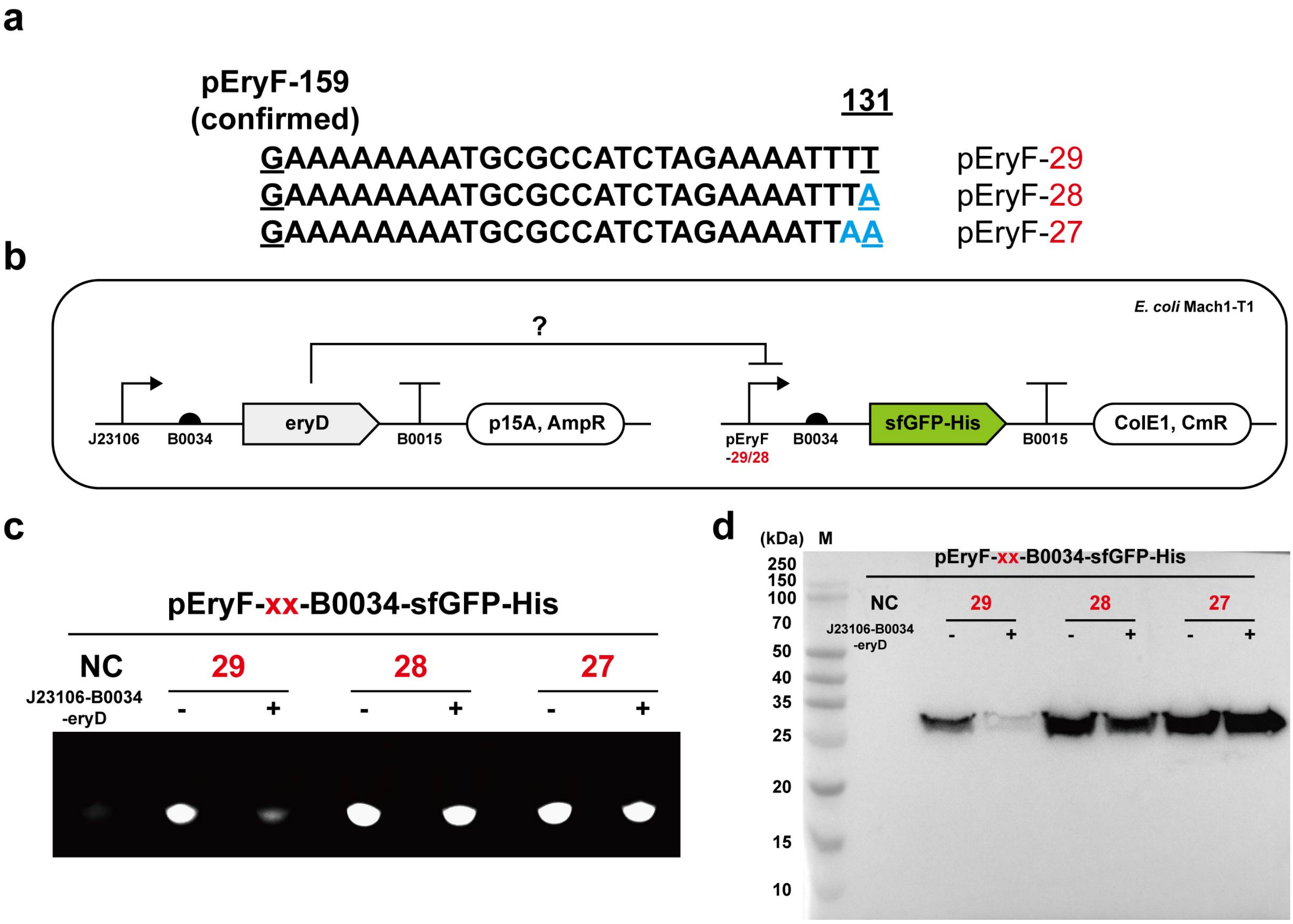

Supplementary Figure 20. The third step for characterization of eryD binding site suffix.

(**a**, **b**) Schematic characterization process of putative eryD binding site (eryO).

(**c**) *E. coli* Mach1-T1 pellets were collected from 1.5 mL overnight LB culture (37°C, 250 rpm for 16 h). Brighter pellets indicated more sfGFP expression. "NC" represents *E. coli* Mach1-T1 without plasmids, and the "-/+" means the *E. coli* strains without or with plasmid pFB261. The result showed that the suffix of eryO was located in pEryF-29 (5'-…AAAATTTT-3'), and the characterized eryO sequence was 5'-GAAAAAAAATGCGCCATCTAGAAAATTTT-3'. Fluorescence images were generated by UVP ChemStudio (analytikjena).

(**d**) Western-Blot analysis of sfGFP (6xHis) relative expression level, the samples were the same as (**c**), the cell pellets were resuspended (equal volume) with 1x phosphate-buffered saline (pH 7.4) and lysed by sonication, the total cell lysate was analyzed.

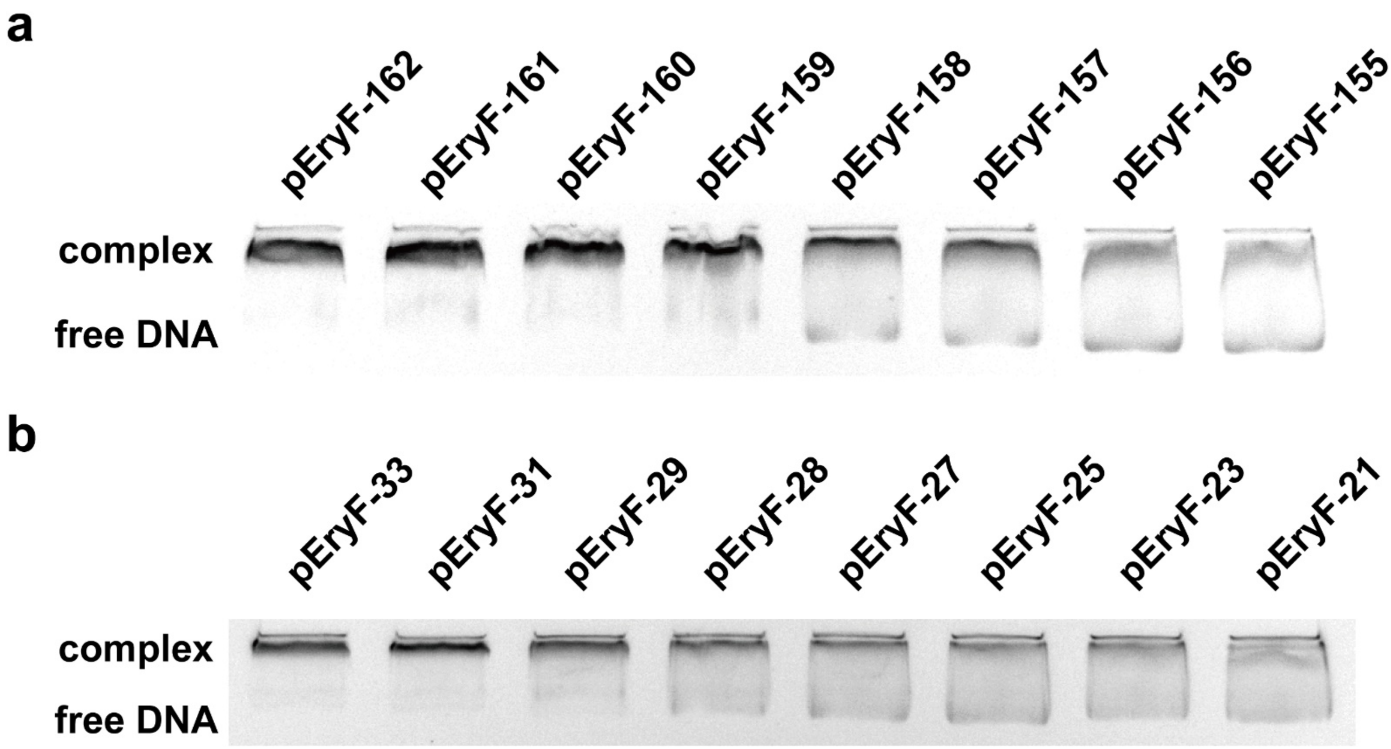

Supplementary Figure 21. EMSA analysis of eryD-eryO interaction.

(**a**) EMSA analysis demonstrates that the prefix of eryO is located in pEryF-159 (5'-GAAAAAAAAT…-3') as the same as *in vivo* experiment results. Each 20 µL reaction consists of 100 nM 5'FAM-labelled pEryF variants and 400 nM purified eryD.

(**b**) EMSA analysis demonstrates that the suffix of eryO is located in pEryF-29 (5'-…AAAATTTT-3') as the same as *in vivo* experiment results. Each 20 µL reaction consists of 100 nM 5'FAM-labelled pEryF variants and 400 nM purified eryD.

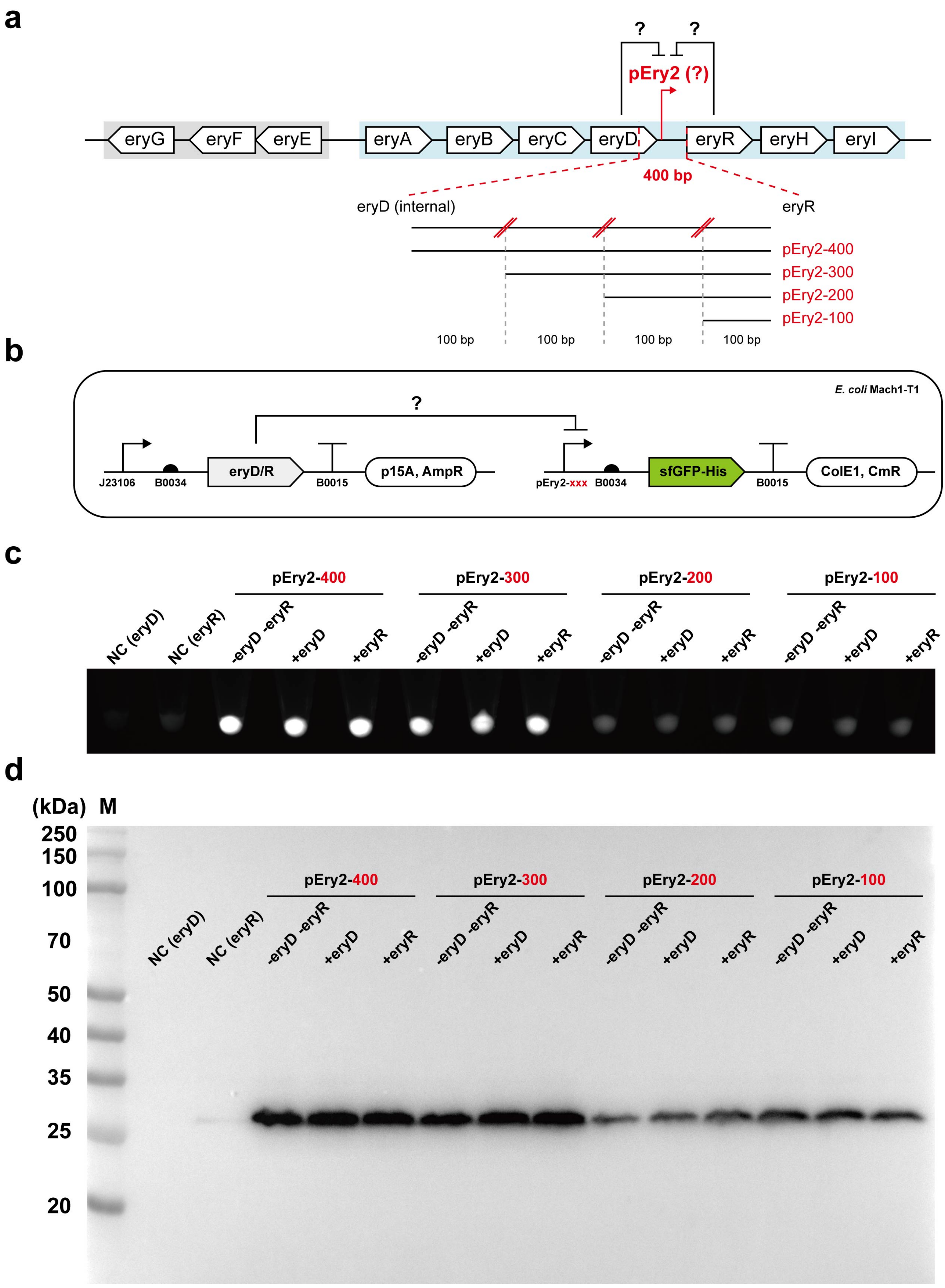
a
?
?
pEry2 (?)
eryG
eryF
eryE
eryA
eryB
eryC
eryD
eryR
eryH
eryI
400 bp
eryD (internal)
eryR
pEry2-400
pEry2-300
pEry2-200
pEry2-100
100 bp
100 bp
100 bp
100 bp
b
E. coli Mach1-T1
?
J23106
B0034
eryD/R
B0015
p15A, AmpR
pEry2-xxx
B0034
sfGFP-His
B0015
ColE1, CmR
c
pEry2-400
pEry2-300
pEry2-200
pEry2-100
NC (eryD)
NC (eryR)
-eryD -eryR
+eryD
+eryR
d
(kDa)
M
250
150
100
70
50
40
35
25
20

Supplementary Figure 22. pEry2 characterization.
(**a**) Schematic characterization process of whether there were eryD/eryR binding sites in the region of pEry2 variants. We assumed that eryD/eryR might regulate the erythritol cluster, and the long DNA gap between eryD and eryR might locate eryD/eryR binding sites in both forward and reverse directions.
(**b**) Schematic compatible plasmids design in *E. coli* Mach1-T1. Plasmids that could strongly express eryD/eryR (pFB261 and pFB266) co-existed with the reporter plasmids, which contained 100 bp truncated pEry2 variants (pFB256 to pFB259).
(**c**) *E. coli* Mach1-T1 pellets were collected from 1.5 mL overnight LB culture (37°C, 250 rpm for 16 h). Brighter pellets indicated more sfGFP expression. Two "NC" are the same and represent *E. coli* Mach1-T1 without plasmids, and the "-/+" means the *E. coli* strains without or with plasmid pFB261 or pFB266. The result showed that there were not any eryD or eryR DNA binding sites in pEry2 region. Fluorescence images were generated by UVP ChemStudio (analytikjena).
(**d**) Western-Blot analysis of sfGFP (6xHis) relative expression level, the samples were the same as (**c**), the cell pellets were resuspended (equal volume) with 1x phosphate-buffered saline (pH 7.4) and lysed by sonication, the total cell lysate was analyzed.

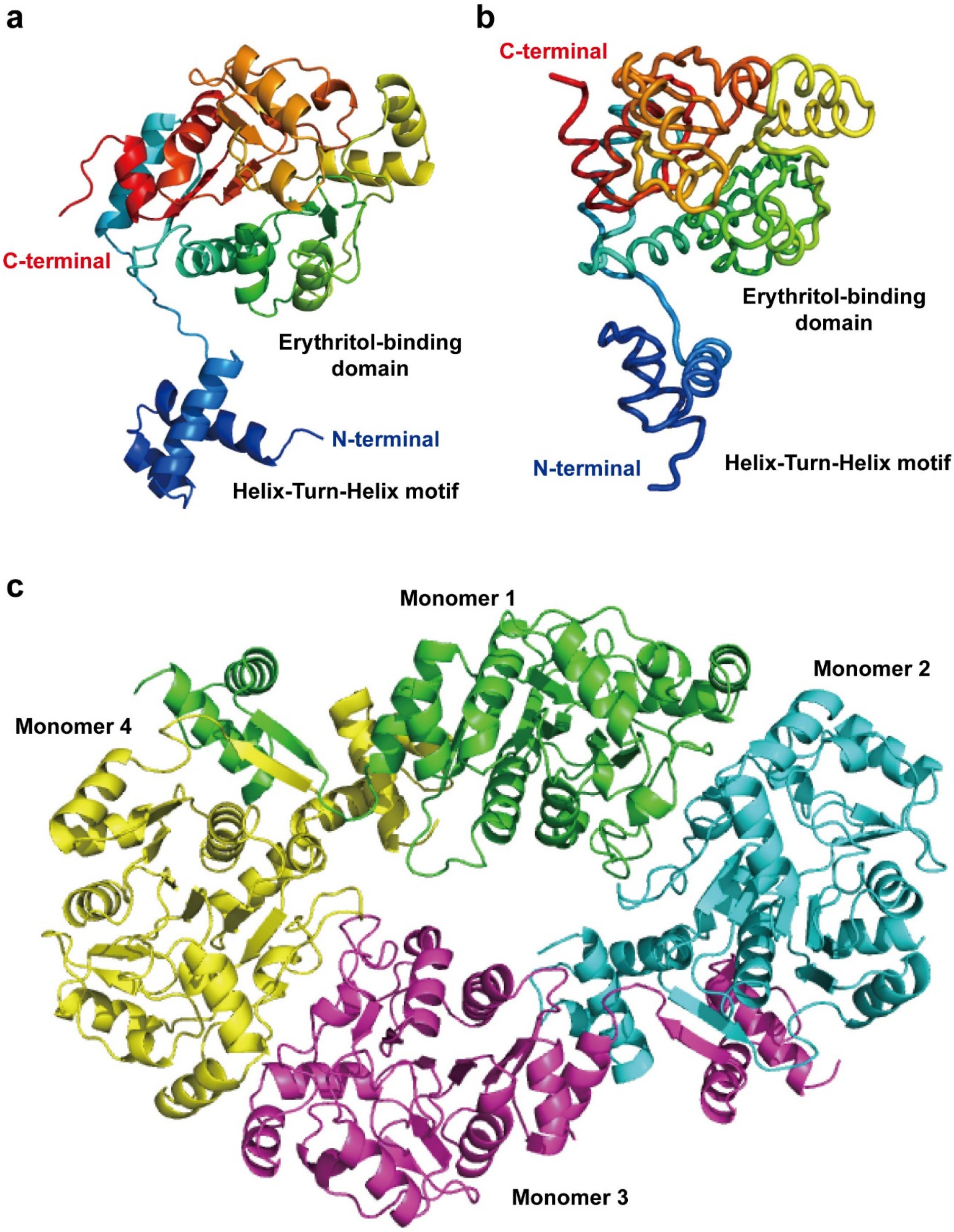

Supplementary Figure 23. Structure models of eryD monomer and homotetramer.
(**a**) AlphaFold[8,9] structure prediction of eryD monomer (Uniprot ID: Q2YIQ4). This predicted eryD is from *Brucella abortus* (strain 2308), and the amino acid sequence is 100% the same as the eryD we characterized in this work.
(**b**) Robetta[10] structure prediction of eryD that we characterized in this work.
(**c**) SWISS-MODEL[11,12] homotetramer structure prediction of eryD that we characterized in this work. This predicted model is built up with the PDB template 4l5i.1.A (Transcriptional regulator LsrR).

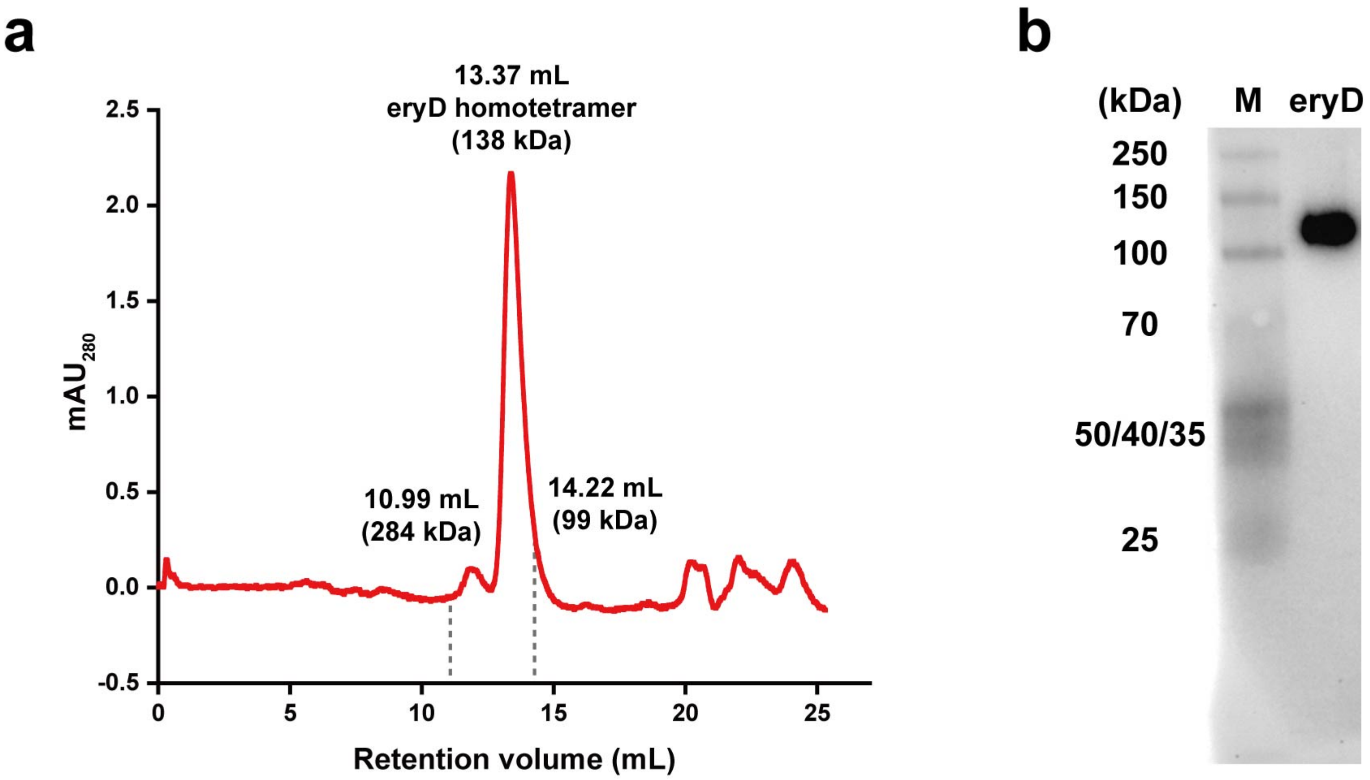

Supplementary Figure 24. eryD assembled as a homotetramer.

(**a**) Size exclusion chromatography of the purified eryD (6xHis) with Superdex 200 increase 10/300 GL column. eryD homotetramer was eluted at 13.37 mL (red peak). The other two purified proteins were used as standards: Vlm2 (284 kDa) was eluted at 10.99 mL and T7 RNA polymerase (99 kDa) was eluted at 14.22 mL. (**b**) Native-PAGE based Western-Blot analysis of the purified eryD (6xHis). The expected homotetramer band (approximately 138 kDa) was shown between the two protein markers of 100 kDa and 150 kDa.

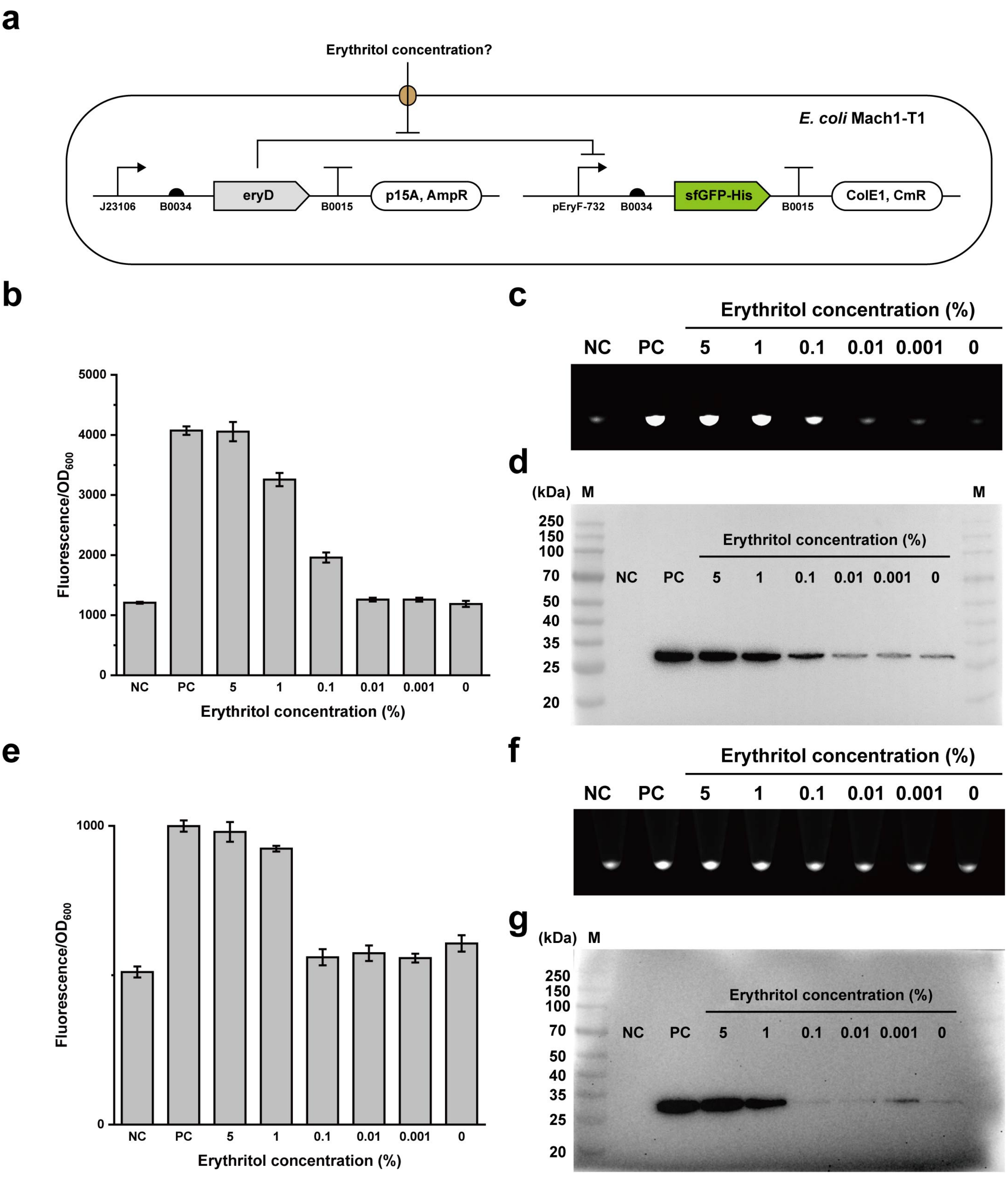

Supplementary Figure 25. Erythritol induction of pEryF-732 at 30°C and 16°C.

(**a**) Schematic diagram of used strain and plasmids. pFB186 and pFB261 co-existed in *E. coli* Mach1-T1.

(**b**) The mentioned strain in (**a**) was induced by different concentrations of erythritol during overnight incubation in LB medium at 30°C, 250 rpm for 16 h. “NC” represents *E. coli* Mach1-T1 without plasmids, “PC” represents *E. coli*

Mach1-T1 with plasmid pFB186 and without pFB261. All measurements were performed with three biological replicates.
(**c**) The collected *E. coli* Mach1-T1 pellets from (**b**), brighter pellets indicated more sfGFP expression.
(**d**) Western-Blot analysis of sfGFP (6xHis) relative expression level, the samples were the same as (**b**) and (**c**), the cell pellets were resuspended (equal volume) with 1x phosphate-buffered saline (pH 7.4) and lysed by sonication, the total cell lysate was analyzed.
(**e**) The mentioned strain in (**a**) was induced by different concentrations of erythritol during overnight incubation in LB medium at 16°C, 250 rpm for 24 h. “NC” represents *E. coli* Mach1-T1 without plasmids, “PC” represents *E. coli* Mach1-T1 with plasmid pFB186 and without pFB261. All measurements were performed with three biological replicates.
(**f**) The collected *E. coli* Mach1-T1 pellets from (**e**), brighter pellets indicated more sfGFP expression.
(**g**) Western-Blot analysis of sfGFP (6xHis) relative expression level, the samples were the same as (**e**) and (**f**), the cell pellets were resuspended (equal volume) with 1x phosphate-buffered saline (pH 7.4) and lysed by sonication, the total cell lysate was analyzed.

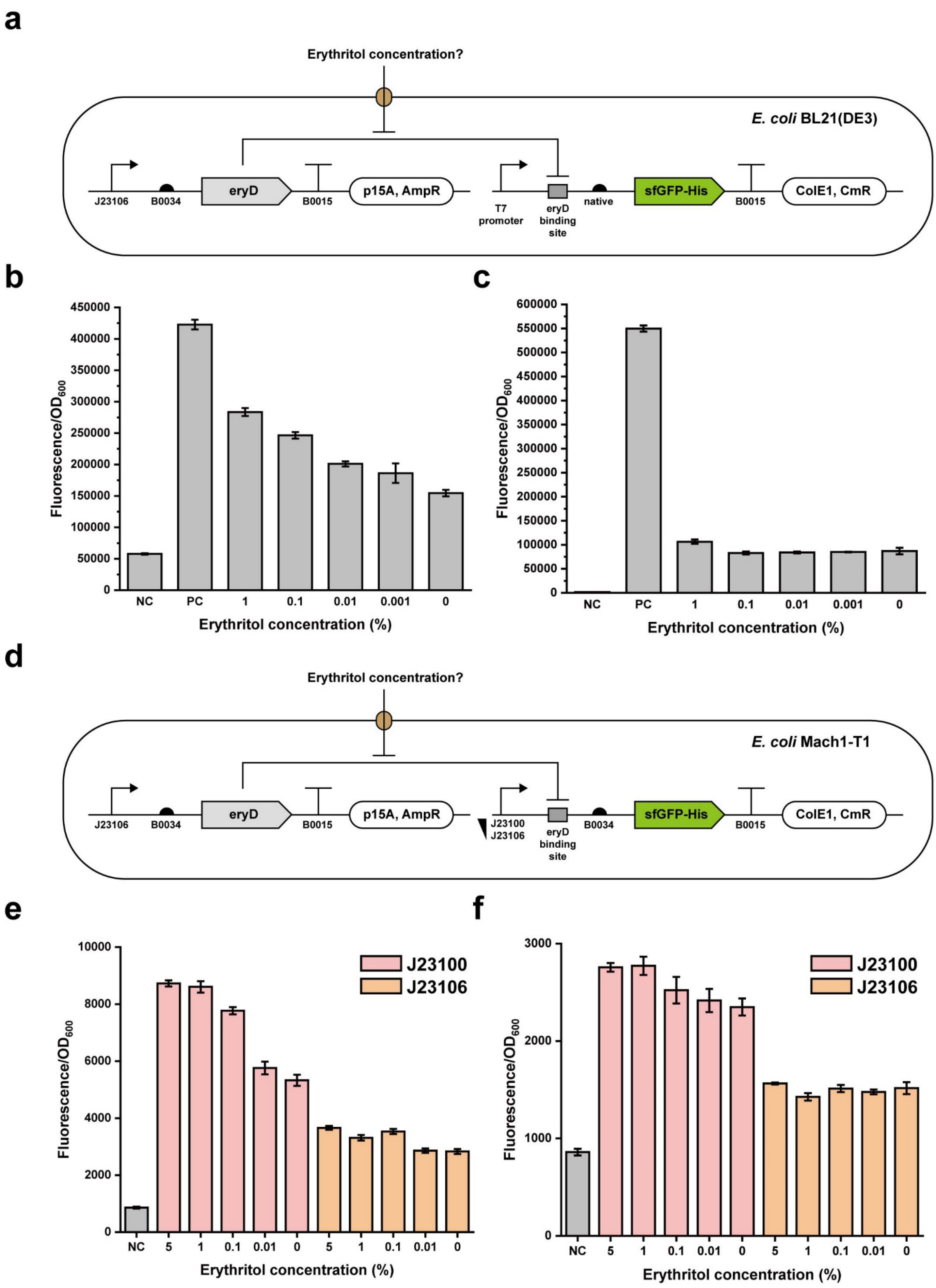
a
Erythritol concentration?
E. coli BL21(DE3)
J23106
B0034
eryD
B0015
p15A, AmpR
T7 promoter
eryD binding site
native
sfGFP-His
B0015
ColE1, CmR
b
Fluorescence/OD600
Erythritol concentration (%)
NC
PC
1
0.1
0.01
0.001
0
c
Fluorescence/OD600
Erythritol concentration (%)
NC
PC
1
0.1
0.01
0.001
0
d
Erythritol concentration?
E. coli Mach1-T1
J23106
B0034
eryD
B0015
p15A, AmpR
J23100
J23106
eryD binding site
B0034
sfGFP-His
B0015
ColE1, CmR
e
J23100
J23106
Fluorescence/OD600
Erythritol concentration (%)
NC
5
1
0.1
0.01
0
f
J23100
J23106
Fluorescence/OD600
Erythritol concentration (%)
NC
5
1
0.1
0.01
0

Supplementary Figure 26. Erythritol induction of synthetic operons at 30°C and 16°C.

(**a**) Schematic diagram of used strain and plasmids for pT7-eryO operon characterization. pFB276 and pFB261 co-existed in *E. coli* BL21(DE3).

(**b**) The mentioned strain in (**a**) was induced by different concentrations of erythritol during overnight incubation in LB medium (with 0.5 mM IPTG for T7 RNA polymerase induction) at 30°C, 250 rpm for 16 h. "NC" represents the mentioned strain without IPTG and without erythritol induction, "PC" represents *E. coli* BL21(DE3) with plasmid pFB277 (with 0.5 mM IPTG for T7 RNA polymerase induction and without erythritol induction). All measurements were performed with three biological replicates.

(**c**) The mentioned strain in (**a**) was induced by different concentrations of erythritol during overnight incubation in LB medium (with 0.5 mM IPTG for T7 RNA polymerase induction) at 16°C, 250 rpm for 24 h. "NC" represents the mentioned strain without IPTG and without erythritol induction, "PC" represents *E. coli* BL21(DE3) with plasmid pFB277 (with 0.5 mM IPTG for T7 RNA polymerase induction and without erythritol induction). All measurements were performed with three biological replicates.

(**d**) Schematic diagram of used strain and plasmids for J23100-eryO/J23106-eryO operon characterization. pFB270 and pFB261/pFB272 and pFB261 co-existed in *E. coli* Mach1-T1.

(**e**) The mentioned strains in (**d**) were induced by different concentrations of erythritol during overnight incubation in LB medium at 30°C, 250 rpm for 16 h. "NC" represents *E. coli* Mach1-T1 without plasmids. All measurements were performed with three biological replicates.

(**f**) The mentioned strains in (**d**) were induced by different concentrations of erythritol during overnight incubation in LB medium at 16°C, 250 rpm for 24 h. "NC" represents *E. coli* Mach1-T1 without plasmids. All measurements were performed with three biological replicates.

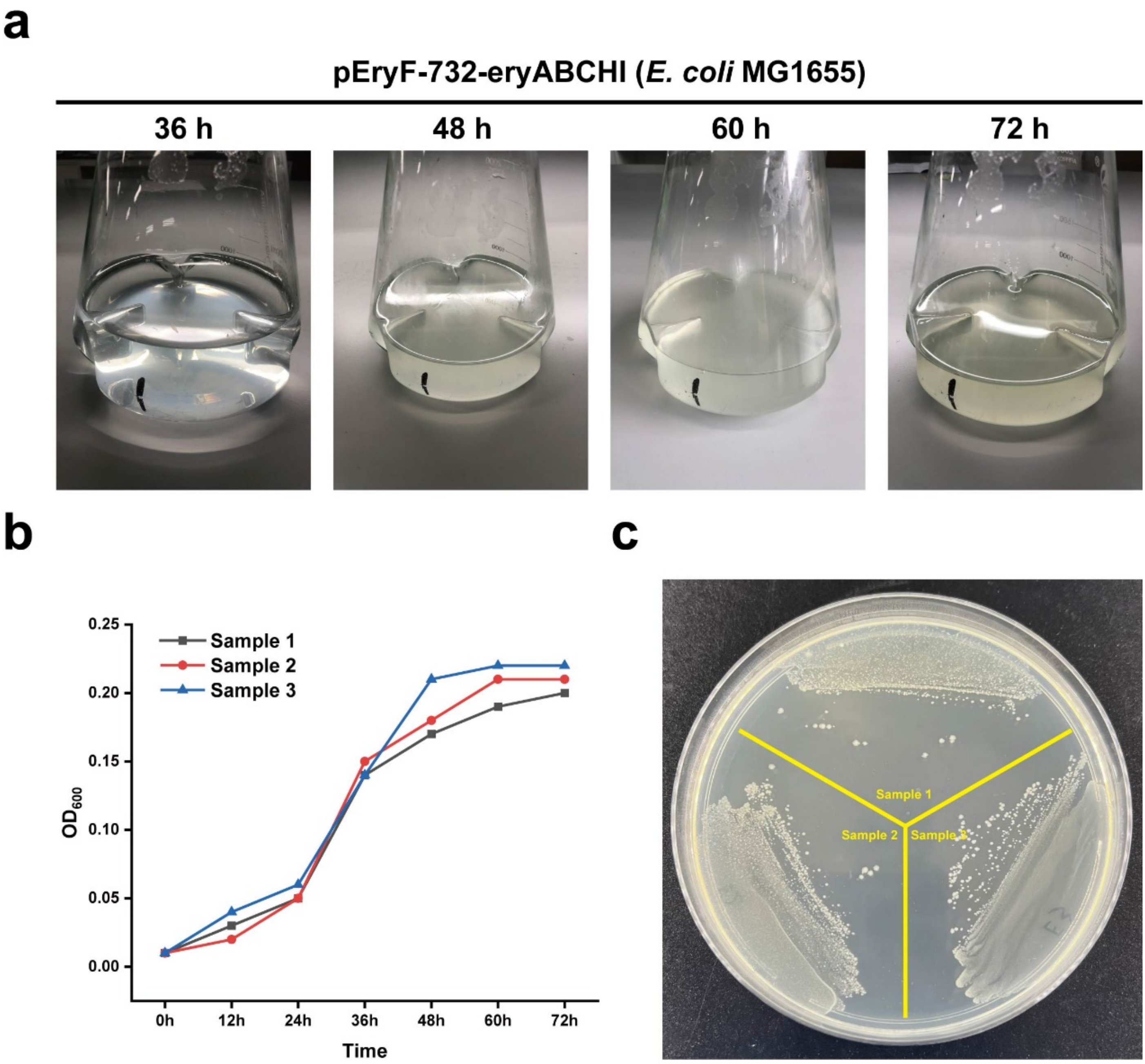

Supplementary Figure 27. Sample preparation for mRNA transcriptional analysis.
Wild-type *E. coli* MG1655 harboring plasmid pFB147 was incubated in 5 mL LB medium (with 34 μg/mL chloramphenicol) for overnight cultivation (37°C and 250 rpm for 16 h). On the second day, 5 mL cell culture was washed with 1x phosphate-buffered saline (pH 7.4) by three times and then resuspended by 5 mL M9-erythritol liquid medium. Then, the resuspended cell mixture was inoculated into 1 L M9-erythritol liquid medium (with 6 μg/mL chloramphenicol) as 1:200 v/v in 2 L shaking flask. After that, the cells were incubated for 72 h (37°C and 250 rpm). Each cultivation was repeated with three biological replicates. The cells after 36 h cultivation were collected to prepare RNA-seq samples. (**a**) The cell cultures in pre-experiments reached stationary phase at 72 h. (**b**) Cell growth curves of pre-experiment samples. The middle logarithmic phase reached at 36 h and the stationary phase reached at 72 h. (**c**) The cell cultures (samples in pre-experiment at 72 h) were picked and spread on LB-agar plates (with 34 μg/mL chloramphenicol) to test the cell viability (overnight cultivation on the plate for 16 h at 37°C). The colonies were normal and did not lose the plasmid pFB147.

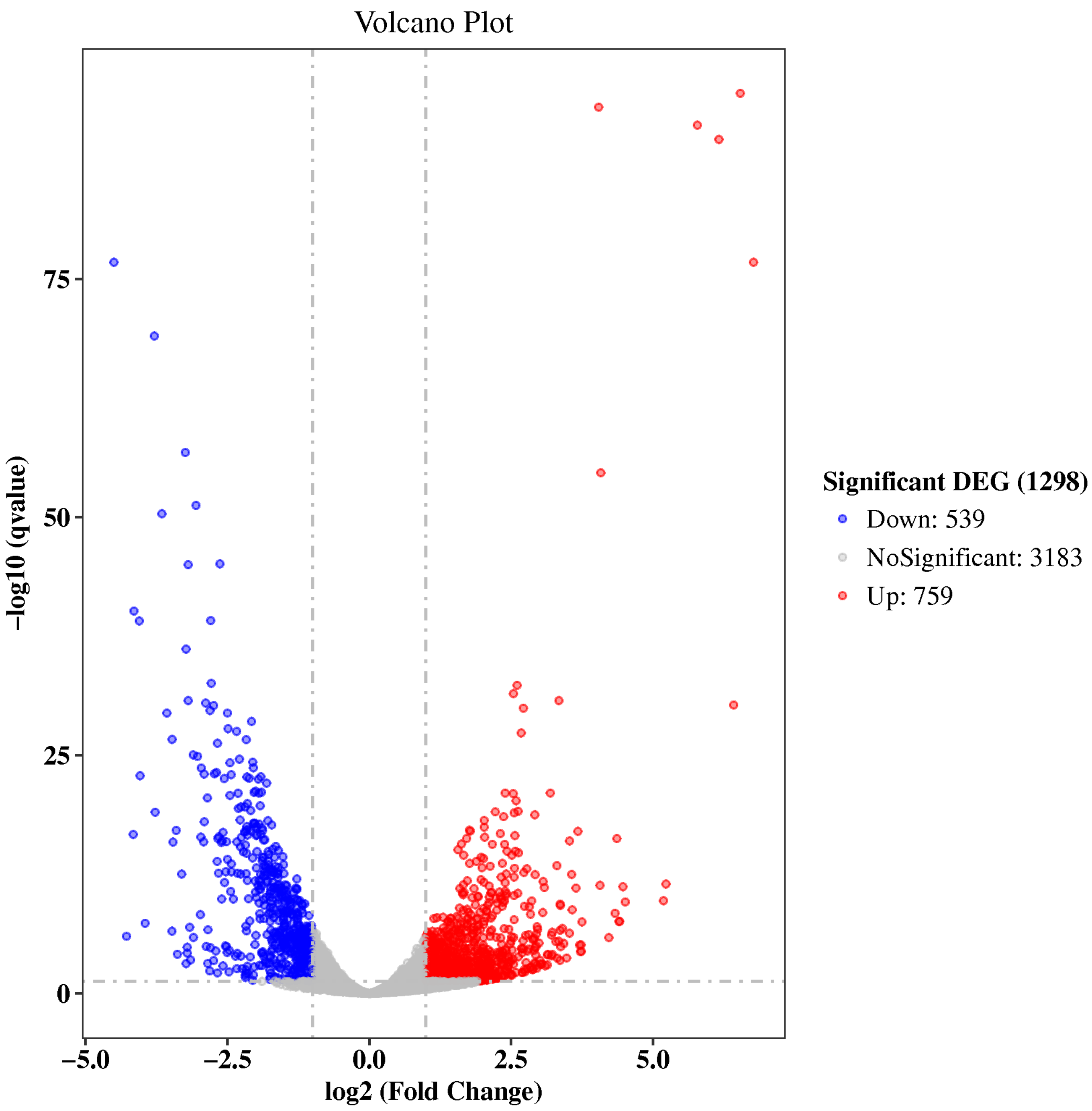

Supplementary Figure 28. Volcano plot of differentially expressed genes from mRNA transcriptional analysis. Volcano plot depicts differentially expressed genes between M9-glucose and M9-erythritol mediums (n=3 as independent biological replicates). The genes of more than two times or less than two times (q value ≤ 0.05) were recognized as differentially expressed.

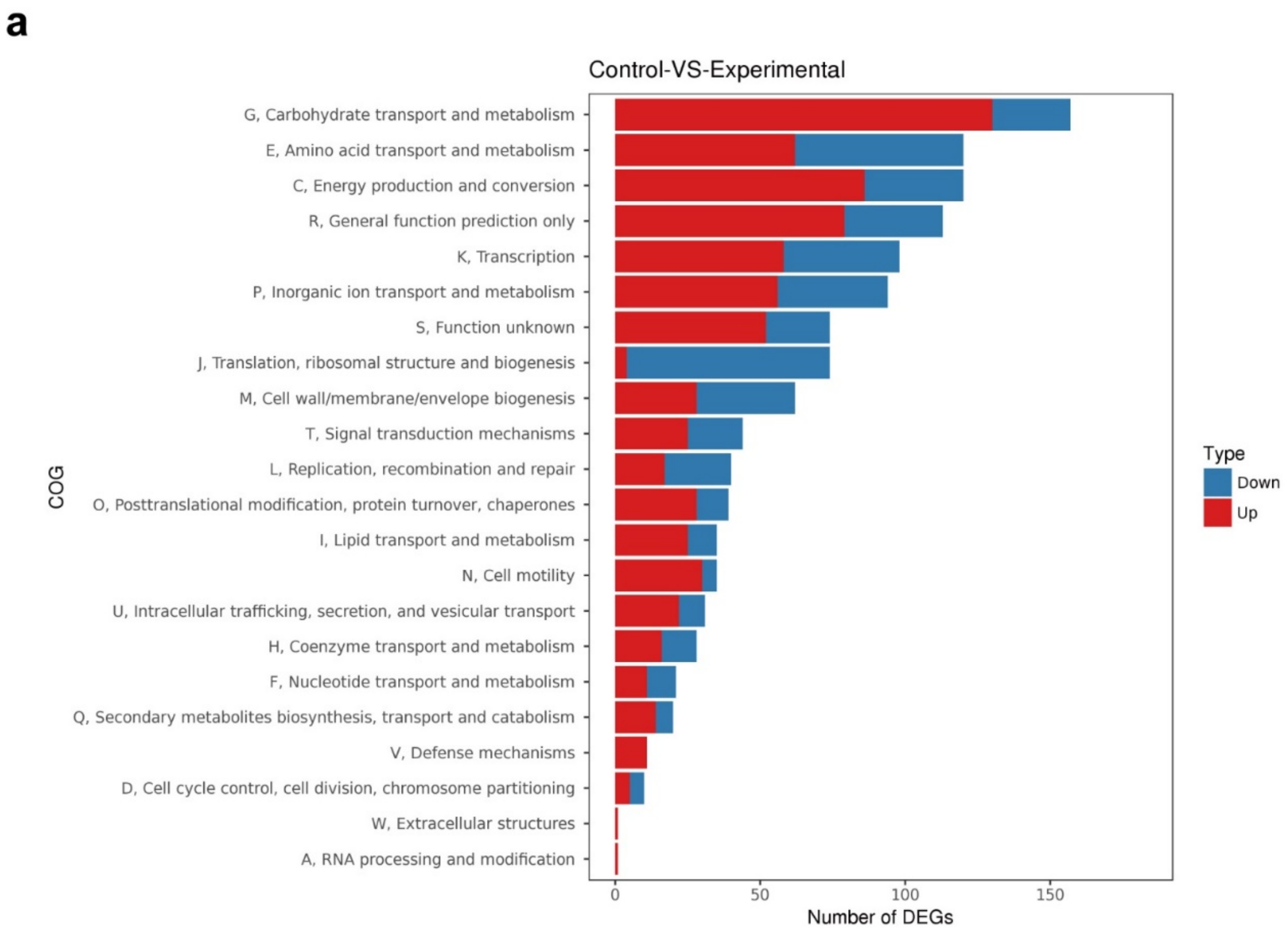
a
Control-VS-Experimental
G, Carbohydrate transport and metabolism
E, Amino acid transport and metabolism
C, Energy production and conversion
R, General function prediction only
K, Transcription
P, Inorganic ion transport and metabolism
S, Function unknown
J, Translation, ribosomal structure and biogenesis
M, Cell wall/membrane/envelope biogenesis
T, Signal transduction mechanisms
L, Replication, recombination and repair
O, Posttranslational modification, protein turnover, chaperones
I, Lipid transport and metabolism
N, Cell motility
U, Intracellular trafficking, secretion, and vesicular transport
H, Coenzyme transport and metabolism
F, Nucleotide transport and metabolism
Q, Secondary metabolites biosynthesis, transport and catabolism
V, Defense mechanisms
D, Cell cycle control, cell division, chromosome partitioning
W, Extracellular structures
A, RNA processing and modification
COG
0
50
100
150
Number of DEGs
Type
Down
Up

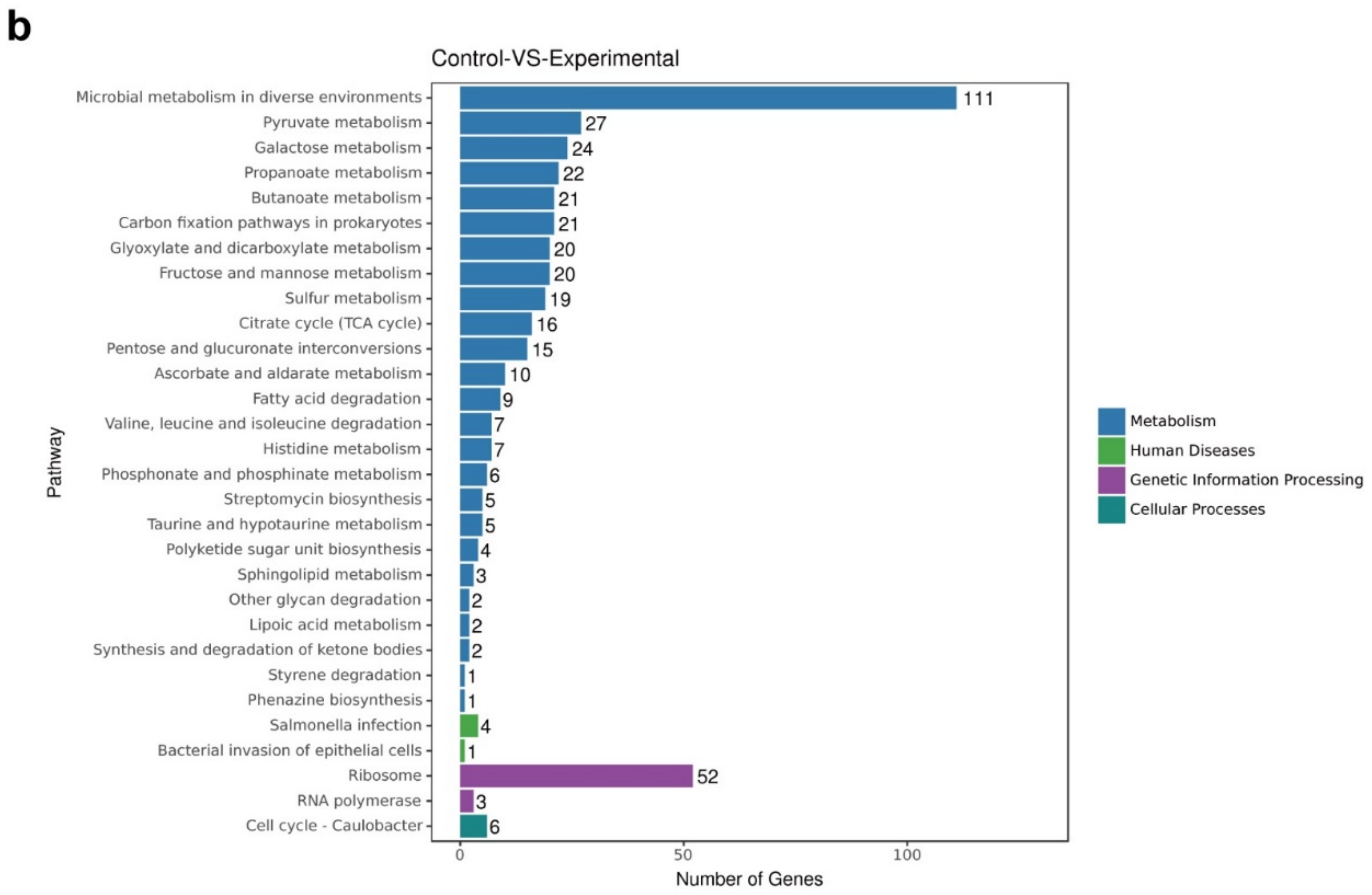
b
Control-VS-Experimental
Microbial metabolism in diverse environments
111
Pyruvate metabolism
27
Galactose metabolism
24
Propanoate metabolism
22
Butanoate metabolism
21
Carbon fixation pathways in prokaryotes
21
Glyoxylate and dicarboxylate metabolism
20
Fructose and mannose metabolism
20
Sulfur metabolism
19
Citrate cycle (TCA cycle)
16
Pentose and glucuronate interconversions
15
Ascorbate and aldarate metabolism
10
Fatty acid degradation
9
Valine, leucine and isoleucine degradation
7
Histidine metabolism
7
Phosphonate and phosphinate metabolism
6
Streptomycin biosynthesis
5
Taurine and hypotaurine metabolism
5
Polyketide sugar unit biosynthesis
4
Sphingolipid metabolism
3
Other glycan degradation
2
Lipoic acid metabolism
2
Synthesis and degradation of ketone bodies
2
Styrene degradation
1
Phenazine biosynthesis
1
Salmonella infection
4
Bacterial invasion of epithelial cells
1
Ribosome
52
RNA polymerase
3
Cell cycle - Caulobacter
6
Pathway
0
50
100
Number of Genes
Metabolism
Human Diseases
Genetic Information Processing
Cellular Processes

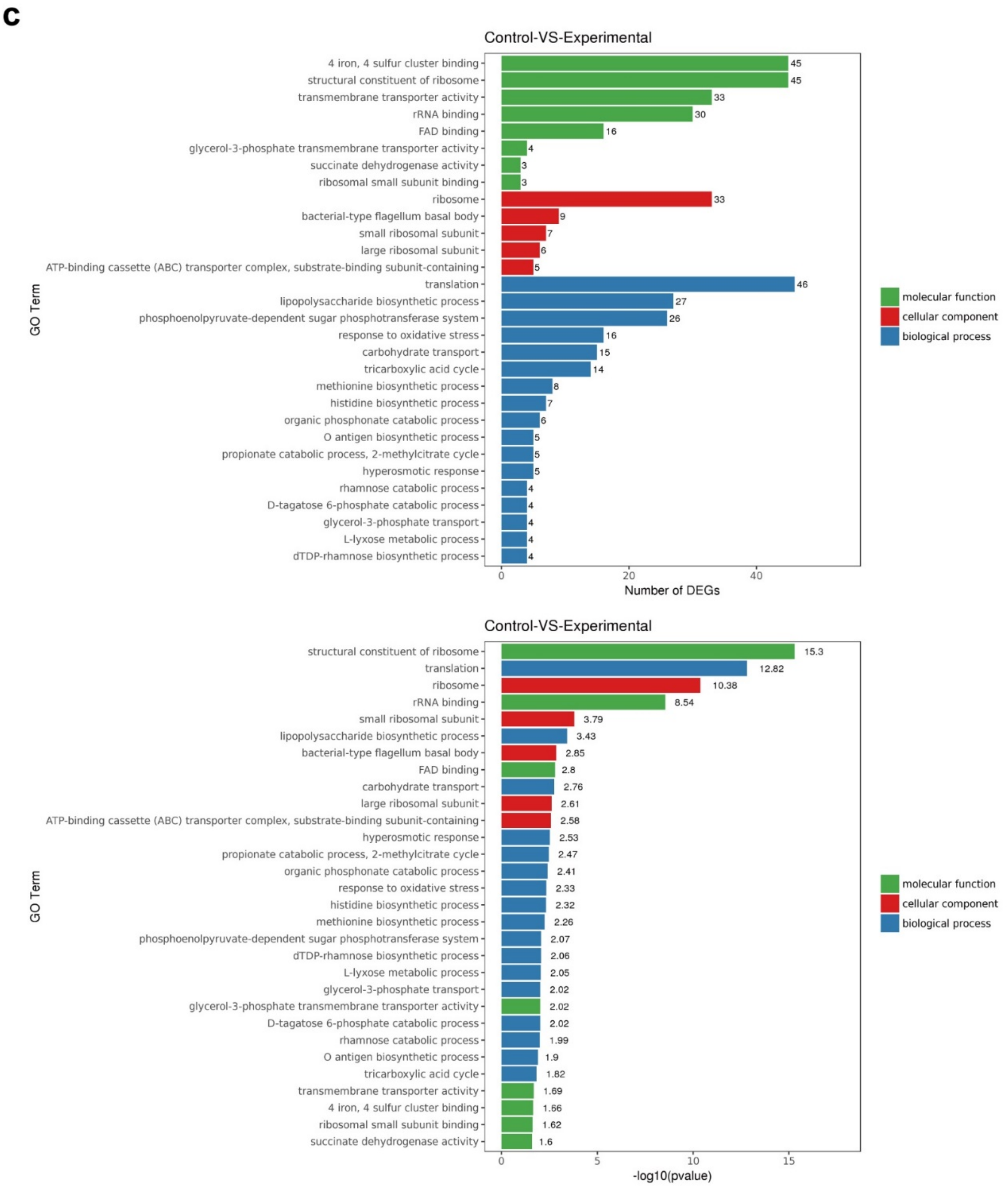

Supplementary Figure 29. mRNA transcriptional analysis.

Transcriptome results were analyzed via (**a**) COG, (**b**) KEGG, and (**c**) GO databases. The shared different genes were associated with carbohydrate metabolism, amino acid metabolism, and ribosome. The genes of carbohydrate metabolism were up-regulated; however, ribosome and translation related genes were down-regulated.

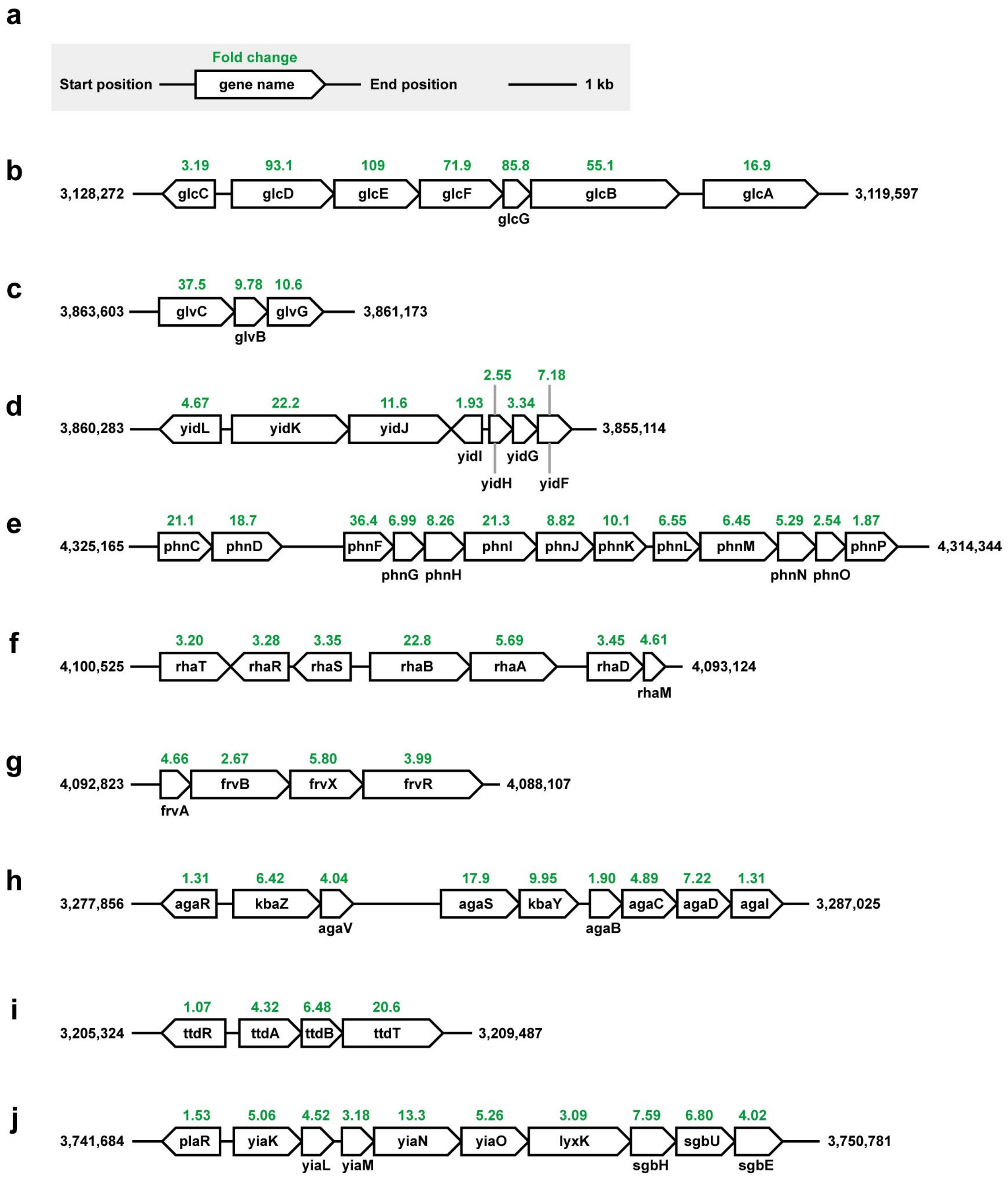

Supplementary Figure 30. Some up-regulated gene clusters (RNA-seq) from M9-erythritol catabolic *E. coli* strain.

E. coli MG1655 genome sequence is used as a reference (GenBank: U00096.3)[13].

(**a**) Schematic diagram.

(**b**) Glycolate utilization cluster.

(**c**) *glv* operon as a putative PTS system.

(**d**) *yid* operon.

(**e**) *phn* operon as phosphonate uptake and utilization.

(**f**) *rha* operon as L-rhamnose uptake and utilization.

(**g**) *frv* operon as a putative PTS system.

(**h**) *aga* operon as N-acetylgalactosamine uptake and utilization.

(**i**) *ttd* operon as L-tartrate uptake and utilization.

(**j**) *yia-sgb* operon.

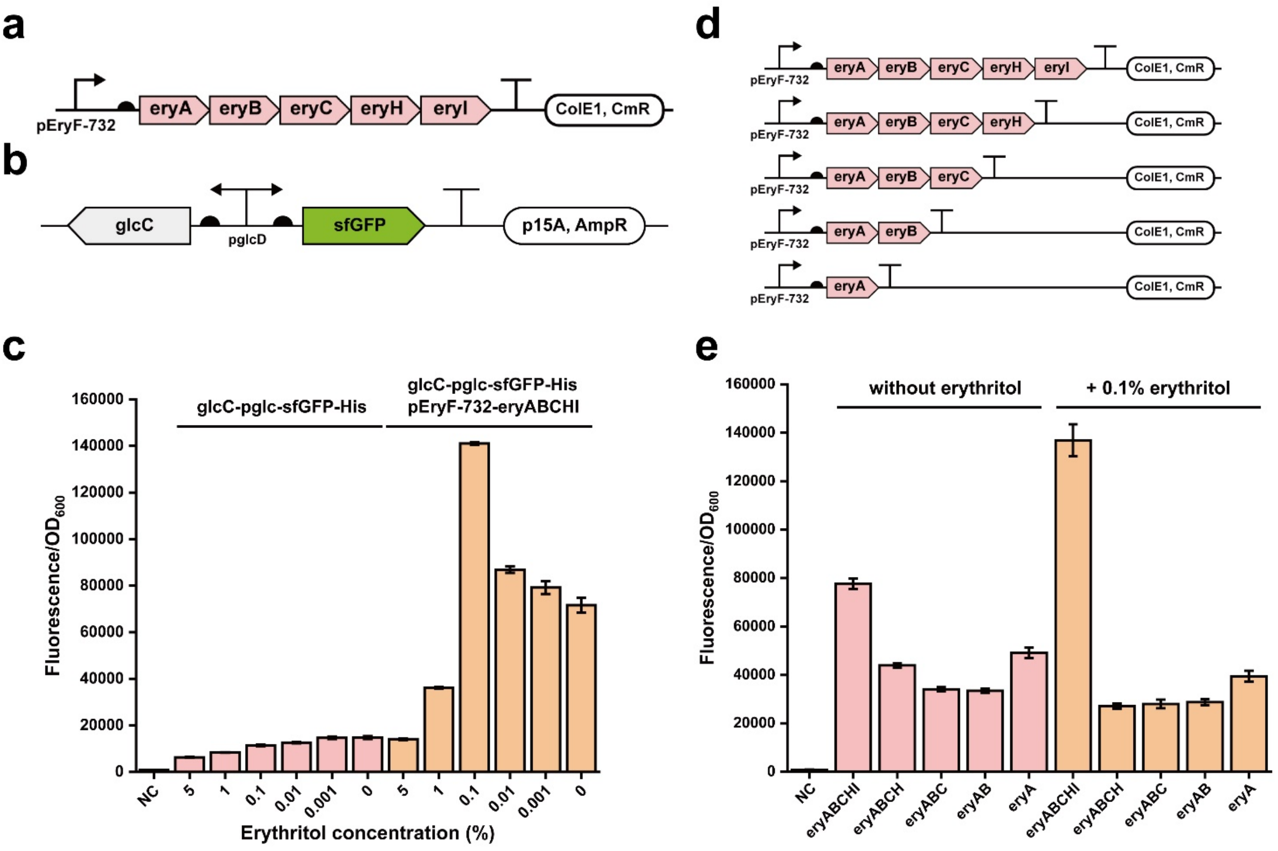

Supplementary Figure 31. glcC can respond to erythritol catabolism.

(**a**) Schematic of erythritol catabolism plasmid pFB147.

(**b**) glcC and putative glcC regulated promoter region pglcD were assembled with sfGFP as a reporter (plasmid pFB340).

(**c**) *E. coli* Mach1-T1 with plasmid pFB340 or pFB340 + pFB147 were incubated overnight with different concentrations of erythritol in 5 mL LB medium at 37°C and 250 rpm for 16 h. sfGFP fluorescence was measured with standardization. The results showed that with optimal 0.1% erythritol, glcC could well respond to erythritol catabolism-related molecules.

(**d**) A series of truncated erythritol catabolism plasmids (pFB147, pFB153 to pFB156) were constructed to determine whether glcC could respond to erythritol catabolism-related molecules.

(**e**) pFB340 co-existed with the truncated plasmids in *E. coli* Mach1-T1. Such strains were incubated overnight with or without 0.1% erythritol in 5 mL LB medium at 37°C and 250 rpm for 16 h. sfGFP fluorescence was measured with standardization. The results showed that glcC-responded substrate was not erythritol catabolism-related molecules.

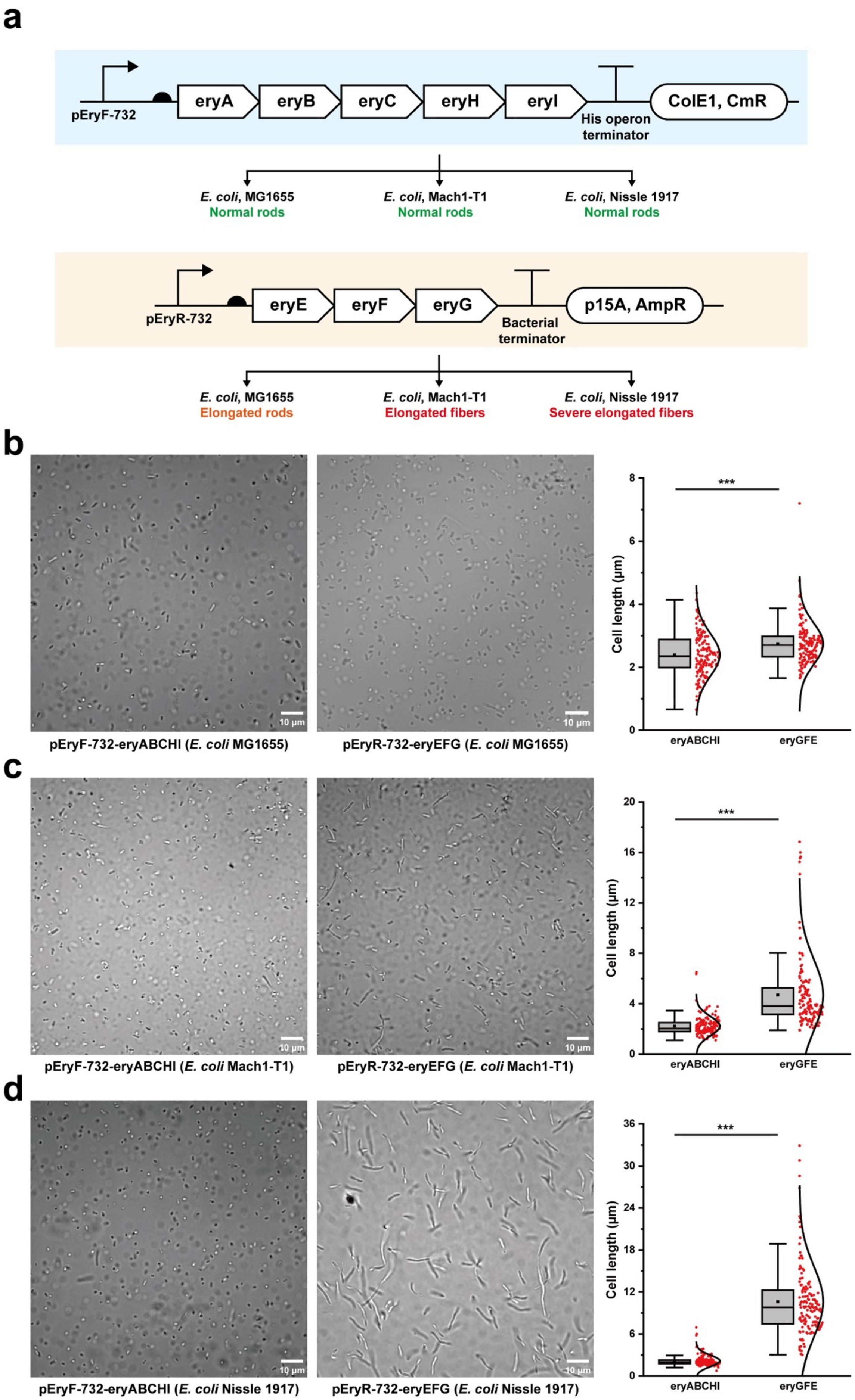
a
pEryF-732
eryA
eryB
eryC
eryH
eryI
His operon terminator
ColE1, CmR
E. coli, MG1655
Normal rods
E. coli, Mach1-T1
Normal rods
E. coli, Nissle 1917
Normal rods
pEryR-732
eryE
eryF
eryG
Bacterial terminator
p15A, AmpR
E. coli, MG1655
Elongated rods
E. coli, Mach1-T1
Elongated fibers
E. coli, Nissle 1917
Severe elongated fibers
b
10 μm
pEryF-732-eryABCHI (E. coli MG1655)
pEryR-732-eryEFG (E. coli MG1655)
Cell length (μm)
***
eryABCHI
eryGFE
c
pEryF-732-eryABCHI (E. coli Mach1-T1)
pEryR-732-eryEFG (E. coli Mach1-T1)
d
pEryF-732-eryABCHI (E. coli Nissle 1917)
pEryR-732-eryEFG (E. coli Nissle 1917)

Supplementary Figure 32. Erythritol ABC transporter cluster changes *E. coli* morphology.

(**a**) Schematic characterization process of whether erythritol catabolic cluster (pFB147) and erythritol ABC transporter cluster (pFB157) would influence cell morphology in different *E. coli* strains. All cell cultures were incubated in LB medium at 37°C and 250 rpm for 16 h. Note that *E. coli* MG1655 harboring pFB157 could only grow in LB medium with 50 μg/mL ampicillin or lower ampicillin concentrations.

(**b**) Cell morphology of *E. coli* MG1655 (harboring pFB147, left or pFB157, right). Both strains were visualized and cell length was measured statistically using 144 individual cells. Boxplot shows statistical analysis. Student's *t*-tests are used for statistical analysis, and $p < 0.05$ indicates statistical significance (***$p < 0.001$).

(**c**) Cell morphology of *E. coli* Mach1-T1 (harboring pFB147, left or pFB157, right). Both strains were visualized and cell length was measured statistically using 144 individual cells. Boxplot shows statistical analysis. Student's *t*-tests are used for statistical analysis, and $p < 0.05$ indicates statistical significance (***$p < 0.001$).

(**d**) Cell morphology of *E. coli* Nissle 1917 (harboring pFB147, left or pFB157, right). Both strains were visualized and cell length was measured statistically using 144 individual cells. Boxplot shows statistical analysis. Student's *t*-tests are used for statistical analysis, and $p < 0.05$ indicates statistical significance (***$p < 0.001$).

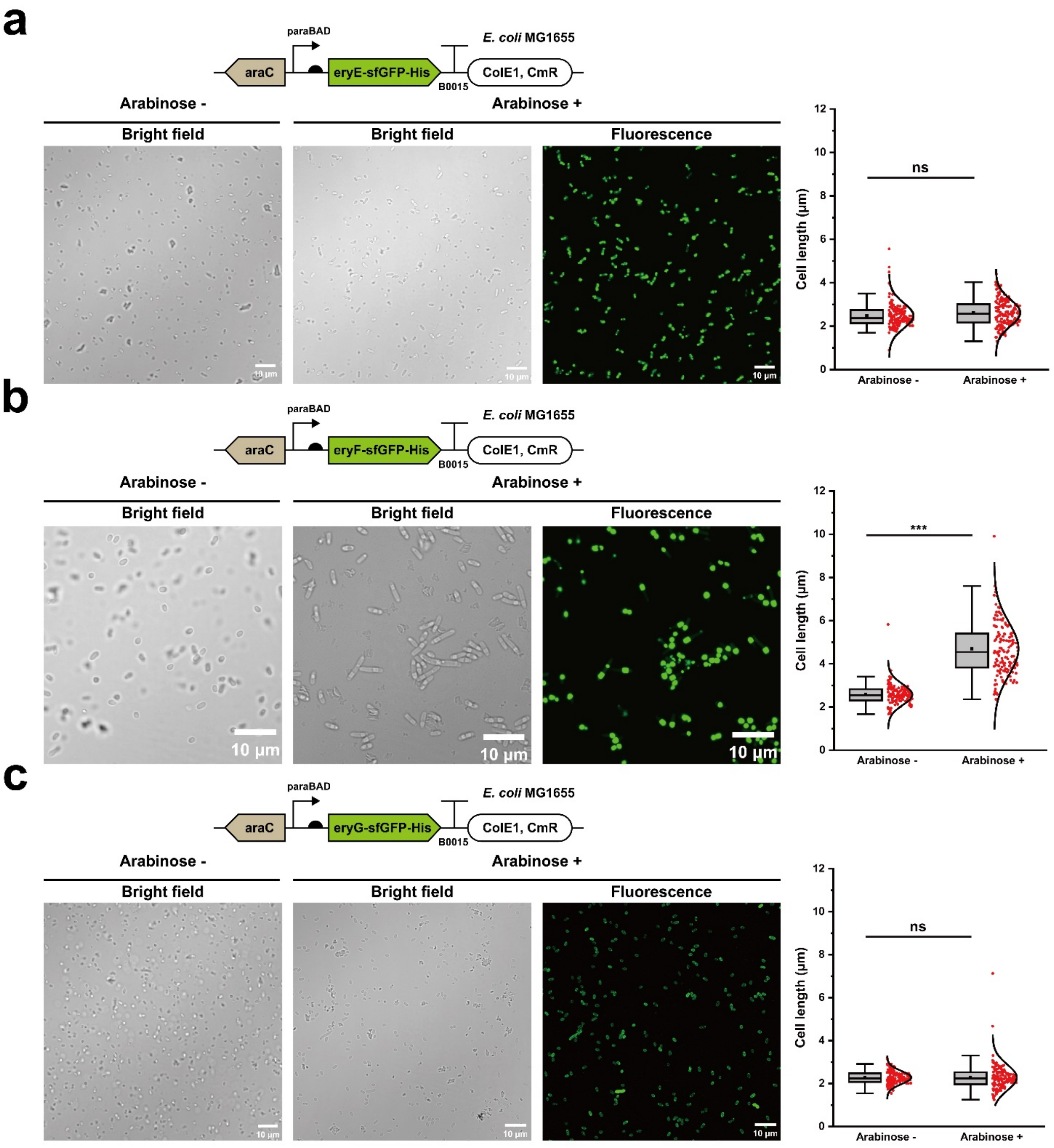

Supplementary Figure 33. Effect of eryE, eryF, and eryG on cell morphology.
E. coli MG1655 harboring the plasmid of (**a**) eryE-sfGFP, (**b**) eryF-sfGFP, or (**c**) eryG-sfGFP was incubated in 5 mL of LB medium at 37°C and 250 rpm. When OD_{600} reached 0.6, 1% arabinose (w/v) was added (or not added) and incubated for another 6 h at 30°C and 250 rpm. Then, cell pellets were resuspended (equal volume) with 1x phosphate-buffered saline (pH 7.4) and imaged under laser scanning confocal microscopy. Cell length was measured statistically using 144 individual cells for each strain. Boxplot shows statistical analysis. Student's *t*-tests are used for statistical analysis, and $p < 0.05$ indicates statistical significance (***$p < 0.001$; ns, $p > 0.05$). The data suggested that eryF was the key component that caused the change of cell morphology.

a

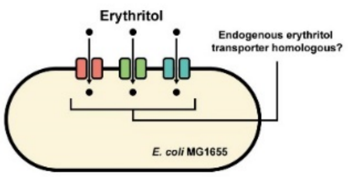

b

C_n	Gene	Protein	Substrate
C_3	glpF	Glycerol uptake facilitator protein	Glycerol
	rbsA	Ribose import ATP-binding protein	
C_5	rbsB	Ribose import binding protein	D-Ribose
	rbsC	Ribose import permease protein	
	araF	L-arabinose-binding periplasmic protein	
C_5	araG	Arabinose import ATP-binding protein	L-arabinose
	araH	L-arabinose transport system permease protein	
C_5	araE	Arabinose-proton symporter	L-arabinose
	xylF	D-xylose-binding periplasmic protein	
C_5	xylG	Xylose import ATP-binding protein	D-xylose
	xylH	Xylose transport system permease protein	
C_5	xylE	D-xylose-proton symporter	D-xylose
C_5/C_6	rhaT	L-rhamnose-proton symporter	Lyxose/L-rhamnose/L-xylose/mannose
	crr	PTS system glucose-specific EIIA component	
C_6	ptsG	PTS system glucose-specific EIICB component	D-glucose
	ptsH	Phosphocarrier protein	
	ptsI	Phosphoenolpyruvate-protein phosphotransferase	
C_6	fruA	PTS system fructose-specific EIIB'BC component	D-frucose
	fruB	Multiphosphoryl transfer protein	
C_6	galP	Galactose-proton symporter	Galactose
	mglA	Galactose/methyl galactoside import ATP-binding protein	
C_6	mglB	D-galactose-binding periplasmic protein	Galactose
	mglC	Galactoside transport system permease protein	
	manX	PTS system mannose-specific EIIAB component	
C_6	manY	PTS system mannose-specific EIIC component	D-Mannose
	manZ	PTS system mannose-specific EIID component	
C_6	fucP	L-fucose-proton symporter	L-fucose
	alsA	D-allose import ATP-binding protein	
C_6	alsB	D-allose-binding periplasmic protein	D-allose
	alsC	D-allose transport system permease protein	
C_{12}	lacY	Lactose permease	Lactose
	malE	Maltose/maltodextrin-binding periplasmic protein	
C_{12}	malF	Maltose/maltodextrin transport system permease protein	Maltose
	malG	Maltose/maltodextrin transport system permease protein	
	malK	Maltose/maltodextrin import ATP-binding protein	
C_{12}	malX	PTS system maltose-specific EIICB component	Maltose
C_{12}	treB	PTS system trehalose-specific EIIBC component	Trehalose
C_{12}	melB	Melibiose carrier protein	Melibiose
	srlA	PTS system glucitol/sorbitol-specific EIIC component	
C_6 (polyol)	srlB	PTS system glucitol/sorbitol-specific EIIA component	Sorbitol
	srlE	PTS system glucitol/sorbitol-specific EIIB component	
	mtlA	PTS system mannitol-specific EIICBA component	
C_6 (polyol)	cmtA	PTS system mannitol-specific cryptic EIICB component	Mannitol
	cmtB	Mannitol-specific cryptic phosphotransferase enzyme IIA component	

c

C_n	Gene	Protein	Substrate
C_3	glpF	Glycerol uptake facilitator protein	Glycerol
C_5	rbsC	Ribose import permease protein	D-Ribose
C_5	araH	L-arabinose transport system permease protein	L-arabinose
C_5	xylH	Xylose transport system permease protein	D-xylose
C_6	mglC	Galactoside transport system permease protein	Galactose
C_6	alsC	D-allose transport system permease protein	D-allose
C_{12}	malG	Maltose/maltodextrin transport system permease protein	Maltose

d

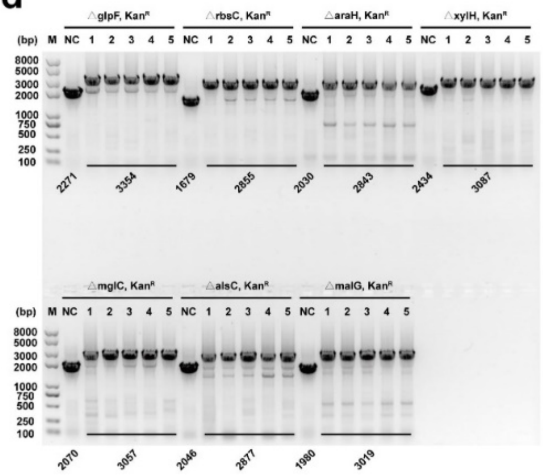

Supplementary Figure 34. Potential endogenous erythritol transporter homologous screening.
(**a**) Schematic diagram of screening *E. coli* MG1655 native transporters that might facilitate erythritol transportation. (**b**) List of all carbohydrate transportation genes and proteins in *E. coli* MG1655. (**c**) Screened seven carbohydrate transporter-associated genes. glpF has been reported that is able to transport erythritol. The other six genes are somewhat homologous to erythritol ABC transporter gene eryG by BLAST protein-protein alignment analysis. (**d**) Construction of mutated *E. coli* MG1655 strains by Lambda-Red recombination. In each of the seven mutated strains, “NC” represents the wild-type *E. coli* MG1655 genome PCR product by the primer pair “forward left-homologous-arm” and “reverse right-homologous-arm”. The other five PCR products are the desired mutation strains by the same primer pair. The KanR genes are not deleted by pCP20.

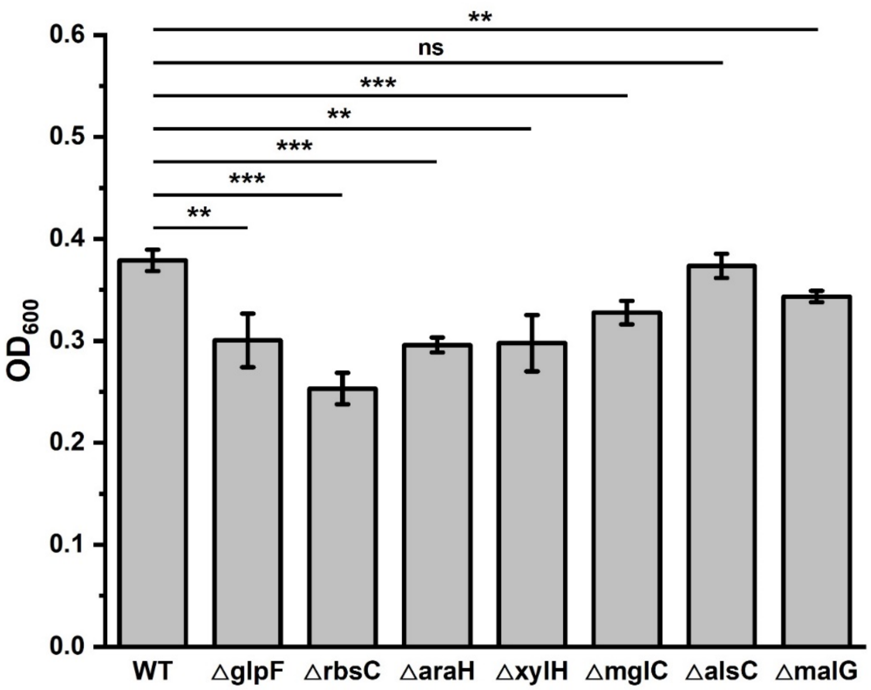

Supplementary Figure 35. Several eryG homologous permeases in *E. coli* may facilitate erythritol catabolism. BLAST protein-protein alignment results showed that seven *E. coli* native carbohydrate ABC-transporter permeases are homologous to erythritol permease eryG. We then constructed 7 different knock-out *E. coli* MG1655 strains by Lambda-Red recombination. All these 7 strains and the WT (wild-type *E. coli* MG1655) strain were transformed with pFB147 to utilize erythritol. Then, all strains were incubated (M9-erythritol medium, 37°C, no shaking) in 96-well plates for 72 h (stationary phase). OD_{600} values were measured at each 12 h. The final OD_{600} values at 72 h were shown in the graph. All values were measured as three biological replicates. The error bars represent the standard deviation (s.d.). Student's *t*-tests are used for statistical analysis and $p < 0.05$ indicates statistical significance (**$p < 0.01$ and ***$p < 0.001$, ns, no significance).

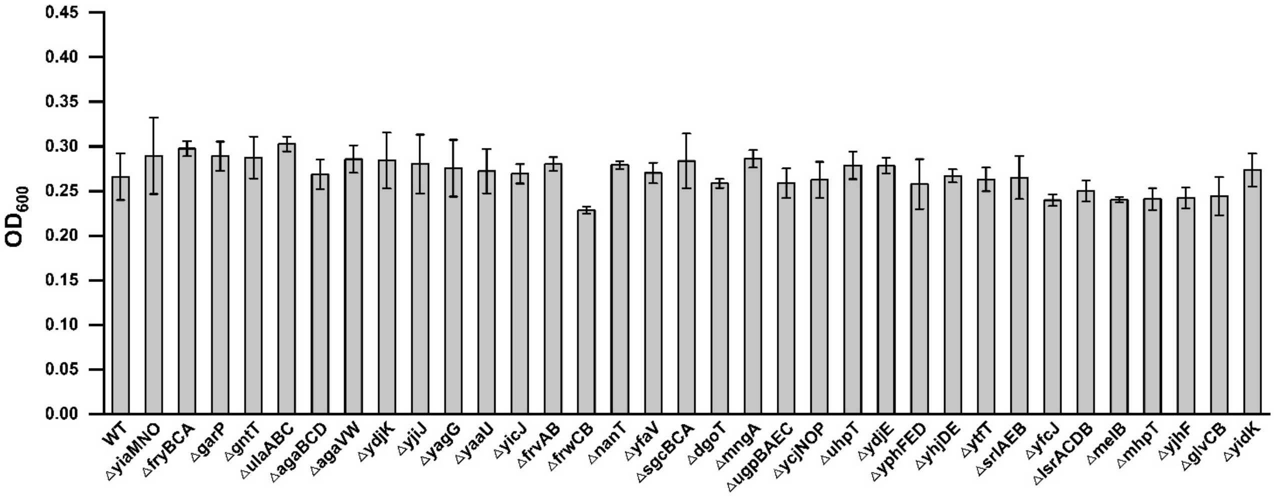

Supplementary Figure 36. Up-regulated carbohydrate transporters were not essential for erythritol transport (from COG analysis of RNA-seq).

COG analysis of RNA-seq indicated totally 34 up-regulated genes that might associate with “carbohydrate transport”. All these genes (or gene clusters) were referred and we constructed 34 different knock-out *E. coli* MG1655 strains by Lambda-Red recombination[3]. All these 34 strains and WT (wild-type *E. coli* MG1655) strain were then transformed with pFB147 to utilize erythritol. After that, all strains were incubated (M9-erythritol medium, 37°C, no shaking) in 96-well plates for 108 h (stationary phase). OD_{600} values were measured each 12 h. This graph showed the final OD_{600} values at 108 h. All values were measured as three biological replicates. The error bars represent the standard deviation (s.d.). Student’s *t*-tests are used for all the 34 genes’ statistical analysis. Overall, there were no statistical significance ($p > 0.05$) between all the 34 measurements, which means that all the 34 genes were not essential for erythritol transport.

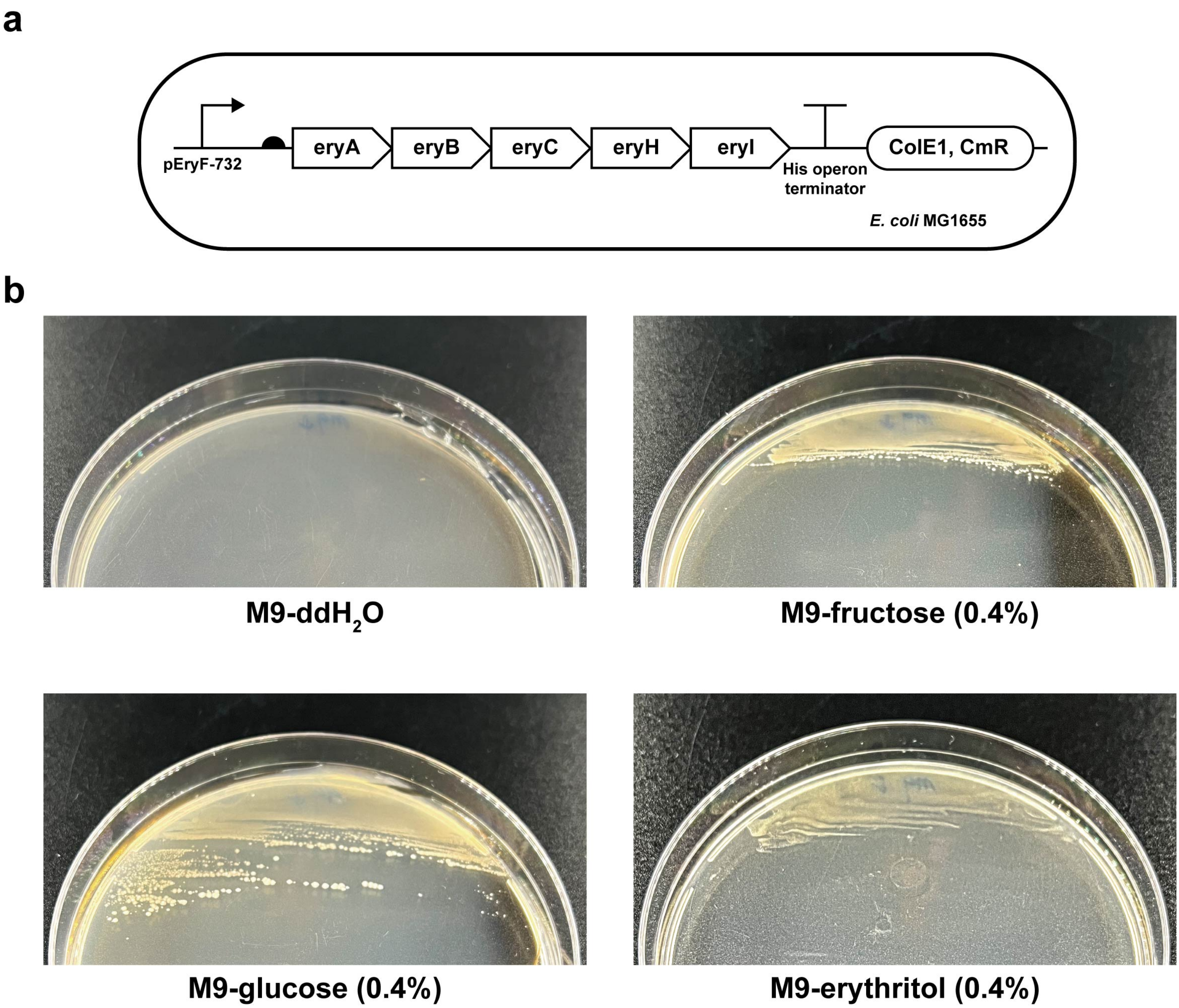

Supplementary Figure 37. Erythritol catabolic *E. coli* strain utilizes different carbon sources.
(**a**) Schematic diagram of *E. coli* MG1655 strain with the plasmid pFB147.
(**b**) The strain was incubated overnight in LB medium at 37°C and 250 rpm for 16 h. On the second day, cell pellets were washed with 1x phosphate-buffered saline (pH 7.4) for three times, then the suspension was spread onto different "M9-carbon source" agar plates and incubated at 37°C for 72 h. This experiment indicated that such "erythritol catabolic *E. coli* strain" could utilize fructose or glucose or erythritol as sole carbon source.

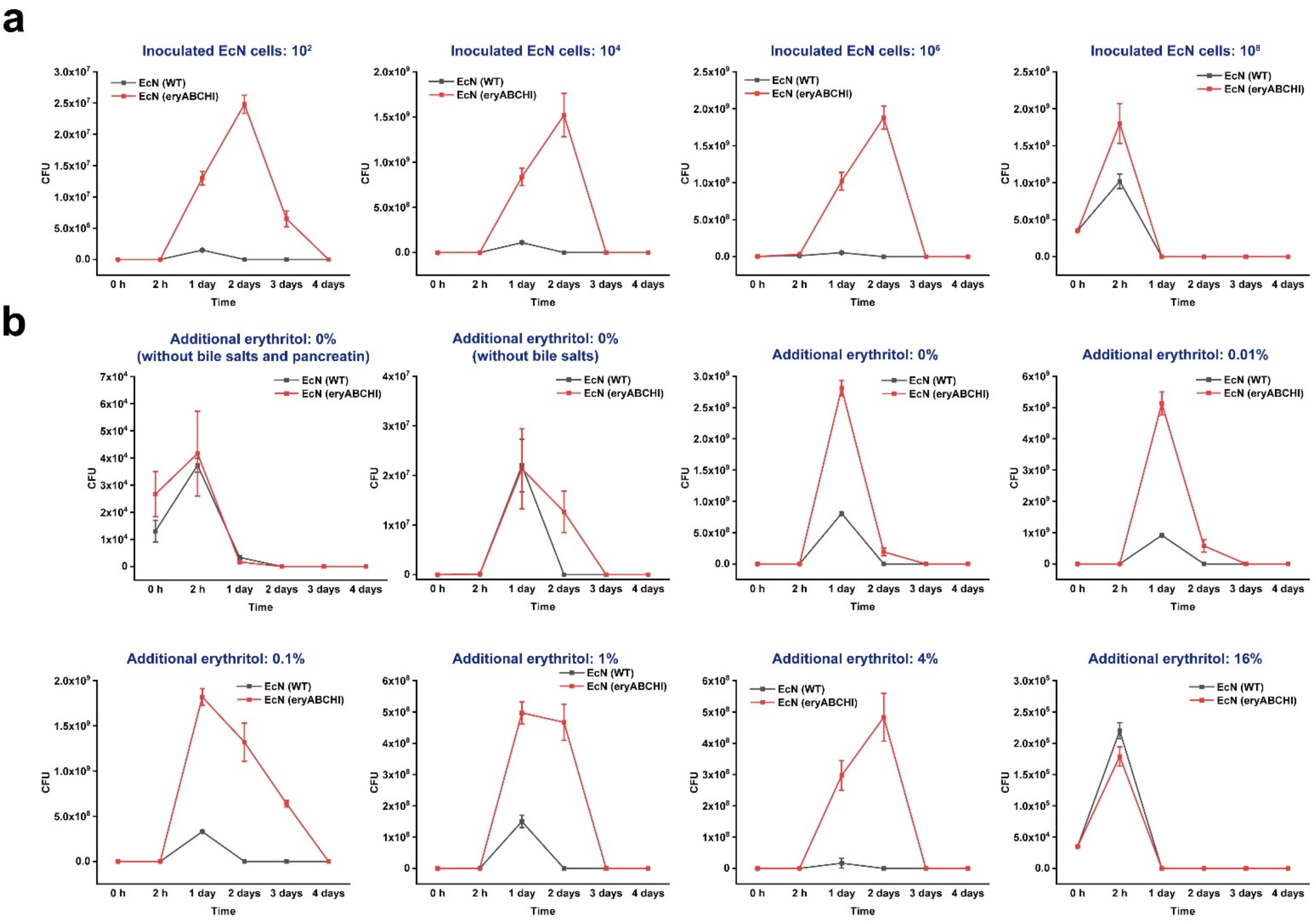

Supplementary Figure 38. Growth curves of engineered *E. coli* Nissle 1917 in SIF with additional erythritol.

(**a**) Growth curves with different inoculated cells of 10^2, 10^4, 10^6, and 10^8, respectively. Additional erythritol was added as 0.4% in SIF.

(**b**) Growth curves with different additional erythritol concentrations of 0%, 0.01%, 0.1%, 1%, 4%, and 16% in SIF, respectively. Two control experiments were performed for comparison -one without bile salts and pancreatin and the other one without bile salts. All the initial inoculated cells were the same with 10^4. All the measurements were performed with three biological replicates. The error bars represent the standard deviation (s.d.).

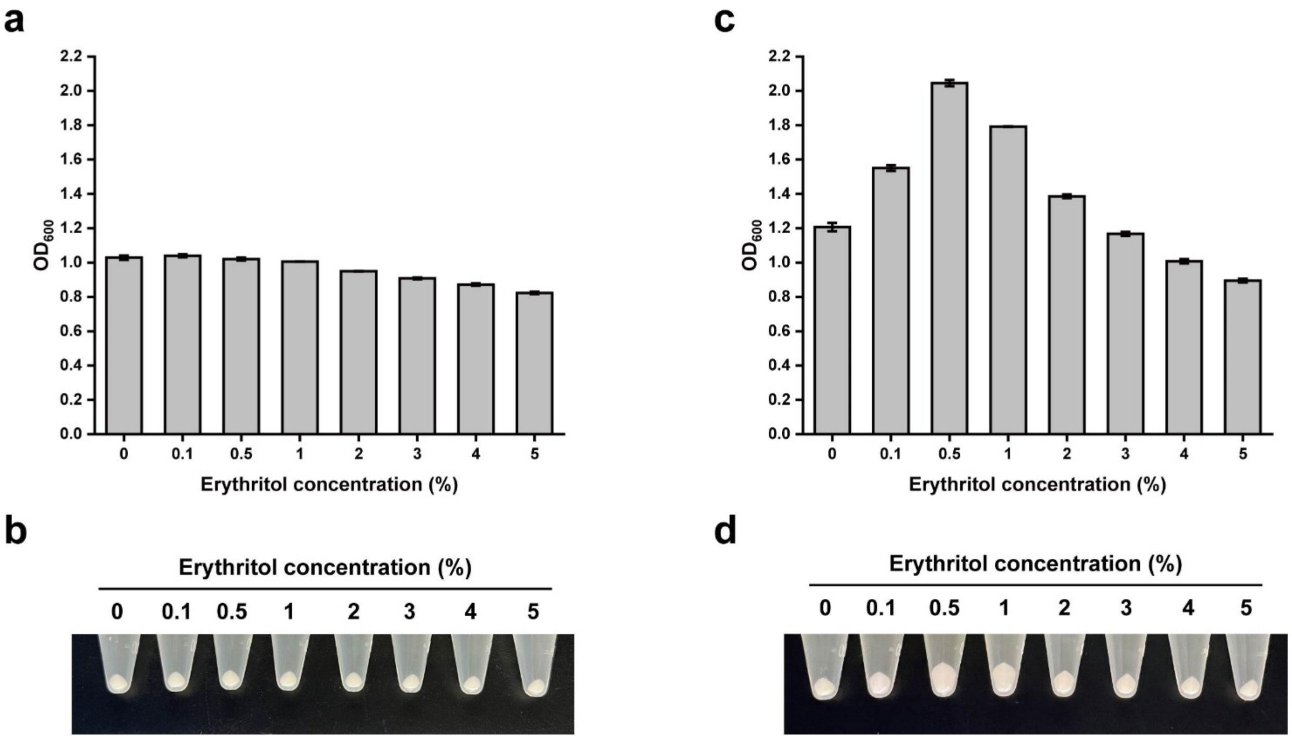

Supplementary Figure 39. Additional erythritol catabolism promotes *E. coli* growth in LB medium.
(**a**) Wild-type *E. coli* MG1655 without plasmids was incubated in 5 mL LB medium (without antibiotics). Additional erythritol was added to LB medium and then the cells were cultivated at 37°C, 250 rpm for 16 h. On the second day, the final OD_{600} values were measured. The results showed that erythritol could not promote *E. coli* growth without the erythritol catabolic cluster.
(**b**) The same samples as described in (**a**). 1.5 mL different cell cultures were collected and centrifuged in 1.5 mL tubes. Cell pellets were accumulated at the tube bottom.
(**c**) *E. coli* MG1655 with plasmid pFB147 (erythritol catabolic cluster) was incubated in 5 mL LB medium (with 34 µg/mL chloramphenicol). Additional erythritol was added to LB medium and then the cells were cultivated at 37°C, 250 rpm for 16 h. On the second day, the final OD_{600} values were measured. The results showed that erythritol could promote *E. coli* growth with the erythritol catabolic cluster, and the optimal erythritol concentration was 0.5% (w/v).
(**d**) The same samples as described in (**c**). 1.5 mL different cell cultures were collected and centrifuged in 1.5 mL tubes. Cell pellets were accumulated at the tube bottom.

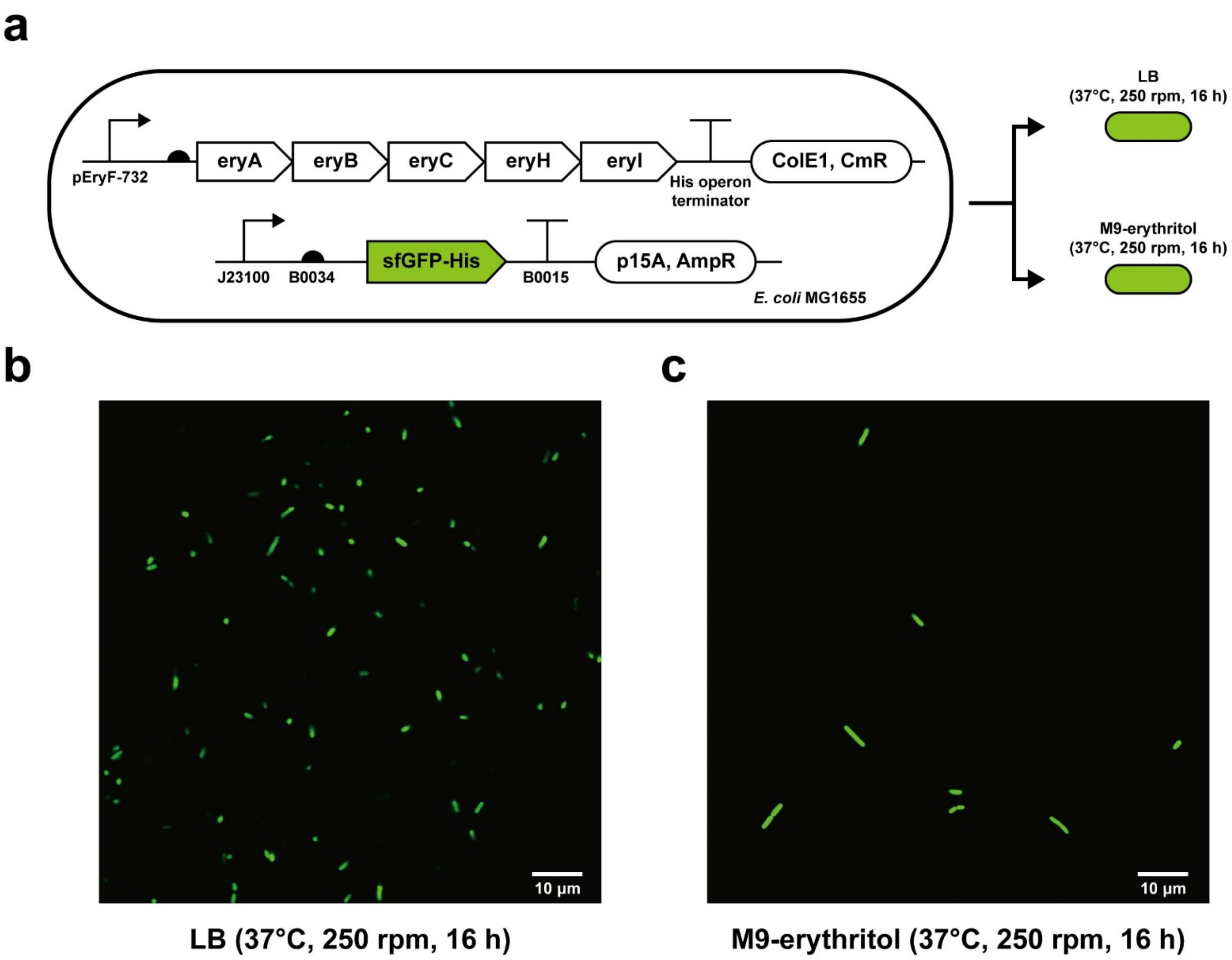

Supplementary Figure 40. *E. coli* MG1655 with the erythritol catabolic cluster could express green fluorescent protein in M9-erythritol medium.

(**a**) Schematic diagram of the engineered strain and plasmids (pFB147 and pFB285). This experiment aimed to test whether the *E. coli* strain with the erythritol catabolic cluster could express other heterologous proteins (e.g., sfGFP) in M9-erythritol medium. The same strain was incubated with both LB medium and M9-erythritol medium, respectively. After overnight cultivation, cell cultures were observed and imaged under confocal laser scanning microscopy.

(**b**) Confocal images of two cell cultures. The imaging parameters of the two samples were the same. We observed that in both cell cultures, sfGFP could be expressed and each cell showed a similar level of fluorescence intensity.

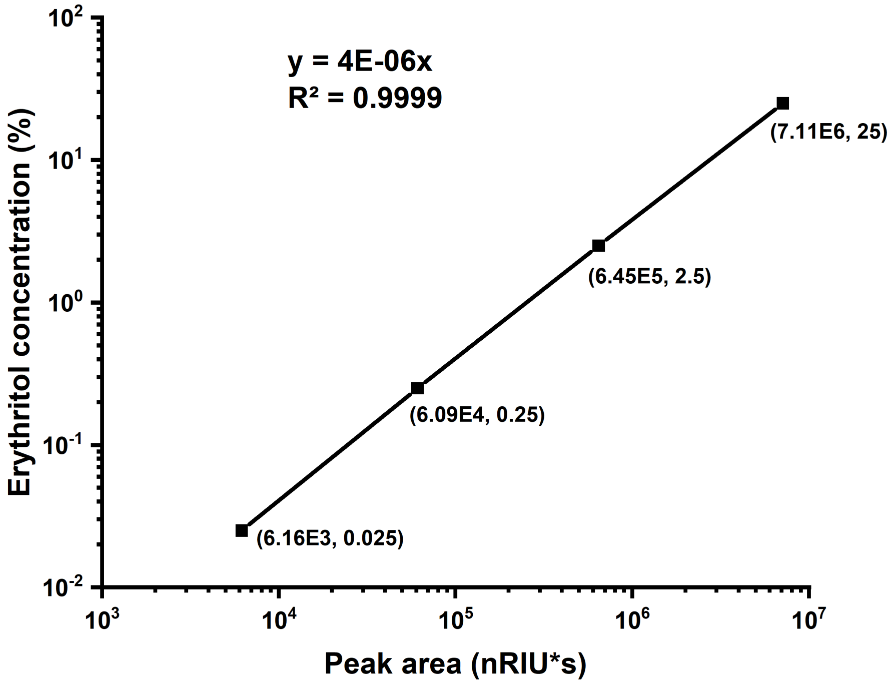

Supplementary Figure 41. Standard curve of erythritol analyzed by HPLC (Refractive Index Detector, RID). Each point was measured and calculated as an average value in triplicates.

III. Supplementary References

References
[1] *Registry of Standard Biological Parts*, <http://parts.igem.org/Main_Page?title=Main_Page> (2022).
[2] Ba, F., Liu, Y., Liu, W. Q., Tian, X., & Li, J. SYMBIOSIS: synthetic manipulable biobricks via orthogonal serine integrase systems. *Nucleic Acids Res.* **50**, 2973–2985 (2022).
[3] Datsenko, K. A. & Wanner, B. L. One-step inactivation of chromosomal genes in *Escherichia coli* K-12 using PCR products. *Proc. Natl. Acad. Sci. U.S.A.* **97**, 6640–6645 (2000).
[4] Altschul, S. F., Madden, T. L., Schäffer, A. A., Zhang, J., Zhang, Z., Miller, W., & Lipman, D. J. Gapped BLAST and PSI-BLAST: a new generation of protein database search programs. *Nucleic Acids Res*. **25**, 3389–3402 (1997).
[5] Johnson, M., Zaretskaya, I., Raytselis, Y., Merezhuk, Y., McGinnis, S., & Madden, T. L. NCBI BLAST: a better web interface. *Nucleic Acids Res*. **36**, W5–W9 (2008).
[6] Krogh, A., Larsson, B., von Heijne, G., & Sonnhammer, E. L. Predicting transmembrane protein topology with a hidden Markov model: application to complete genomes. *J. Mol. Biol*. **305**, 567–580 (2001).
[7] Reese M. G. Application of a time-delay neural network to promoter annotation in the *Drosophila melanogaster* genome. *Comput. Chem*. **26**, 51–56 (2001).
[8] Jumper, J. et al. Highly accurate protein structure prediction with AlphaFold. *Nature* **596**, 583–589 (2021).
[9] Varadi, M. et al. AlphaFold Protein Structure Database: massively expanding the structural coverage of protein-sequence space with high-accuracy models. *Nucleic Acids Res*. **50**, D439–D444 (2022).
[10] Kim, D. E., Chivian, D., & Baker, D. Protein structure prediction and analysis using the Robetta server. *Nucleic Acids Res*. **32**, W526–W531 (2004).
[11] Waterhouse, A. et al. SWISS-MODEL: homology modelling of protein structures and complexes. *Nucleic Acids Res*. **46**, W296-W303 (2018).
[12] Bienert, S., Waterhouse, A., de Beer, T. A., Tauriello, G., Studer, G., Bordoli, L., & Schwede, T. The SWISS-MODEL Repository-new features and functionality. *Nucleic Acids Res*. **45**, D313-D319 (2017).
[13] Blattner, F. R. et al. The complete genome sequence of *Escherichia coli* K-12. *Science* **277**, 1453–1462 (1997).
